# High Resolution Quantitative Trait Locus Mapping and Whole Genome Sequencing Enable the Design of an *Anthocyanidin Reductase*-Specific Homoeo-Allelic Marker for Fruit Colour Improvement in Octoploid Strawberry (*Fragaria × ananassa*)

**DOI:** 10.3389/fpls.2022.869655

**Published:** 2022-03-18

**Authors:** Marc Labadie, Guillaume Vallin, Aline Potier, Aurélie Petit, Ludwig Ring, Thomas Hoffmann, Amèlia Gaston, Juan Munoz-Blanco, José L. Caballero, Wilfried Schwab, Christophe Rothan, Béatrice Denoyes

**Affiliations:** ^1^Université de Bordeaux, INRAE, UMR BFP, Villenave d’Ornon, France; ^2^Invenio, MIN de Brienne, Bordeaux, France; ^3^Biotechnology of Natural Products, Technical University of Munich, Freising, Germany; ^4^Departamento de Bioquímica y Biología Molecular, Universidad de Córdoba, Córdoba, Spain

**Keywords:** *Fragaria × ananassa*, colour, anthocyanins, mQTL, *F. vesca* subgenome, homoeo-allele, *ANR*, MYB-like ODORANT

## Abstract

Fruit colour is central to the sensorial and nutritional quality of strawberry fruit and is therefore a major target in breeding programmes of the octoploid cultivated strawberry (*Fragaria × ananassa*). The red colour of the fruit is caused by the accumulation of anthocyanins, which are water-soluble flavonoids. To facilitate molecular breeding, here we have mapped with high resolution fruit colour quantitative trait loci (QTLs) (COLOUR, scored visually as in selection programmes) and associated flavonoid metabolic QTLs (5 anthocyanins compounds together with 8 flavonols and flavan-3-ols) to specific subgenomes of cultivated strawberry. Two main colour-related QTLs were located on the LG3A linkage group (*Fragaria vesca* subgenome). Genetic mapping, transcriptome analysis and whole genome sequencing enabled the detection of a homoeo-allelic variant of *ANTHOCYANIDIN REDUCTASE* (*ANR)* underlying the major male M3A COLOUR and pelargonidin-3-glucoside (PgGs) QTLs (up to ∼20% of explained variance). Consistent with previously published functional studies, *ANR* transcript abundance was inversely related with PgGs content in contrasted progeny individuals. Genetic segregation analyses further indicated that a molecular marker designed using an 18 bp deletion found in the 5′UTR of the candidate *ANR* homoeo-allelic variant is effective in identifying genotypes with intense red fruit colour. Our study provides insights into the genetic and molecular control of colour-related traits in strawberry and further defines a genetic marker for marker-assisted selection of new strawberry varieties with improved colour. The QTLs detected and the underlying candidate genes are different from those described to date, emphasising the importance of screening a wide diversity of genetic resources in strawberry.

## Introduction

**FIGURE 1:**
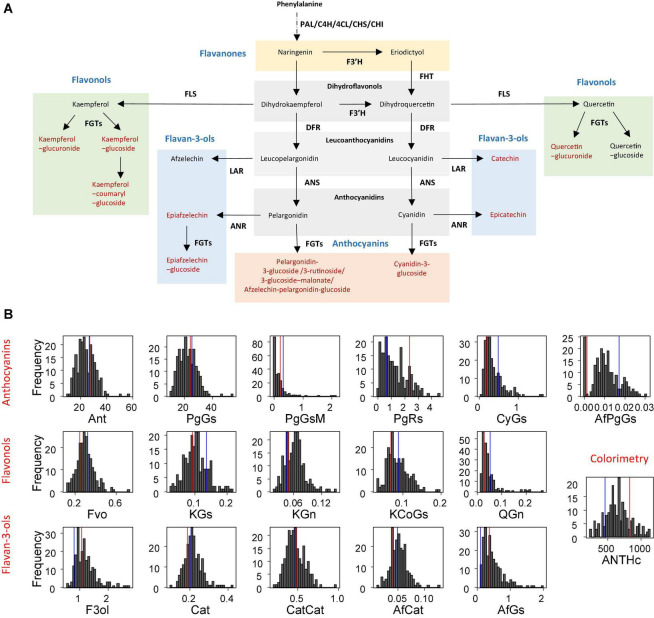
Flavonoids of strawberry (*Fragaria × ananassa*) fruit and their distribution in the progeny. **(A)** Simplified flavonoid biosynthetic pathway. Compounds assessed in this study are in red. Chemical classes are in blue. ANR, anthocyanidin reductase; ANS, anthocyanidin synthase; CHI, chalcone isomerase; C4H, cinnamic acid-4-hydroxylase; 4CL, 4-coumarate:CoA ligase; CHS, chalcone synthase; DFR, dihydroflavonol-4-reductase; FGT, UDPglucose: flavonoid-3-O-glucosyltransferase; FHT/F3H, flavanone-3-hydroxylase; FLS, flavonol synthase; LAR, leucoanthocyanidin reductase; PAL, phenylalanine ammonia-lyase. **(B)** Distribution of the progeny mean in 2010. The mean phenotypic values of the parents are shown in red for “Capitola” and in blue for “CF1116.” Ant, Fvo, F3ol values were obtained by summation of total anthocyanins, total flavonols and total flavan-3-ols, respectively; PgGs, pelargonidin-3-glucoside; PgGsM, pelargonidin-3-glucoside-malonate; PgRs, pelargonidin-3-rutinoside; CyGs, cyanidin-3-glucoside; AfPgGs, (epi)afzelechin-pelargonidin-3-glucoside; KGs, kaempferol-glucoside; KGn, kaempferol-glucuronide; KCoGs, kaempferol-coumaryl-glucoside; QGn, quercetin-glucuronide; Cat, catechin; CatCat, (epi)catechin dimers; AfCat, (epi)afzelechin-(epi)catechin dimers; AfGs, (epi)afzelechin-glucoside; ANTHc, anthocyanins (colourimetry). The flavonoid metabolites values are expressed as mg-equ/100 g FW assuming a response factor of 1. ANTHc results are expressed as mg pelargonidin-3-glucoside equivalents/100 g FW.

The cultivated strawberry (*Fragaria × ananassa*) is the most consumed small fruit worldwide. Since its creation in the 18th century in botanical gardens in Europe by fortuitous hybridisation between the two New World strawberry species *Fragaria chiloensis* and *Fragaria virginiana* (Edger et al., 2019), *F. × ananassa* has been continuously improved to fit the needs of both producers and consumers. In recent years, fruit sensorial quality including fruit colour has become a major target for strawberry breeding (Mezzetti et al., 2018). The red fruit colour is due to the accumulation of the anthocyanin pigments, which are water-soluble flavonoids. The flavonoids detected in strawberry fruit (anthocyanins, flavonols, and flavan-3-ols) (Ring et al., 2013; Urrutia et al., 2016; Davik et al., 2020; Labadie et al., 2020; Pott et al., 2020) are derived from the phenylpropanoid pathway (Figure 1A). Flavonols and flavan-3-ols are mainly glycosides of quercetin and kaempferol as well as derivatives of catechin and epicatechin. Anthocyanins are mainly glycosides of pelargonidin and cyanidin, whose composition gives the fruit its distinctive colour hue, from bright red (pelargonidin derivatives) to dark red (cyanidin derivatives). In addition, anthocyanins are antioxidant molecules with proven dietary health-benefits (Butelli et al., 2008; Tulipani et al., 2008) and make a major contribution to the nutritional quality of strawberries (Battino et al., 2009; Giampieri et al., 2014; Miller et al., 2019).

In a recent study of a pseudo F1 progeny of *F. × ananassa* using SSR markers (Labadie et al., 2020), we mapped flavonoid metabolic quantitative trait loci (mQTLs) and other colour- and antioxidant-related QTLs assessed through colourimetric assays and LC-ESI-MS analysis (Ring et al., 2013; Haugeneder et al., 2018). Additional flavonoid mQTLs were identified by linkage mapping and association studies of other bi-parental populations of *F. × anan*assa (Davik et al., 2020; Pott et al., 2020). Interestingly, most mQTLs mapped to different genomic regions depending on the population studied, indicating that a large genetic diversity is available in strawberry to improve fruit colour, which is a complex trait. Identifying the genetic variants underlying colour-related QTLs can help decipher the signalling or biosynthetic pathways responsible for natural variations in fruit colour and, moreover, enable the design of specific genetic markers for marker-assisted selection (MAS) of new strawberry varieties with improved fruit colour.

In the octoploid *F. × ananassa* (2*n* = 8x = 56), trait variation at a single locus may be controlled by up to eight homoeo-alleles located on four linkage groups (LGs) corresponding to the four subgenomes of *F. × ananassa*. Recent findings suggest that each subgenome is mainly derived from one of four ancestral diploid species *Fragaria vesca*, *Fragaria iinumae*, *Fragaria nipponica*, and *Fragaria viridis* with intrachromosomal patterns of mixed ancestral DNA variation (Edger et al., 2019; Hardigan et al., 2021a). The current resolution offered by the high-density strawberry SNP genotyping arrays (Bassil et al., 2015; Hardigan et al., 2020) now makes it possible to narrow a mQTL down to a small chromosomal region specific to a given subgenome (Hardigan et al., 2020, 2021a), as done for the identification of several malonyltransferase candidate genes underlying pelargonidin-3-O-malonylglucoside QTLs (Davik et al., 2020). Thanks to the availability of high quality genome sequences of the diploid woodland strawberry *F. vesca* (Shulaev et al., 2011; Edger et al., 2018) and octoploid *F. × ananassa* (Edger et al., 2019; Liston et al., 2020; Hardigan et al., 2021a) and progress in sequencing, it is now possible to obtain whole genome sequences (WGS) of the parents of the population studied, compare them to reference genomes, identify polymorphism in candidate genes and design specific markers for MAS. This strategy should further accelerate the discovery of homoeo-allelic variants underlying colour and flavonoid variations in *F. × ananassa*.

In this study focused on fruit colour QTLs and built on previous work from Labadie et al. (2020), we identify a deletion in the anthocyanidin reductase (*ANR)* gene that underlies a major colour QTL on LG LG3A and design a simple marker for COLOUR, a visual trait commonly scored in breeding programmes. To this end, we first built a new linkage map where LGs were classified according to their subgenome by using the Affymetrix IStraw90 Axiom array (Bassil et al., 2015). We further mapped with high-resolution flavonoid mQTLs (Labadie et al., 2020) and two new traits linked to fruit colour. The combination of mQTL mapping and transcriptome analysis of progeny individuals led to the identification of candidate genes underlying two major homoeo-mQTLs for pelargonidin-3-glucoside (PgGs) located on LG3A (*F. vesca* subgenome). Whole genome sequencing of the parents next allowed us to detect a specific homoeo-allelic deletion in *ANR* that underlies a male colour QTL. The *ANR* deletion can be used as a genetic marker for the prediction of *ANR* allelic status and therefore of fruit PgGs content and colour. These discoveries are important to further our knowledge of the control of anthocyanin biosynthesis in the fruit and to define the best strategy for breeding new strawberry varieties with improved colour and health benefits.

## Materials and Methods

### Plant Materials and Preparation

A pseudo full-sibling F_1_ population of 165 individuals obtained from a cross between the variety “Capitola” (“CA75.121-101” × “Parker,” University of California, Davis, CA, United States) and the advanced line “CF1116” {[“Pajaro” × (“Earlyglow” × “Chandler”)], reference from the Ciref, France} was developed. The “Capitola” and “CF1116” parents display contrasting fruit colour together with differences in fruit shape and weight, firmness, sweetness, and acidity (Lerceteau-Köhler et al., 2012). For each of the two consecutive study years (2010 and 2011), cold-stored strawberry plants planted in 2009 and 2010 were grown in soil-free pine bark substrate under plastic tunnel with daily ferti-irrigation and control of biotic stresses. The mapping population included a total of 165 individuals over the two study years. Within this progeny, 72 and 131 individuals, including the parents, were respectively phenotyped in 2010 and in 2011. Fruits were harvested at the red ripe stage, when red colouration of the fruit is homogeneous, and processed as previously indicated (Labadie et al., 2020) to produce frozen powder samples that were further stored at −80°C until use for chemical analyses.

### Fruit Colour Evaluation and Flavonoid Chemical Analyses

In 2011, for both harvests, red ripe fruits (4--5 fruits per harvest) were also photographed side-by-side with the strawberry colour chart from Ctifl.^1^ Fruit colour was then scored by two independent persons (two replicates) on a scale from 0 (very pale red-orange) to 6 (very dark red) using the photographs. A mean score value was then obtained for each genotype (Supplementary Figure 1).

Analysis of polyphenolic metabolites by LC–ESI-MS was done as previously described (Ring et al., 2013). A total of 16 traits encompassing 13 individual phenolic metabolites and their sum by chemical class (three traits) were measured for the 2 years as described in Labadie et al. (2020). Analyses of pooled frozen powder samples were carried out in 2010 and 2011 on six replicates for the parents and on three replicates for individuals from the progeny.

Extraction and measurement of total anthocyanin content (ANTHc) by colourimetric assay were as described in Labadie et al. (2020). Results are expressed as mg PgGs equivalents/100 g fresh weight. For each genotype, four technical repeats from the pooled two-harvest-fruit-powder were performed.

Frequency distribution of each trait was represented using ggplot2 (Wickham, 2016) (v3.2.1) r-package.

### Genotyping

DNA from the parental lines “Capitola” and “CF1116” and from 165 individuals from the mapping population was extracted using DNeasy Plant Mini kit (Qiagen, Hilden, Germany) and hybridised with the Affymetrix^®^ 90 K Axiom^®^ SNP array (Affymetrix, CA, United States) at CeGen USC (Santiago de Compostela, Spain). IStraw90^®^ SNP array includes 138,000 SNP markers corresponding to 90,000 localisations on wild diploid strawberry *F. vesca* genome (Bassil et al., 2015; Edger et al., 2020). Analysis was performed using Genotyping console™ and SNPpolisher© (Affymetrix, CA, United States) following manufacturers recommendations.

We developed a molecular marker, named BxANR_5UTR, in the 5′UTR of the *ANR* gene. To this end, using the *F. vesca* reference genome and WGS sequencing data from both parents, “Capitola” and “CF1116,” we designed primers for BxANR_5UTR (forward: GAGAGTTGGTGGTGCTTTCA; reverse ATGGT GTGGGTGTGTCTCAG, 5′-3′) to amplify a 182 bp fragment that spans an 18 bp gap (TTCTTCCTCTTCTTCTTC) in the *ANR* 5′UTR. As described in Perrotte et al. (2016), we used specific extended primers of the M13 sequence which allow the hybridisation of a fluorolabelled primer (6-FAM or VIC). The PCR products of all individuals of the segregating population were diluted 1:200 and separated using capillary electrophoresis [CE; Applied Biosystems (ABI 3730), ABI Genescan™ 500 LIZ size standard]. Peak identification and fragment sizing of each electropherogram were done with GeneMapper v4.0 software. Data were converted to a qualitative binary code for scoring the presence or absence of BxANR_5UTR homoeo-alleles.

### Linkage Maps and Quantitative Trait Locus Analysis

Single dose markers (SD) from the Affymetrix array (Bassil et al., 2015) that were in backcross configuration and segregated 1:1 (Rousseau-Gueutin et al., 2008) were used in combination with previously mapped SSR, SSCP, and AFLP markers (Gaston et al., 2013; Labadie et al., 2020) for map construction using JoinMap 5.1 software (Van Ooijen, 2011). Grouping was performed using independence log of the odds (LOD) and the default settings in JoinMap^®^. LGs were chosen from an LOD higher than 10 for all of them. Map construction was performed using the maximum likelihood (ML) mapping algorithm and the following parameters: chain length 5.000, initial acceptance probability 0.250, cooling control parameter 0.001, stop after 30.000 chains without improvement, length of burn-in chain 10.000, number of Monte Carlo EM cycles 4, chain length per Monte Carlo EM cycle 2.000 and sampling period for recombination frequency matrix samples: 5.

For QTL analysis, the female and male linkage parental maps based on the 165 individuals were used separately. Phenotypic data of the 72 and 131 individuals for 2010 and 2011, respectively, were represented by the mean value of the three replicates. QTL detection was performed by simple interval mapping (SIM) using R/QTL (Broman et al., 2003). Permutation analysis (1,000 permutations) was performed to calculate the critical LOD score. QTL with LOD values higher than the LOD threshold at *P* ≤ 0.05 were considered significant. When one QTL was found significant, we used composite interval mapping (CIM) with one co-variable at the position of the significant QTL and reiterated the analysis until no new significant QTLs were detected. Bayesian credible interval was calculated using the function “bayesint” at probability of 0.95. The proportion of phenotypic variance explained by a single QTL was calculated as the square of the partial correlation coefficient (*R*^2^). Mapping results are displayed using MapChart (Voorrips, 2002).

### Gene Expression Analysis

A custom-made oligonucleotide-based (60-mer length) platform (Roche NimbleGen) designed from non-redundant *F. vesca* strawberry sequences (Fv_v1.0, Shulaev et al., 2011) representing a total of 18,152 unigenes (Ring et al., 2013) was used for the analysis of differentially expressed genes between 21 F1 genotypes from the “Capitola” × “CF1116” population displaying contrasted phenotypic values for flavonoids and colour-related traits. RNA extraction from red ripe fruits and microarray processing and data analysis were as previously described (Ring et al., 2013). Student’s *t*-test was used with a confidence of *P* < 0.05 to detect statistically significant differences.

### Whole Genome Sequencing

Whole genome sequencing of the two parents “Capitola” and “CF1116” was performed using paired-end Illumina sequencing with a ∼ 50X coverage of the *F. × ananassa* genome. Illumina paired-end shotgun-indexed libraries were prepared and sequenced using an Illumina HiSeq 3000 at the Institut National de la Recherche Agronomique GeT-PlaGe facility (Toulouse, France), operating in a 150-bp paired-end run mode. Raw fastq files were mapped to the *F. vesca* FvH4 reference genome sequence (*F. vesca* Genome v4.0.a1) (Edger et al., 2018) using BWA-MEM algorithm (Li, 2013) for the alignment of paired-end (150 bp) Illumina reads. Polymorphisms between “Capitola” and “CF1116” were identified using Integrative Genomics Viewer (IGV) (Robinson et al., 2011). All identified polymorphisms were tested for the 1:7 (mutant allele:WT alleles) segregation ratio for goodness-of-fit to theoretical ratio (Chi-squared test) when considering the hypothesis that, out of the eight homoeo-alleles expected in the octoploid *F. × ananassa*, one single homoeo-allele (mutant allele) specific to “Capitola” or to “CF1116” controls the trait.

## Results

### High Resolution Mapping of Fruit Colour-Related Traits in Strawberry

In addition to the thirteen fruit flavonoid compounds previously analysed by LC-ESI-MS (Labadie et al., 2020; Figure 1A), we mapped the colour of the fruit (COLOUR trait) scored visually according to the strawberry colour chart (Ctifl^2^ (Supplementary Figure 1). As described in Labadie et al. (2020), the flavonoids detected in the fruit were flavonols (four compounds), flavan-3-ols (four compounds), and anthocyanins [five compounds: PgGs, pelargonidin-3-glucoside-malonate (PgGsM), pelargonidin-3-rutinoside (PgRs), cyanidin-3-glucoside (CyGs), and (epi)afzelechin-pelargonidin-glucoside (EpPgGs)]. The quantitative value and range in parents and progeny, the broad sense heritability and the transgression value in the “Capitola” × “CF1116” population were reported in Labadie et al. (2020) for all the traits except COLOUR that was measured only in 2011 (Supplementary Table 1 and Supplementary Figure 1). Very high broad sense heritability values ranging from 0.81 (PgGs) to 0.93 (PgGsM) were observed for the major anthocyanin compounds (Labadie et al., 2020). Distributions of flavonoids and colour-related traits are shown in Figure 1B for 2010 and in Supplementary Figure 2 for 2011. Continuous variations of the phenotypic values were observed in the progeny for all the traits assessed in 2010 and in 2011.

In this study, we focused on colour-related mQTLs and QTLs. The PgGs, which contributes to total ANTHc for as much as ∼90%, displayed a fivefold variation among the most contrasted individuals. This is in agreement with the distribution of most flavonoid metabolites and colour-related traits in the progeny, which showed a 4–10-fold variation. As could be expected, the ANTHc values measured by colourimetry varied in the same range as PgGs. In contrast, considerable variations were observed for the minor anthocyanin forms CyGs and PgRs, which displayed 23-fold and 114-fold variations in the progeny, respectively; and for the COLOUR score, which ranged from 0.5 to 6. PgGs -malonate and CyGs were not detected in a large number of individuals, resulting in a very skewed distribution in the progeny (Figure 1B and Supplementary Figure 2).

For linkage map construction, we added to the previous linkage maps (Rousseau-Gueutin et al., 2008) 9,455 SNP markers from the Axiom^®^ IStraw90^®^ SNP array [only single dose markers (SD) with 4,974 female and 4,481 male markers] (Bassil et al., 2015). The construction of the linkage maps was done with a total of 5,216 and 4,879 markers for the female and male linkage maps, respectively. The final number of markers covered the expected 28 LGs for the female map and 31 LGs for the male map (3 additional small LGs were included) (Supplementary Tables 2–4). The lengths of the female and male linkage maps were 4,135.1 and 3,929.2 cM, respectively, with an average distance between markers of 0.8 cM.

Linkage groups were assigned to one of the seven homoeologous groups (HGs). However, contrary to our previous reports (Rousseau-Gueutin et al., 2008; Labadie et al., 2020), a homoeologous LG (e.g., LG1A, B, C, or D) has been named according to the recent nomenclature of Hardigan et al. (2020, 2021a) where letters refer to species-derived subgenomes: A, *F. vesca*; B, *F. iinumae*; C, *F. nipponica*, and D, *F. viridis* (Supplementary Table 2).

### The *Fragaria vesca* Derived Subgenome Is Predominant in the Genetic Architecture of Fruit Colour in the Population of *Fragaria × ananassa* Analysed

QTL were detected for all the quantitative traits analysed using CIM for “Capitola” or “CF1116.” Information on markers for the male and female maps are provided in Supplementary Tables 3, 4. The list of significant QTLs detected for each trait including associated markers, position on the male and female linkage maps, LOD score and effect is provided in Supplementary Table 5. The QTL values at the 5 and 10% thresholds used to select the significant male and female QTLs for each trait and year are given in Supplementary Table 6. Distributions per HGs and LGs of significant colour-related mQTLs [PgGs, PgGsM, PgRs, CyGs, EpPgGs, and Ant (summation of anthocyanins compounds)] and QTLs (ANTHc and COLOUR) are synthesised in Table 1 for the male and female. The graphical representation of the location on the male and female linkage maps for the significant QTLs detected for the 2 years of study (2010 and 2011) is presented in Supplementary Figure 3.

**TABLE 1:** Distribution and number of significant quantitative trait loci (QTLs) detected for colour-related traits according to the male and female linkage groups (LGs), year and subgenomes.

LGs-new-name^a^	LGs-old-name^a^	Nb QTLs in 2010	Nb QTLs in 2011	Total Nb of QTLs	Nb regions with QTLs	QTLs located on a same linkage group^b^
M1B	M1a		6	6	3	M_2011_COLOUR/2011_AfPgGs/2011_Ant; M_2011_PgGs/2011_PgRs;
						M_2011_ANTHc
M1C	M1c		1	1	1	M_2011_PgGsM
M2A/F2A	M2a/F2a	2	1	3	2	M_2011_AfPgGs; F_2010_Ant/2010_PgGs
**M3A/F3A**	**M3a/F3a**	**2**	**3**	**5**	**1**	**M_2011_COLOUR/2010_Ant/2010_PgGs/F_2011_Ant/2011_PgGs**
F3D	F3b		1	1	1	F_2011_PgGs
M4A	M4a	1		1	1	M_2010_ANTHc
M4D/F4D	M4d/F4d		2	2	2	M_2011_PgGs; F_2011_COLOUR
M5A	M5a		1	1	1	M_2011_PgRs
M5C	M5b		1	1	1	M_2011_PgRs
M6A/F6A	M6a/F6a	2	8	10	5	M_2011_PgRs; M_2011_ANTHc; F_2010_Ant/2010_PgGs/2011_COLOUR/2011_CyGs; F_2011_Ant/2011_ANTHc/2011_PgGs; F_2011_PgGsM
M6C	M6d		1	1	1	M_2011_PgRs
M6D/F6D	M6b/F6b		2	2	2	M_2011_PgRs; F_2011_AfPgGs
M7B	M7a		1	1	1	M_2011_PgRs
M7C	M7d		1	1	1	M_2011_PgRs
M41	M41		1	1	1	M_2011_PgRs
Total	Total	7	30	37	24	
**Subgenome** ^c^						
A		7	13	20	10 (42%)	
B			7	7	4 (17%)	
C			4	4	4 (17%)	
D			5	5	5 (21%)	
Unknown			1	1	1 (3%)	
Total		7	30	37	24	

*^a^Linkage groups were assigned to one of the seven homoeologous groups (HGs) according to the recent nomenclature (new name) (Hardigan et al., 2021a). Names used in previous studies (Lerceteau-Köhler et al., 2012; Perrotte et al., 2016; Labadie et al., 2020) (old name) are also indicated.*

*^b^Colour-related traits considered: anthocyanins measured by LC-ESI-MS (Ant, PgGs, PgGsM, PgRs, GyGs, AfPgGs); total anthocyanins measured by colourimetry (ANTHc); and colour assessed visually (COLOUR). QTLs were identified using CIM analysis with LOD > LOD threshold at 10%. In bold, LG3A QTLs further investigated. QTLs overlapping within a given LG (i.e., with overlapped Bayesian intervals) are separated by a slash (/). QTLs found on different regions within a given LG are separated by a semicolon (). mQTLs are underlined.*

*^c^Capital letters refer to species-derived subgenomes: A, F. vesca; B, F. iinumae; C, F. nipponica, and D, F. viridis.*

Among the 65 QTLs detected for all the flavonoid and colour-related traits analysed (Supplementary Table 5), a total of 37 (57%) significant colour-related QTLs (29 anthocyanins mQTLs, 4 ANTHc, and 4 COLOUR QTLs) was detected (Tables 1, 2). More than half of the colour-related QTLs (54%) were located on the *F. vesca* subgenome. Most LGs harboured only one region controlling variations in colour-related traits, the notable exception being the LG6A carrying two male and three female regions with colour-related QTLs (Table 1). Quantitative variations of few anthocyanin compounds were controlled by mQTLs located on different homoeologous LGs within the same HG (Table 2). The only examples are those of PgGs QTLs located on the female LGs F3A and F3D in 2011 and of PgRs QTLs located on the male LGs M5A and M5C and M7B and M7C in 2011. Moreover, half of the colour-related QTLs detected on the male linkage map were located on only three LGs (M1B, M3A, and M6A) (11 QTLs out of 22). On the female linkage map, the overwhelming majority (12 QTLs out of 15) were located on only three LGs (F2A, F3A, and F6A). Remarkably, the above-mentioned LGs correspond to the *F. vesca* subgenome. Analysis of the contribution of the different subgenomes further highlighted the predominant role of *F. vesca* in the determination of fruit colour in *F. × ananassa*. The *F. vesca* subgenome is responsible for major variations in PgGs content (F2A QTL: *R*^2^ = 15; M3A QTL: *R*^2^ = 20.6; F3A QTL: *R*^2^ = 8.8; F6A QTL: *R*^2^ = 19.7) and contributes to 42% of the total number of colour-related QTLs detected (Table 1).

**TABLE 2:** Distribution of significant anthocyanin mQTLs and visually assessed COLOUR QTL in the male (M) and female (F) linkage maps.

Traits	Abbreviation	Nb of QTLs	Location of QTLs on linkage groups (years)^a^
Anthocyanins			
Total anthocyanins	Ant	6	M1B (2011), F2A (2010), M3A (2010), F3A (2011), F6A (2010 and 2011)
Pelargonidin-3-glucoside	PgGs	8	M1B (2011), F2A (2010), M3A (2010), F3A (2011), F3D (2011), M4D (2011), F6A (2010 and 2011)
Pelargonidin-3-glucoside-malonate	PgGsM	2	M1C (2011), F6A (2011)
Pelargonidin-3-rutinoside	PgRs	9	M1B (2011), M5A (2011), M5C (2011), M6A (2011), M6D (2011), M6C (2011), M7B (2011), M7C (2011), M41 (2011)
Cyanidin-3-glucoside	CyGs	1	F6A (2011)
(epi)Afzelechin-pelargonidin-glucoside	AfPgGs	3	M1B (2011), M2A (2011), F6D (2011)
Colourimetry			
Anthocyanins (colourimetry)	ANTHc	4	M1B (2011), M4A (2010), M6A (2011), F6A (2011)
Visual assessment			
Colour	COLOUR	4	M1B (2011), M3A (2011), F4D (2011), F6A (2011)

*^a^Capital letters refer to species-derived subgenomes: A, F. vesca; B, F. iinumae; C, F. nipponica, and D, F. viridis (Hardigan et al., 2021a). QTLs were identified using CIM analysis with LOD > LOD threshold at 10%.*

The *F. iinumae* (17%), *F. nipponica* (17%), and *F. viridis* (21%) subgenomes each contribute less than 25% of all 37 colour-related QTLs (Table 1). Subgenomes derived from *F. iinumae* (M1B QTL; *R*^2^ = 11.65) and from *F. viridis* (M3D and M4D QTLs; *R*^2^ > 7.5) contribute to variations of PgGs content in the progeny (Table 2 and Supplementary Table 5). *F. iinumae*, *F. nipponica*, and *F. viridis* are also involved in the control of PgRs and *F. viridis* in that of COLOUR (F4D; *R*^2^ = 9). We further investigated if the *MYB10-2* homoeo-allele located on the LG1 *F. iinumae*–derived subgenome (LG1-2, i.e., LG1B) (Castillejo et al., 2020) could underline the M1B Ant, PgGs, PgRs, AfPgGs, ANTHc, and COLOUR QTLs (Supplementary Table 5). In the male linkage map, the closest marker to the *MYB10-2* homoeo-allele at position 15,517,937 bp on the subgenome Fvb1-2 of the Camarosa reference genome is AX-89846847 at 118.78 cM on M1B. This marker is in the Bayesian credible interval of the flavan-3-ols AfCat_2011 mQTL (Supplementary Figure 3) but not in the other M1B QTLs intervals detected in our study.

### High Resolution Mapping of Major Fruit Colour Quantitative Trait Loci on Linkage Group 3A Allows the Detection of Potential Candidate Genes Associated With Intense Red Fruit Colour

**FIGURE 2:**
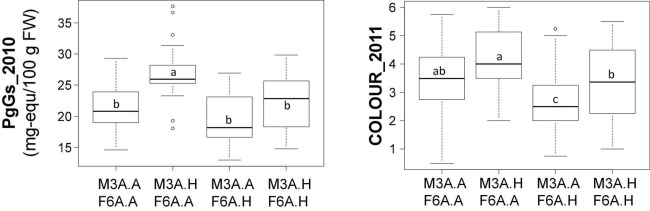
Contribution of the M3A and F6A quantitative trait loci (QTLs) to colour-related trait values. Effect of M3A and F6A alleles on PgGs content and COLOUR values. The allelic status (presence, H; absence, A) of the two markers, AX.89826440.M3A (M3A) and AX.89842368.F6A (F6A) is indicated on the abscissa. These markers were chosen because they were localised at the peak of colour-related QTLs (PgGs_2010 and COLOUR_2011) on M3A and F6A. Boxes represent the trait variation of individuals with the reported combination of alleles. Boxplots with the same letter are not significantly different (Kruskal–Wallis test, *P* < 0.05).

The two colour-related QTLs showing the highest percentage of variance (more than 20%), which are located on M3A and F6A (Ant_2010, PgGs_2010, and COLOUR_2011) (Table 2), display opposite effects on ANTHc (Supplementary Table 5). We investigated if they had epistatic relationships through the analysis of the allelic interaction by ANOVA (Figure 2). Variance analysis confirmed that the male M3A QTLs had indeed a positive effect on trait values and the female F6A QTLs a negative effect. However, because trait values were similar in individuals harbouring both M3A and F6A QTLs, we can conclude that there is only additive effect and likely no significant interaction between them.

**FIGURE 3:**
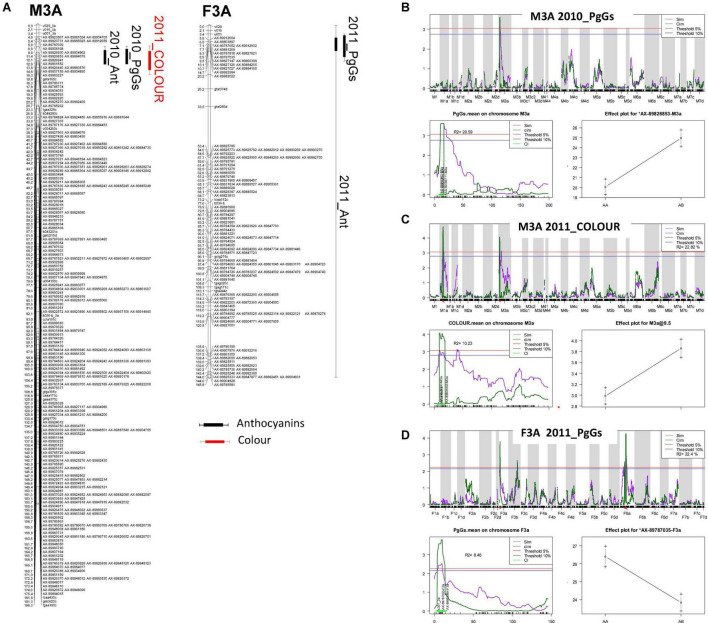
Localisation of colour-related QTLs on the linkage groups M3A and F3A. **(A)** Mapchart of linkage group LG3A. Linkage groups are represented in MapChart 2.3 (Voorrips, 2002) with a space of 3 mm per cM. Each boxplot corresponds to a QTL identified with a threshold of 10%. Bayesian credible interval of QTL is indicated at 5%. Ant, total anthocyanins; PgGs, pelargonidin-3-glucoside; COLOUR, visual evaluation of fruit colour. Each QTL name is preceded by the year (2010_ or 2011_). mQTL scans and effect of markers linked to **(B)** 2010_PgGs and **(C)** 2011_COLOUR QTLs localised on linkage group M3A (male) and **(D)** 2011_PgGs QTL on linkage group F3A (female). For each of the three QTLs, genome scan (top), scan on specific linkage groups M3A or F3A (bottom left) and plot effect of the QTL marker (bottom right) are shown. For QTL genome scan and scan of specific linkage group, LOD values are shown on the *y*-axis and genetic positions in centiMorgans are on the *x*-axis. Simple and composite interval mapping analysis (SIM and CIM) are represented by the purple solid and the dark green solid lines, respectively. Threshold of QTL detection at 5% (red) and 10% (blue) for each trait is represented. In scan on specific linkage group, Bayesian credible interval of QTL is indicated at 5% in light green. For each QTL flanking markers, markers of QTL and variance (*R*^2^) are indicated. For each trait, effect of QTL is indicated at the marker corresponding to the maximal LOD value of QTL.

We also identified overlapping Bayesian credible intervals for various QTLs, which would indicate the presence of pleiotropic or closely linked QTLs (Table 2 and Supplementary Figure 3). In particular, overlapping QTLS were found for total anthocyanins (Ant), PgGs and COLOUR on M3A and F3A (Figure 3A and Supplementary Table 5). We could detect mQTLs for Ant and PgGs on the male map (M3A) in 2010 and on the female map (F3A) in 2011, and a COLOUR QTL on M3A in 2011 (Figure 3A). CIM analysis further showed that the fruit COLOUR QTL and Ant and PgGs mQTls, which displayed high LOD scores values >3.0, were co-located in a narrow chromosomal interval (Figures 3B–D). Furthermore, the male allele from “CF1116” (on M3A) had a positive effect on the levels of anthocyanins and COLOUR (Figures 3B,C) while the female allele from “Capitola” (on F3A) had the opposite effect (Figure 3D). This points to the likely presence in this LG3A region of two different allelic variants, one from the male parent and one from the female parent, which affect either positively (the male homoeo-allelic variant) or negatively (the female homoeo-allelic variant) the colour-related traits. For Ant and PgGs, the percentages of phenotypic variance explained by the M3A mQTLs (*R*^2^ = ∼20) were more than twice as high as that of the F3A mQTLs (*R*^2^ = ∼8.5) (Supplementary Table 5).

**FIGURE 4:**
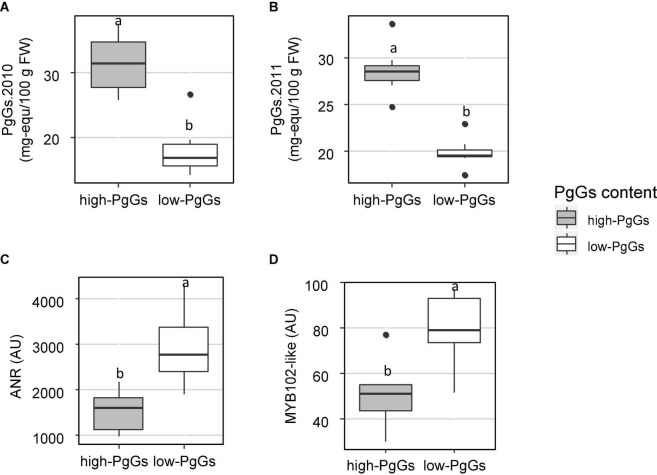
Expression of *ANR* and *MYB102-like ODORANT* in two extreme pools of individuals with contrasted pelargonidin-3-glucoside (PgGs) content. Two pools of seven individuals from the “Capitola” and “CF1116” progeny were constituted according to their PgGs content in **(A)** 2010 and **(B)** 2011. Microarray signal values (arbitrary units) are reported for **(C)** *ANR (FvH4_3g02980)* and **(D)** *MYB102-like ODORANT (FvH4_3g03780*). Boxplots with different letters are significantly different (Kruskal–Wallis test, *P* < 0.05).

To detect candidate genes underlying colour QTLs and their homoeo-allelic variants, we took advantage of the strong synteny between *F. vesca* genome (Edger et al., 2018) and the subgenome of *F. vesca* Fvb3-4, which corresponds to our LG LG3A (Hardigan et al., 2021a). For both QTLs, we identified on the diploid FvH4 *F. vesca* genome an interval framed by SNP markers with physical positions overlapping the Bayesian credible intervals of the M3A and F3A QTLs. On M3A, this region is flanked by AX-89904962 and AX-89785774 Affymetrix markers and spans an interval on chromosome 3 (Fvb3) from 1,213,489 to 2,673,762 b, while on F3A this region is larger (826,085–2,539,615 b) and almost overlapped with the M3A interval. Based on the latest annotation of the *F. vesca* genome (Li et al., 2019), we identified a total of 392 genes in the M3A/F3A interval (826,085–2,673,762 b) (Supplementary Table 7) and searched them for candidate genes possibly involved in the regulation and/or synthesis of flavonoids in strawberry fruit. Among them, the most likely candidate gene is annotated as NAD(P)-binding Rossmann-fold superfamily protein (*FvH4_3g02980*). It encodes an ANR enzyme, which catalyses the conversion of pelargonidin to epiafzelechin and of cyanidin to epicatechin (Figure 1A) and has previously been demonstrated to control anthocyanin accumulation in *F. vesca* fruit (Fischer et al., 2014). Two additional candidate genes encoding MYB-related transcription factors annotated as *MYB58* (*FvH4_3g03680*) and *MYB102-like ODORANT* (*FvH4_3g03780*) are found in the M3A/F3A interval. To further investigate the candidate genes, we first mined transcriptome data obtained by microarray analysis of 21 individuals from the segregating population which displayed contrasted flavonoid-related phenotypes. To this end, we first constituted two groups of seven F1 individuals each displaying either high or low PgGs content at the red ripe stage, with a PgGs content ratio between the two pools of 1.7 in 2010 and 1.4 in 2011 (Figure 4 and Supplementary Table 8). We next analysed these individuals for differentially-expressed genes (DEGs) using a custom-made oligonucleotide-based (60-mer length) platform representing a total of 18,152 strawberry unigenes (Ring et al., 2013). To identify significant DEGs present in the region of interest, we applied a Student’s *t*-test on the two phenotypic groups for all genes located in the Bayesian credible interval. Out of the 392 genes found in the M3A/F3A interval that harbours the QTL and mQTLs of interest, 304 genes including *ANR* (*FvH4_3g02980*), *MYB102-like ODORANT* (*FvH4_3g03780*) and *MYB58* (*FvH4_3g03680*) were present on the microarray (Supplementary Table 9). Among them, 50 DEGs (*P*-value < 0.05) were found, which included *ANR* and *MYB102-like ODORANT* but not *MYB58*. The expression of the *ANR* and *MYB102-like ODORANT* genes were respectively 1.6 and 1.9-fold higher in the individuals with low PgGs content than in the individuals with high PgGs content (Figure 4 and Supplementary Table 9). Analysis of the remaining DEGs did not highlight any additional candidate gene for the control of anthocyanin accumulation in the fruit (Supplementary Table 9).

**FIGURE 5:**
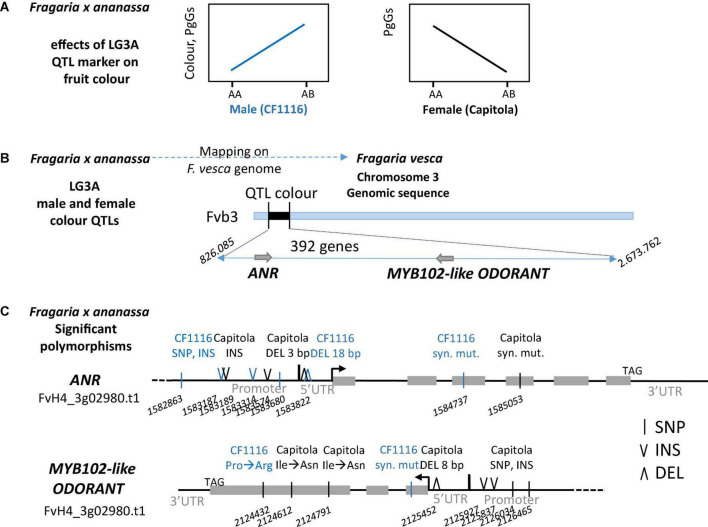
Polymorphisms identified in the *MYB102-like ODORANT* and *ANR* genes. Schematic representations of **(A)** effects of LG3A QTL markers: positive effect of the male marker and negative effect of the female marker on fruit colour and **(B)** the position of the M3A/F3A QTL interval on chromosome Fvb3. **(C)** Polymorphisms found in the two candidate genes *ANR* (*FvH4_3g02980*) and *MYB102-like ODORANT* (*FvH4_3g03780*). Only polymorphisms between “Capitola” (in black) and “CF1116” (in blue) that respond to a Chi-squared test for the presence of 1 allele out of the 8 alleles (1:7 ratio) are reported. DEL, deletion; INS, insertion; SNP, single nucleotide polymorphism; syn. mut., synonymous mutation. Three letter code is used for amino acids. Grey box represents exon. Numbers refer to positions on Fvb3 (*Fragaria vesca* Whole Genome v4.0.a1 Assembly).

We then performed the whole genome sequencing (Illumina Hiseq 3000) of the “Capitola” and “CF1116” parents of the segregating population. We obtained 137 and 142 million 150 pb paired-end reads, representing 50.7X and 52.7X coverage of the octoploid genome (Edger et al., 2019), respectively. As summarised in Figure 5A, the LG3A colour QTL marker has a positive effect on fruit colour in the male (“CF1116”) and a negative effect in the female (“Capitola”). Thanks to the alignment of paired reads “Capitola” and “CF1116” to the FvH4 *F. vesca* genome (Edger et al., 2018), which is syntenic to the subgenome Fvb3-4 of the Camarosa octoploid reference genome (Hardigan et al., 2021a), we had access to sequence polymorphisms of *ANR* and *MYB102-like ODORANT* genes in the four sub-genomes of both parents (Figure 5B). Because *ANR* and *MYB102-like ODORANT* are up-regulated in progeny individuals with low PgGs content (Figure 4), we first analysed sequence polymorphisms in regions that may affect gene regulation and that were different between both parents. In the 1 kb region upstream of the transcription start site of both genes, we found several SNPs and insertions by comparison of the “Capitola” and “CF1116” sequences. In the 5′untranslated region (UTR) of *ANR*, we found two deletions, one of 3 bp in Capitola and one of 18 pb in CF1116. In the 5′UTR of *MYB102-like ODORANT*, we found only one deletion of 8 bp in “Capitola.” We next analysed the protein coding regions for polymorphisms that are different between both parents and detected two SNPs in *ANR* and three SNPs in *MYB102-like ODORANT*.

To further reduce the number of candidate polymorphisms, we subsequently considered only those polymorphisms fitting the 1:7 ratio expected for single male or female homoeo-alleles (Chi-squared test; *P* > 0.05) located on a single LG (Figure 5C). Interestingly, the 18 bp deletion located from −109 to −127 bp upstream of the start codon in the 5′UTR of the *ANR* gene of “CF1116” (the male parent) (Figure 5C) was present in 13% of the reads. Analysis of the corresponding CTTCTTCCTCTTCTTCTT sequence with PlantRegMap (Jin et al., 2017) predicted that the deletion was in the 21 nucleotides binding site of a MADS Agamous-like transcription factor (*Arabidopsis AT2G45660*). Blast analysis of *F. vesca* v1.0 ab hybrid reference genome sequence at GDR (Jung et al., 2019) using AT2G45660 protein sequence as a query and visualisation of gene expression of top hits using *F. vesca* eFP browser (Hawkins et al., 2017) allowed the identification of four strawberry homologs expressed in fruit cortex along development (*gene24852*, *gene04229*, *gene26119*, and *gene06301*). Other small insertion/deletions (INDELS) or SNPs found in promoter or 5′UTR of *ANR* and *MYB102-like ODORANT* genes did not match known motifs. Two SNPs found in the protein coding region of *ANR* are synonymous and do not affect the function of the protein. One SNP found in the protein coding region of *MYB102-like ODORANT* is synonymous while two SNPs found in exon 3 of the MYB102-like ODORANT of “Capitola” (the female parent) are non-synonymous mutations leading to H146Q and I206N amino acid substitutions (Figure 5C).

### Development of a Predictive Marker for Improvement of Red Fruit Colour Intensity Based on the Anthocyanidin Reductase Homoeo-Allele Carrying a 18 bp 5′UTR Deletion

**FIGURE 6:**
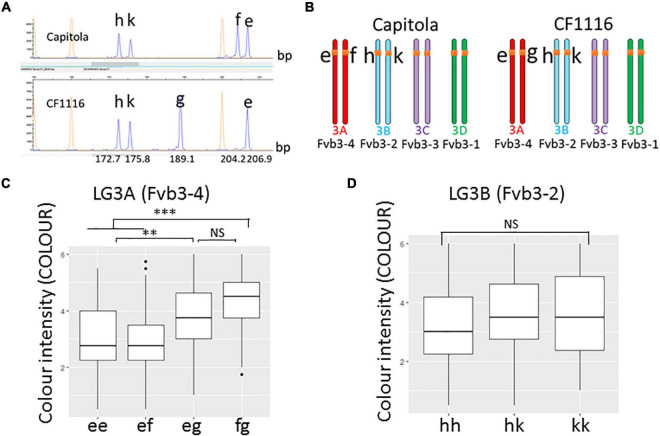
Effect of homoeo-alleles of the BxANR_5UTR marker on fruit colour. **(A)** Analysis by capillary electrophoresis (ABI 3730) of a PCR-amplified region framing the 18 bp 5′UTR deletion found in *ANR* led to the identification of five peaks (in blue) corresponding to five homoeo-alleles (h, k, g, f, and e). On LG3A (Fvb3-4), the f allele is only found in “Capitola,” the g allele only in “CF1116” while the e, h, and k homoeo-alleles are found in both “Capitola” and “CF1116.” Estimated fragment sizes of the ANR 5′UTR sequences amplified with M13-tailed primers are indicated on the abscissa. **(B)** Mapping of *ANR* homoeo-alleles on homoelogous group 3. **(C)** Effect of homoeo-alleles e, f, and g on colour intensity. The allelic status of individuals is indicated on the abscissa. **(D)** Effect of homoeo-alleles h and k on colour intensity. The allelic status of individual is indicated on the abscissa. Colour intensity was evaluated on a scale from 0 (very pale red-orange) to 6 (very dark red). Boxes represent the trait variation of individuals with the reported combination of alleles. Kruskal–Wallis test: ^**^P < 0.01; ^***^P < 0.001; NS, not significant.

To develop genetic markers for improvement of strawberry fruit colour by MAS, we first analysed the allelic status of SNP marker AX-89826853 (position: 1,981,447 bp on FvH4, i.e., close to *ANR*) using the 14 selected progeny individuals with contrasted PgGs content. All individuals with high PgGs showed the presence of this SNP marker while individuals with low PgGs did not. To further check whether the 5′UTR deletion found in *ANR* could be used for developing a genetic marker tightly linked to the M3A colour QTLs, we next PCR-amplified a 182 bp region (size in the diploid reference genome) spanning the 18 bp deletion found in “CF1116” and analysed the resulting PCR product by CE (Figure 6A) in the segregating population derived from “Capitola” and “CF1116.” Beforehand the PCR product (hereafter named BxANR_5UTR) was cloned in a plasmid vector and sequenced to confirm that the expected 5′UTR sequence of *ANR* was amplified. We identified five CE peaks in “Capitola” and “CF1116” that correspond to five homoeo-alleles; three of them are common to both parents (h, k, and e), one is specific to “Capitola” (f) and one is specific to “CF1116” (g) (Figure 6B). Noteworthy, the estimated size of the peak for allele g, which was observed only in the male parent “CF1116,” corresponds approximately to the size of the deleted variant of BxANR_5UTR while the size of the allele f would correspond to the 3 bp deleted variant specific to “Capitola” (Figure 6A). The h and k homoeo-alleles found in both “Capitola” and “CF1116” have smaller peak sizes corresponding to PCR fragments carrying several deletions.

Within the five homoeo-alleles, three homoeo-alleles (e, f, and g) and two homoeo-alleles (h and k) are in coupling/repulsion configurations (ef × eg and hk × hk). Using JoinMap, we further mapped the homoeo-alleles to LG3A (Fvb3-4; alleles e, f, and g) and LG3B (Fvb3-2; alleles h and k) (Figure 6B). Surprisingly, BLAST search of the “Camarosa” reference genome sequence (Jung et al., 2019) found only one *ANR* gene, which is located on Fvb3-2, and failed to identify *ANR* on Fvb3-4, which is the subgenome harbouring the M3A colour QTL. Thanks to the position of Affymetrix markers close to the e, f, g, h, and k homoeo-alleles, we could nevertheless confirm that the positions of the e, f, and g homoeo-alleles are orthologous to those of the h and k homoeo-alleles, thus indicating that the *ANR* gene is indeed present in the Fvb3-4 subgenome of “Capitola” and “CF1116.” Possible explanations are that *ANR* has been deleted from Fvb3-4 in the “Camarosa” variety or that the sequence of the Fvb3-4 region is incomplete in the reference genome. We favour the last hypothesis because this gene is present in the chr_3A (corresponding to Fvb3_4) of the recently released genome sequence of Royal Royce (Hardigan et al., 2021b).

We next analysed the effect of the various combinations of the five homoeo-alleles on fruit colour intensity in the segregating population derived from “Capitola” and “CF1116” (137 progeny individuals and the two parents) (Figures 6C,D). Remarkably, the g homoeo-allele, which corresponds to the deleted variant of BxANR_5UTR that is co-localised on M3A (Fvb3-4) with the AX-89826853 marker linked to the PgGs, Anth and COLOUR QTLs (Figures 3A,B, 6B), had a significant and positive effect on fruit colour as expected (Figure 5A). When present in the genetic combination, the g homoeo-allele resulted in darker red fruit with an average increase in the COLOUR score from 3 to 3.9 (on a 0 to 6 scale; Supplementary Figure 1), which is considerable (Figures 6C,D). Because the analysis of BxANR_5UTR is straightforward and allows the identification of strawberry genotypes with dark red fruit, it can therefore be used in MAS as a genetic marker for breeding strawberry varieties with more intense red fruit colour.

## Discussion

The biosynthesis of flavonoids, and that of anthocyanins in particular, has been thoroughly investigated in strawberry because of their considerable importance to the sensorial and nutritional quality of the fruit (Mezzetti et al., 2018). As evidenced by reverse genetic studies, the MYB, bHLH, and WD-repeat proteins transcription factors play prominent roles in anthocyanin regulation in strawberry (Jaakola, 2013; Salvatierra et al., 2013; Schaart et al., 2013; Medina-Puche et al., 2014; Härtl et al., 2017; Wang et al., 2020; Zhang et al., 2020). Additional candidates are the structural enzymes involved in flavonoid and anthocyanin pathways (Almeida et al., 2007; Griesser et al., 2008; Fischer et al., 2014) and in connected pathways leading to phenylpropanoid-derived compounds (Ring et al., 2013). Recent studies aimed at deciphering the architecture of flavonoids in strawberry allowed the localisation of tens of flavonoid mQTLs and, for some of them, the identification of underlying candidate genes (Davik et al., 2020; Labadie et al., 2020; Pott et al., 2020). In addition, genetic variations in MYB10, a master regulator of anthocyanin biosynthesis, were shown to be responsible for white fruit phenotype in the wild diploid strawberry *F*ragaria *nilgerrensis* (Zhang et al., 2020) and underlying variations of fruit skin and flesh colour in the diploid woodland strawberry *F. vesca* and the octoploid *F. × ananassa* (Castillejo et al., 2020; Wang et al., 2020). Regardless of these advances, the molecular factors involved in variations of strawberry fruit colour remain largely unknown (Whitaker et al., 2020).

Here, we discovered likely candidate genes and homoeo-allelic variations underlying several colour-related QTLs through the (i) high resolution mapping of colour traits broken down into individual components, (ii) identification of specific regions carrying the QTLs in the donor subgenomes, and (iii) combination of whole genome sequencing of the parents and transcriptome analysis of selected progeny individuals. Furthermore, by focusing on the major colour-related QTLs found on M3A LG and on the underlying *ANR* candidate gene, we could design the BxANR_5UTR marker which is of considerable help to improve red fruit colour intensity in strawberry.

### Anthocyanin Variations Are Largely Controlled by the *Fragaria vesca*–Derived Subgenome in the Population Studied

In polyploid plant species, each trait is likely controlled by homoeologous gene series or homoeo-alleles. In the octoploid strawberry, homoeo-alleles are located at orthologous positions on one of the four subgenomes (Edger et al., 2019). Remarkably, our results showed that, within a given HG, mQTLs for flavonoids and anthocyanins were mostly located on a single LG (i.e., on a single subgenome). As an example, the A subgenome was likely responsible for the major PgGs mQTLs localised on M3A, F3A, and F6A LGs. Furthermore, the main LGs accounting for half of male colour-related QTLs (three LGs) and for the majority of female QTLs (three LGs) can be attributed to the A subgenome, which is mainly derived from *F. vesca* (Hardigan et al., 2021a), thus highlighting the predominant role of *F. vesca* in fruit flavonoid metabolism and quality. Our findings are thus consistent with transcriptome analyses showing that *F. vesca* homoeologs are responsible for almost 89% of the anthocyanin biosynthesis in octoploid strawberry (Edger et al., 2019).

The contribution of the various diploid genomes to octoploid genome is complex (Edger et al., 2019; Liston et al., 2020, Hardigan et al., 2021a). We followed Hardigan et al. (2021a) for the assignment of a subgenome to a given LG. In the population studied, subgenomes other than *F. vesca* may play key roles in flavonoid metabolism, including, e.g., subgenomes derived from *F. iinumae* and *F. viridis* for PgGs; subgenomes derived from *F. iinumae, F. viridis*, and *F. nipponica* for PgRs; subgenomes derived from *F. iinumae-* and *F. viridis* for COLOUR. The crucial role in the regulation of strawberry fruit colour of the *MYB10*-2 homoeo-allele localised on LG1B (*F. iinumae*–derived subgenome) has been demonstrated recently (Castillejo et al., 2020). However, in our population, this homoeo-allele was not included in the Bayesian credible interval of the colour-related QTLs detected on M1B (Supplementary Figure 3) and is likely not responsible for the colour variations observed.

### The *Anthocyanidin Reductase* Gene of the *Fragaria vesca*–Derived Subgenome Is a Likely Candidate to Control Major Variations in Strawberry Fruit Colour

In *F. × ananassa*, pinpointing with high resolution the position of flavonoid mQTLs and other colour-associated QTLs opens the possibility to identify the homoeo-alleles responsible for major variations in colour-related traits. Major QTLs detected on LG3A for the total anthocyanins, the PgGs and the visually scored fruit colour are all co-located at the beginning of LG3A (Figure 3A) and overlap with the colour-related QTLs previously detected over 3 years of study for the physical parameters L and b (colour space values) (Lerceteau-Köhler et al., 2012), indicating that these colour QTLs are robust. We could further map with high resolution the genetic architecture of the colour trait and show that: (i) PgGs mQTLs displaying high LOD scores values >3.0 are co-localised on the male (M3A) and female (F3A) maps in a narrow chromosomal interval encompassing 392 genes, (ii) the male homoeo-allele from “CF1116” has a positive effect on PgGs content (Figures 3B,C) while the female homoeo-allele from “Capitola” has the opposite effect (Figure 3D), and (iii) homoeo-allelic variants of two candidate genes encoding ANR and MYB102-like ODORANT, both of which are differentially expressed in pools of individuals with contrasted PgGs contents, are respectively found in the male (“CF1116”) and female (“Capitola”) parents.

The possible candidate gene underlying the female F3A colour-related QTL (∼8.5% of explained variance) is a MYB transcription factor (TF). MYB TFs play predominant roles in the control of phenylpropanoid pathway and anthocyanin biosynthesis in strawberry (Salvatierra et al., 2013; Schaart et al., 2013; Medina-Puche et al., 2014; Wang et al., 2020). However, the most likely MYB candidate gene underlying the female F3A PgGs mQTL is not homologous to a well characterised strawberry MYB such as MYB10 (Medina-Puche et al., 2014; Hawkins et al., 2016; Castillejo et al., 2020; Wang et al., 2020; Zhang et al., 2020). It encodes a MYB102-like ODORANT protein homologous to the petunia *R*2R3-MYB ODORANT1 (ODO1) which regulates floral-scent related genes in petunia (Spitzer-Rimon et al., 2010). Ectopic expression of *ODO1* in tomato activates phenylpropanoid metabolism without affecting volatiles (Dal Cin et al., 2011). In addition to the two SNPs leading to synonymous amino acid changes in *ODO1* coding region, the *MYB102-like ODORANT* gene from “Capitola” LG3A carries several SNPs and INDELS in the 5′UTR and promoter regions. This opens the possibility that the differential expression of this gene, which was observed in progeny individuals with contrasted PgGs content (Figure 4), could be responsible for variation in anthocyanin biosynthesis.

The major colour-associated QTLs (PgGS, Anth, and COLOUR; ∼20% of explained variance) are located on the male M3A LG. The most likely underlying candidate gene is *ANR*. In the pseudo F_1_ population studied, each individual is heterozygous at a given locus. Therefore, only one *ANR* homoeo-allele localised on the *F. vesca*–derived A subgenome underlies the male (“CF1116”) M3A colour-associated QTLs. Mapping the WGS of the “Capitola” and “CF1116” parents to the FvH4 *F. vesca* genome (Edger et al., 2018), which is syntenic to the subgenome Fvb3-4 (3A) (Hardigan et al., 2021a), allowed us to identify a large deletion (18 bp) in the 5′UTR of an *ANR* homoeo-allele (homoeo-allele g) carried by the male parent (“CF1116”). Combining genotyping by CE with visual scoring of fruit colour of the segregating population unequivocally associated intense red fruit colour to the presence of the *ANR* homoeo-allele g (Figure 6C).

These genetic indications, which support the contribution of ANR to the control of anthocyanin accumulation in strawberry, are in line with our previous molecular findings in *F. vesca* (Fischer et al., 2014). In the wild diploid *F. vesca*, ANR is encoded by a single gene localised on Fvb3. ANR converts the anthocyanidins pelargonidin and cyanidin to flavan-3-ols (epiafzelechin or epicatechin); concurrently, anthocyanidins can also be converted to anthocyanins (Figure 1A). In strawberry, flavan-3-ols accumulate in green fruit during the early stages of fruit development while the red-coloured anthocyanins accumulate after the onset of ripening (Fischer et al., 2014). Moreover, *ANR* is highly expressed in fruit cortex during early fruit development, up to the white fruit stage, and moderately so thereafter (Hawkins et al., 2017), suggesting a negative correlation between *ANR* transcript abundance and anthocyanin accumulation in the fruit. ANR may thus play a prominent role in the determination of fruit colour by controlling the trade-off between flavan-3-ols biosynthesis and anthocyanin biosynthesis. Indeed, we already demonstrated that silencing *ANR* in *F. vesca* fruit redirects phenypropanoid flux from flavan-3-ols to anthocyanins, resulting in early anthocyanin accumulation in the fruit (Fischer et al., 2014). Conversely, overexpression of tea (*Camellia sinensis*) *ANR* genes in tobacco results in a significant loss of flower red-pigmentation due to reduced ANTHc (Zhao et al., 2017). Altogether, these published results are consistent with our observation of a negative correlation between *ANR* transcript abundance and PgGs content in progeny individuals (Figure 4). They further support the hypothesis that the 18 bp *ANR* 5′UTR deletion found in the male homoeo-allele g may result in down-regulation of *ANR*, promotion of the flux from anthocyanidins to anthocyanins, accumulation of PgGS in the fruit and consequently more intense red colour in individuals of the progeny where homoeo-allele g is present.

The mechanism by which deletion in 5′UTR of *ANR* would achieve this effect remains to be elucidated. The 5′UTR plays a pivotal role in controlling gene expression through the regulation of transcript abundance or the alteration of mRNA translation efficiency or stability. Moreover, 5′UTR mutations can control transcript abundance by altering functional elements (Lim et al., 2021). The 5′UTR mutation in *ANR* homoeo-allele g deletes a *cis*-regulatory element which is a putative MADS box binding motif. By mining strawberry genomic databases, we found four MADS box genes that may possibly interact with the deleted motif and are expressed in fruit cortex along development (Hawkins et al., 2017). As the involvement of MADS-box in anthocyanin accumulation has been reported in different species including bilberry (Jaakola et al., 2010) and pear (Wang et al., 2017), which are also Rosaceae species, future work would focus on the possible involvement of the identified MADS box genes in the regulation of *ANR* and thus in the control of fruit colour in strawberry.

## Conclusion

In summary, with the precise assignment of QTLs to narrow genomic regions, the genetic architecture of fruit colour can now be explored at an unprecedented level in *F. × ananassa*. From a more applied perspective, the present study further shows that high resolution mapping combined with whole-genome sequencing can help discover genetic variants associated with colour traits, from which simple and breeder-friendly genetic markers such as BxANR_5UTR can be designed for accelerating the selection process.

## Accession Numbers

Anthocyanidin reductase: FvH4_3g02980 (gene24665); MYB102-like ODORANT: FvH4_3g03780 (gene30725); MYB58: FvH4_3g03680 (gene30736).

## Data Availability Statement

Original datasets are available in a publicly accessible repository: The original contributions presented in the study are publicly available. This data can be found here: https://doi.org/10.15454/FMCVMA.

## Author Contributions

BD conceived and designed the experiments. AuP conducted hands-on experiments and data collection. AuP, AlP, and AG participated in the data collection. JM-B and JC designed the microarray. LR, TH, and WS generated LC-LS data. AlP designed the genetic marker and performed the genetic segregation analyses. ML, GV, CR, and BD conducted the data analysis and performed the statistical analysis. CR wrote the original draft. All authors read and approved the final manuscript.

## Conflict of Interest

The authors declare that the research was conducted in the absence of any commercial or financial relationships that could be construed as a potential conflict of interest.

## Publisher’s Note

All claims expressed in this article are solely those of the authors and do not necessarily represent those of their affiliated organizations, or those of the publisher, the editors and the reviewers. Any product that may be evaluated in this article, or claim that may be made by its manufacturer, is not guaranteed or endorsed by the publisher.

## Footnotes

1
http://www.ctifl.fr/Pages/Kiosque/DetailsOuvrage.aspx?IdType=3&idouvrage=833

2
https://www.ctifl.fr/pages/Kiosque/ListeOuvrage.aspx?idType=3
