# Supplementary material for: High Resolution Quantitative Trait Locus Mapping and Whole Genome Sequencing Enable the Design of an Anthocyanidin Reductase-Specific Homoeo-Allelic Marker for Fruit Colour Improvement in Octoploid Strawberry (Fragaria × ananassa)
Source: Front Plant Sci. 2022 Mar 18;13:869655. doi: 10.3389/fpls.2022.869655 (PMC8972132; doi:10.3389/fpls.2022.869655)
Supplement: Supplementary file 1 [file Data_Sheet_1.PDF]

Supplementary Material

High resolution QTL mapping and whole genome sequencing enable design of an ANR homoeoallele-specific marker for fruit colour improvement in octoploid strawberry (F. x ananassa)

Marc Labadie, Guillaume Vallin, Aline Potier, Aurélie Petit, Ludwig Ring, Thomas Hoffmann, Amèlia Gaston, Juan Munoz-Blanco, José L. Caballero, Wilfried Schwab, Christophe Rothan, Béatrice Denoyes

Supplementary datasets, figures and tables.

Supplementary Data Files

Supplementary_Dataset1: read_Cap_ANR_Fvb3_1582300-1587019.fasta
reads of Capitola around ANR (extraction of reads in reference genome of FvH4-v4.a1 Fvb3:1582300-1587019)

Supplementary_Dataset2: read_CF_ANR_Fvb3_1582300-1587019.fasta
reads of CF1116 around ANR (extraction of reads in reference genome of FvH4-v4.a1 Fvb3:1582300-1587019)

Supplementary_Dataset3: read_Cap_Myb102_Fvb3_2123741-2127200.fasta
reads of Capitola around MYB102-like ODORANT (extraction of reads in reference genome of FvH4-v4.a1 Fvb3:2123741-2127200)

Supplementary_Dataset4: read_CF_Myb102_Fvb3_2123741-2127200.fasta
reads of CF1116 around MYB102-like ODORANT (extraction of reads in reference genome of FvH4-v4.a1 Fvb3:2123741-2127200)

All supplementary data files are available in the INRAE dataverse DOI https://doi.org/10.15454/FMCVMA

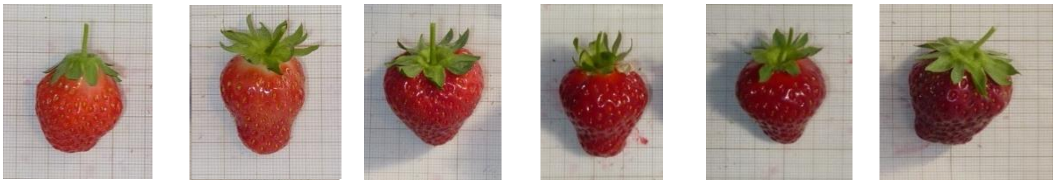

Individual	25	194	12	207	211	13
Mean score	1.0	2.0	3.0	4.0	5.0	6,0

Supplemental Fig 1. Colour score of ripe fruits.
Visual evaluation of colour on a scale from 1 (very pale red) to 6 (very dark red) on ripe fruits harvested in 2011. Six individuals from the progeny issued from the cross between ‘Capitola’ and ‘CF1116’ (CxC) are shown.

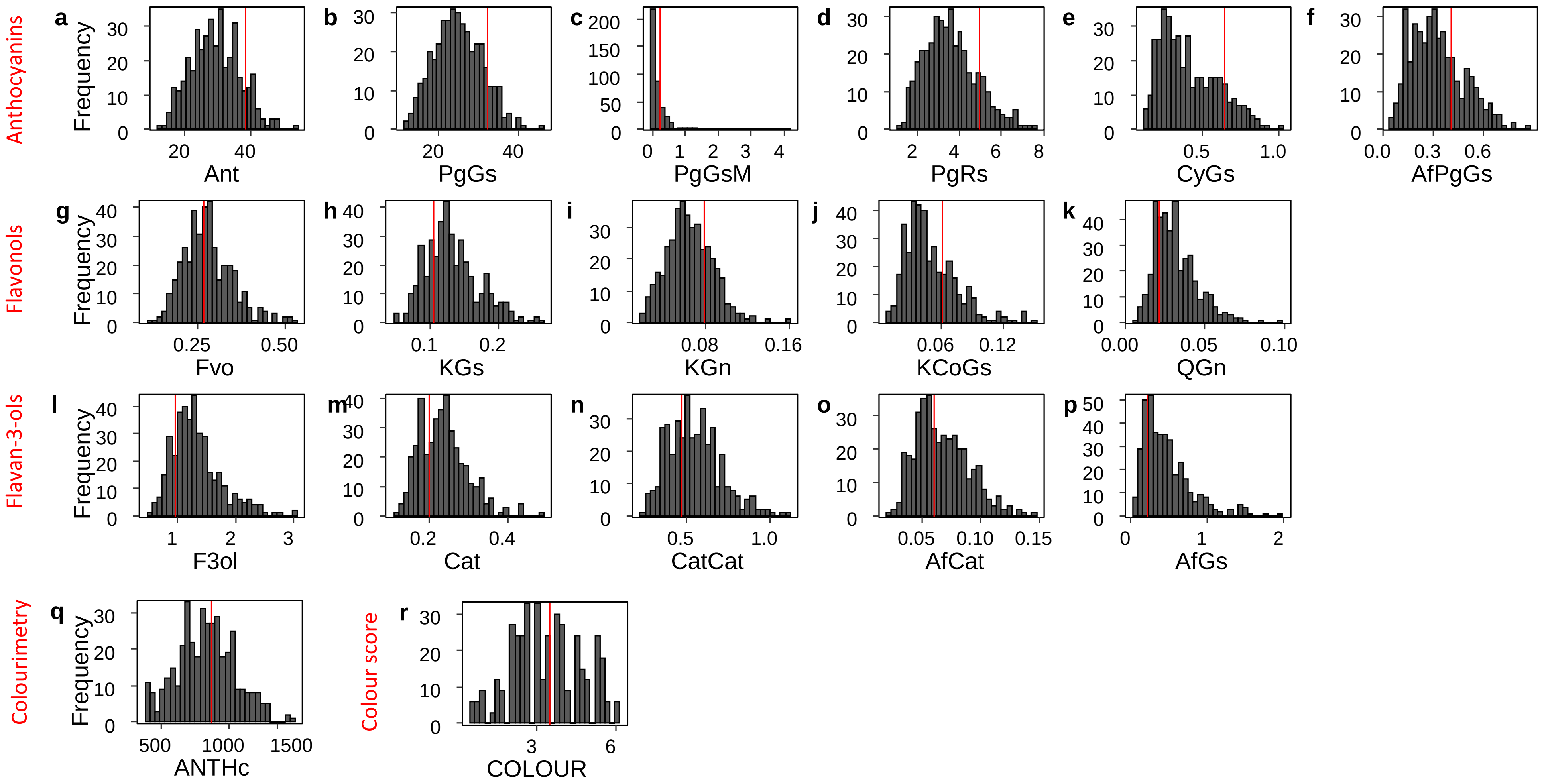

Supplemental Figure 2. Distribution in 2011 of the progeny mean for flavonoid metabolites, total anthocyanins assessed by colourimetry and colour assessed visually.

Mean phenotypic values from parents are represented in red for 'Capitola'. Ant, Fvo, F3ol values were obtained by summation of total anthocyanins, total flavonols and total flavan-3-ols, respectively; PgGs, Pelargonidin-3-glucoside; PgGsM, Pelargonidin-3-glucoside-malonate; PgRs, Pelargonidin-3-rutinoside; CyGs, Cyanidin-3-glucoside; AfPgGs, (epi)Afzelechin-pelargonidin-3-glucoside; KGs, Kaempferol-glucoside; KGn, Kaempferol-glucuronide; KCoGs, Kaempferol-coumaryl-glucoside; QGn, Quercetin-glucuronide; Cat, Catechin; CatCat, (epi)Catechin dimers; AfCat, (epi)Afzelechin-(epi)catechin dimers; AfGs, (epi)Afzelechin-glucoside; ANTHc, anthocyanins (colourimetry). The flavonoid metabolites values are expressed as mg-equ/100 g FW assuming a response factor of 1. ANTHc results are expressed as mg pelargonidin-3-glucoside equivalents/100 g FW. COLOUR values were assessed on a 0 to 6 scale. Values are the means of n = 3 replicates per genotype, except for COLOUR (n = 2).

A. QTLs on male linkage map

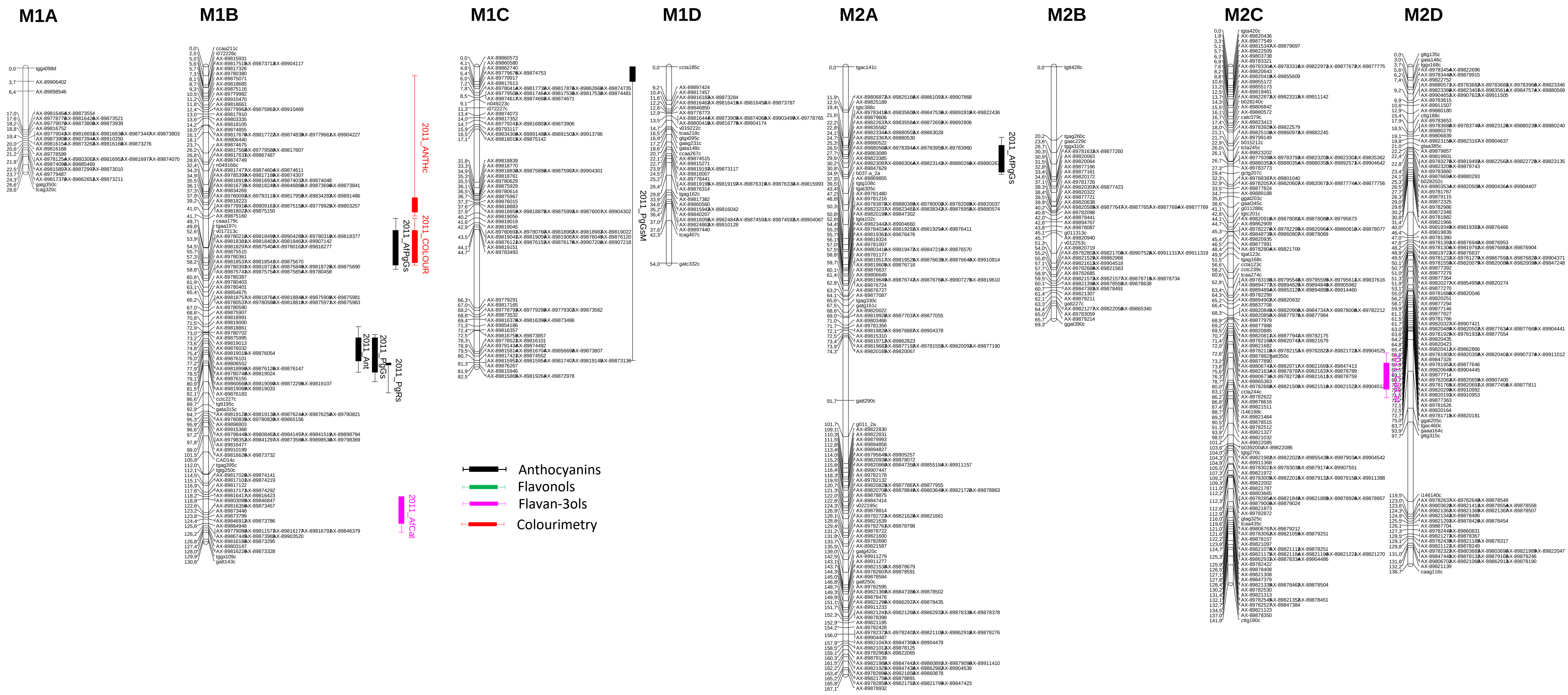

Supplemental Figure 3. Localization of all detected QTLs on male (A) and female (B) linkage maps for flavonoid metabolites, total anthocyanins assessed by colourimetry and colour assessed visually.

The QTLs for the various flavonoid metabolites, for the total anthocyanins measured by colourimetric assays or for colour assessed visually have a colour code that is shown on the bottom left of the figure. The QTL name is suffixed according to the year and the trait. The linkage groups are represented in MapChart 2.3 (R. E. Voorrips, 2002) with a space of 3mm by cM. Each boxplot corresponds to a QTL identified with a threshold of 10%. Bayesian credible interval of QTL is indicated at 5%. Ant, total anthocyanins; PgGs, Pelargonidin-3-glucoside; PgGsM, Pelargonidin-3-glucoside-malonate; PgRs, Pelargonidin-3-rutinoside; CyGs, Cyanidin-3-glucoside; AfPgGs, (epi)Afzelechin-pelargonidin-3-glucoside; Fvo, total flavonols; KGs, Kaempferol-glucoside; KGn, Kaempferol-glucuronide; KCoGs, Kaempferol-coumaryl-glucoside; QGn, Quercetin-glucuronide; F3ol, total flavan-3-ols; Cat, Catechin; CatCat, (epi)Catechin dimers; AfCat, (epi)Afzelechin-(epi)catechin dimers; AfGs, (epi)Afzelechin-glucoside; ANTHc, anthocyanins (colourimetry); COLOUR, colour (assessed visually).

A. QTLs on male linkage map - continued

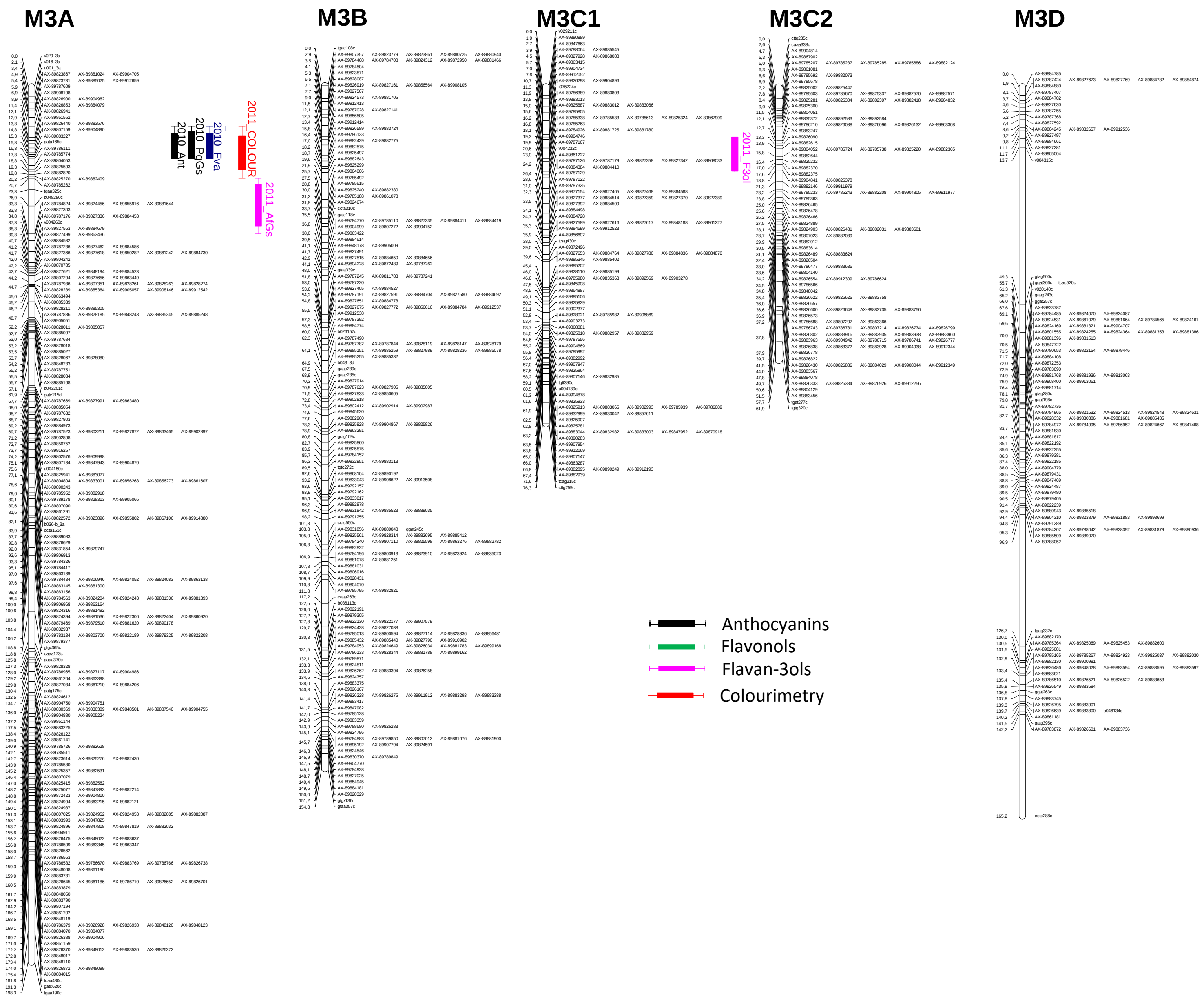

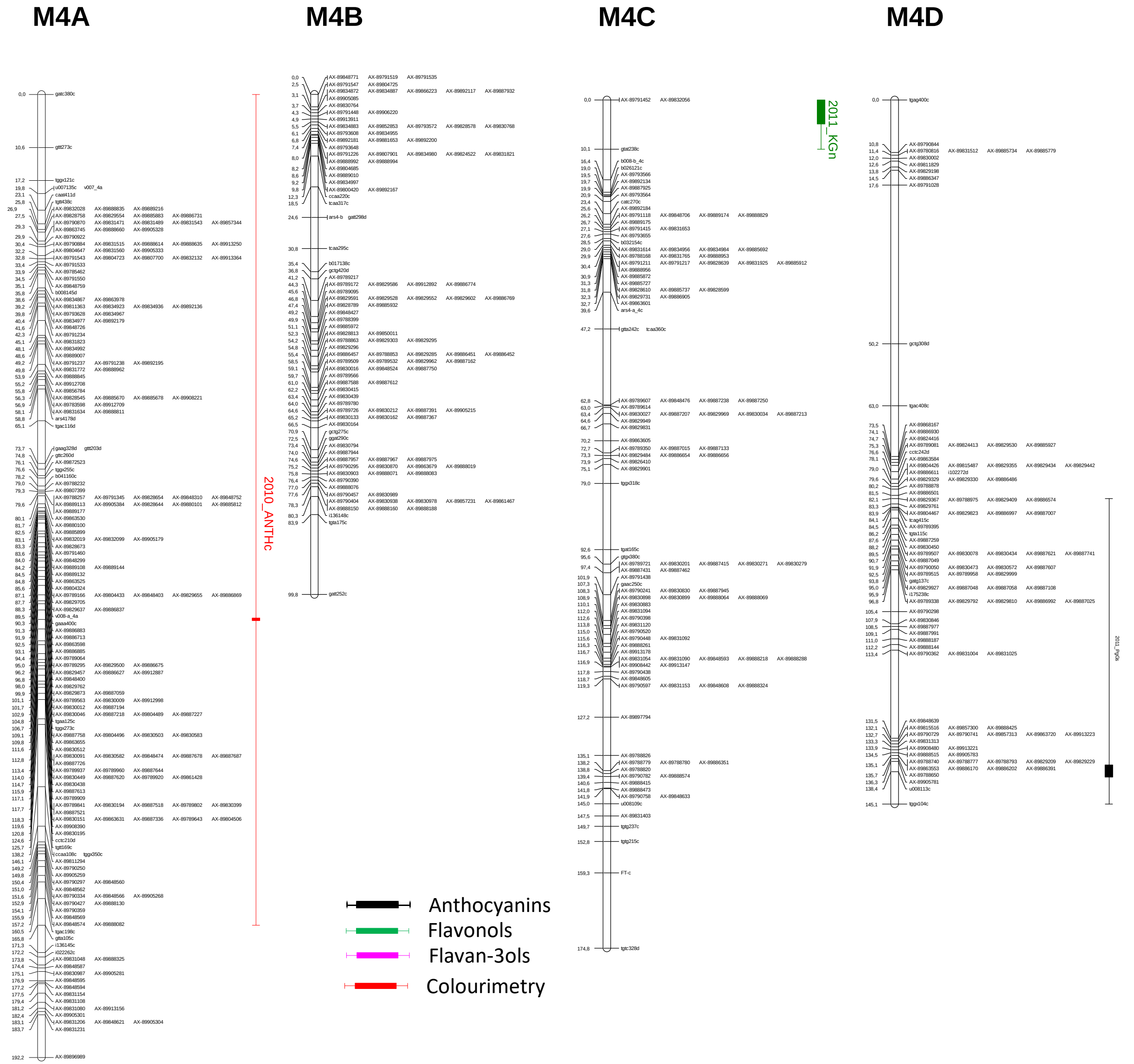

M4A
M4B
M4C
M4D
2010_ANTHc
2011_KGn
Anthocyanins
Flavonols
Flavan-3ols
Colourimetry

A. QTLs on male linkage map - continued

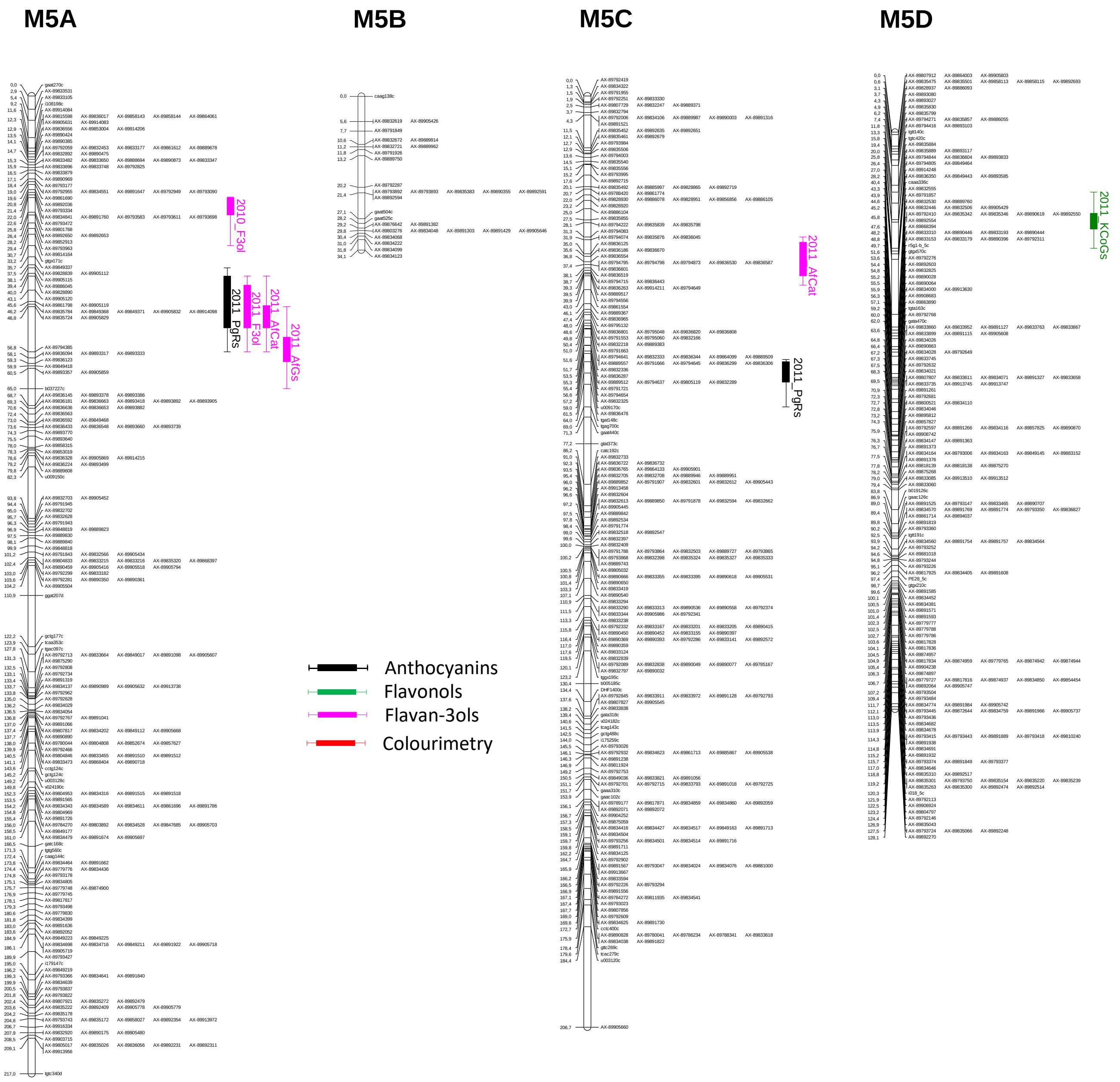

A. QTLs on male linkage map - continued

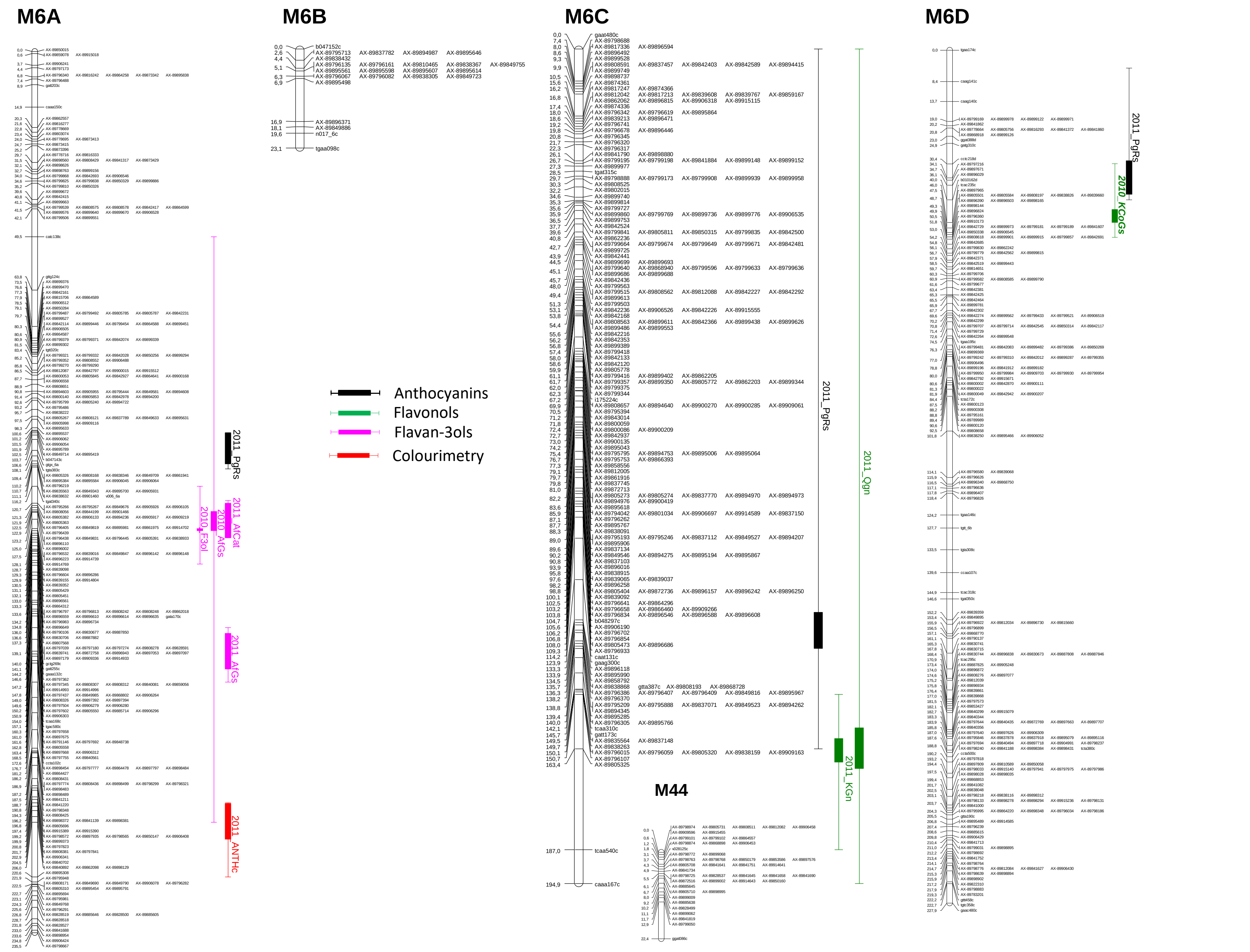

A. QTLs on male linkage map - continued

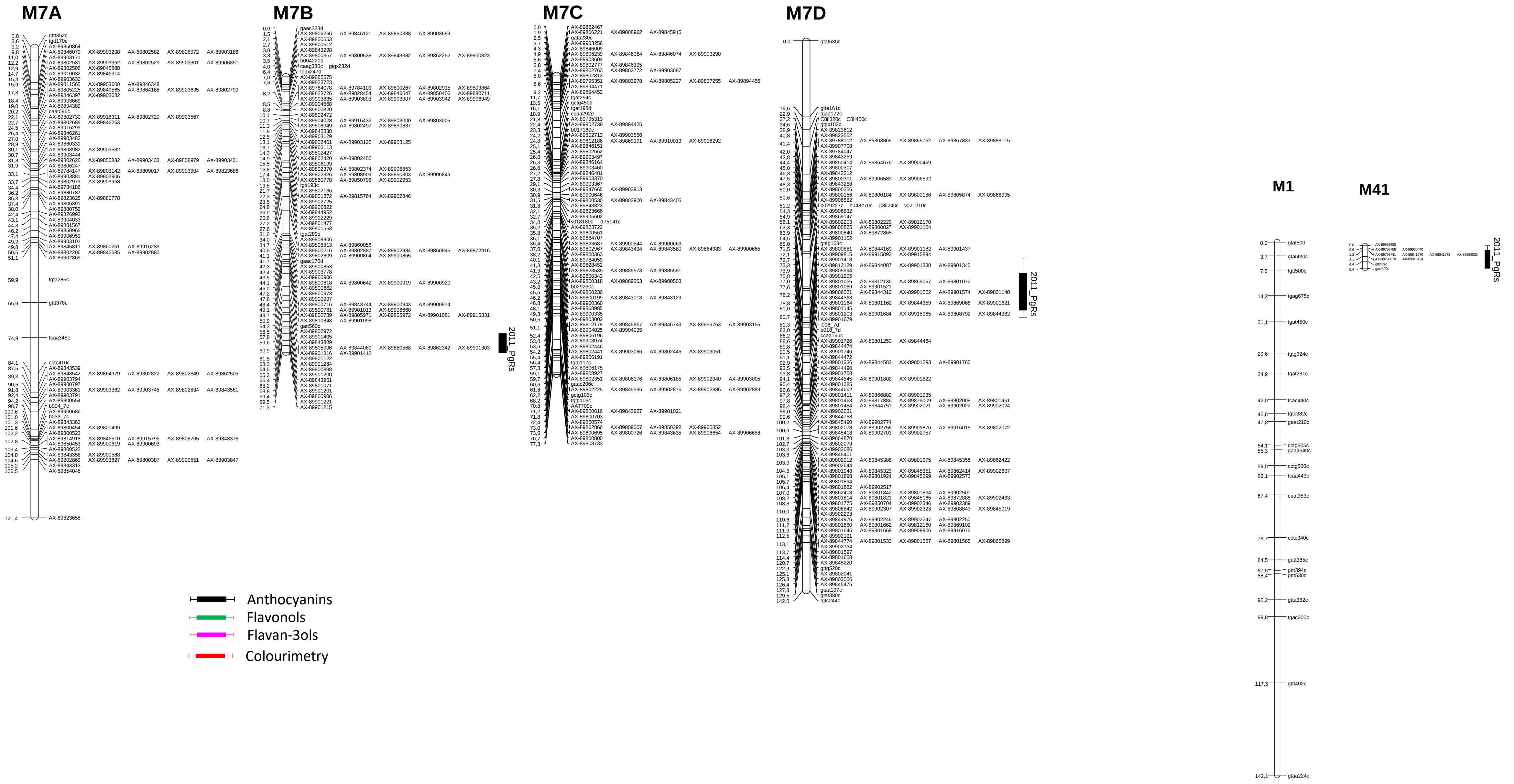

B. QTLs on female linkage map

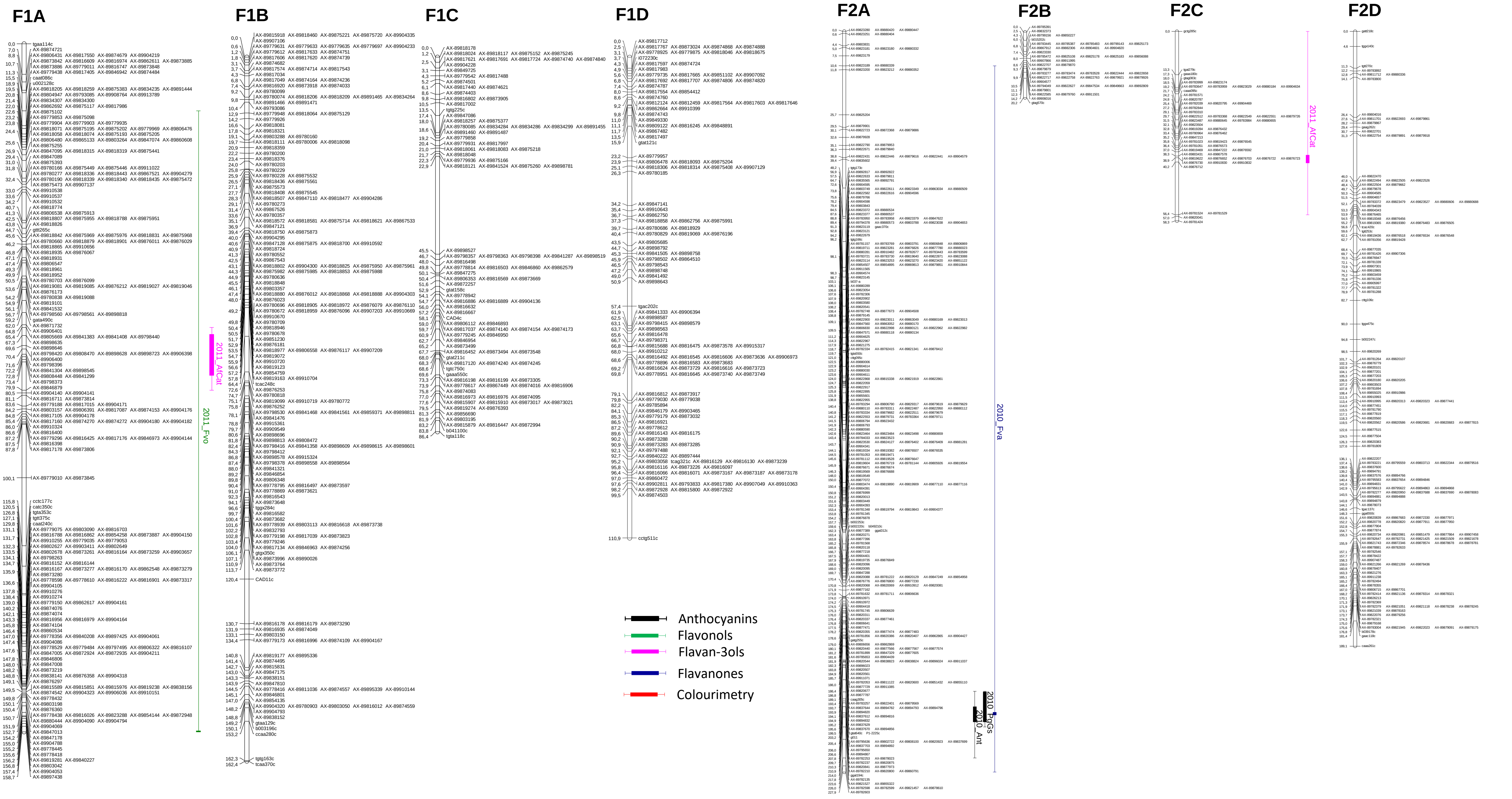

B. QTLs on female linkage map - continued

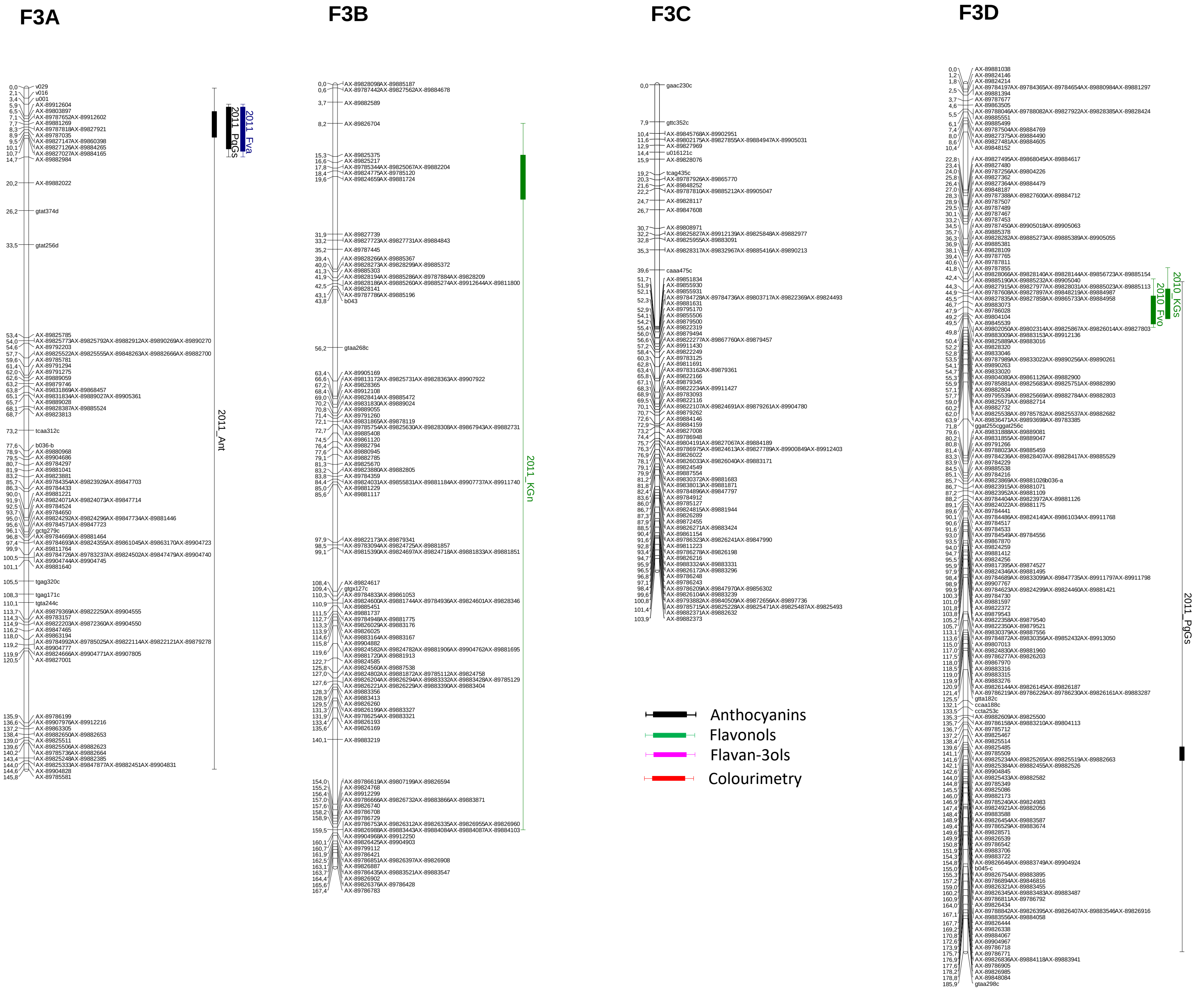

B. QTLs on female linkage map - continued

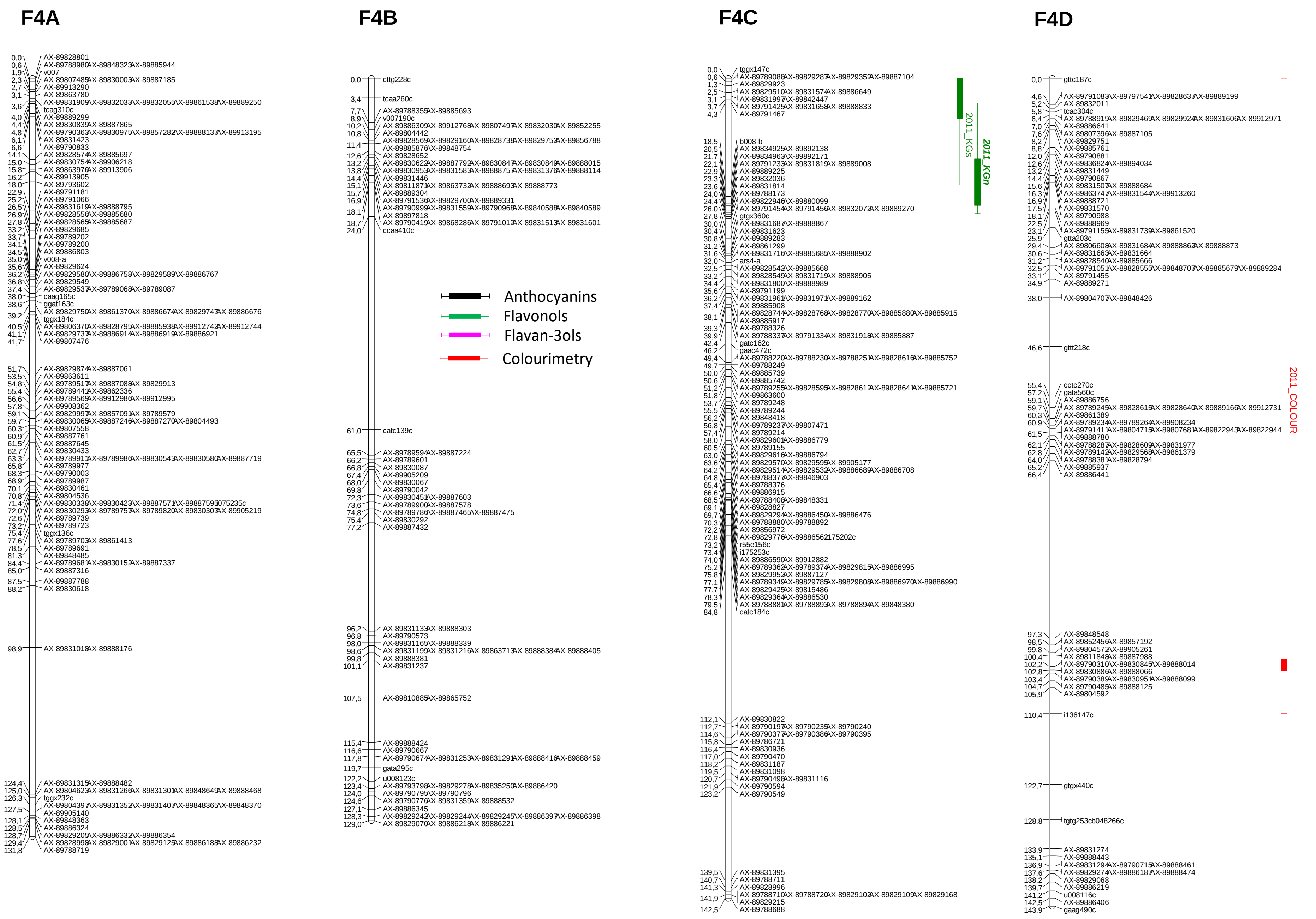

B. QTLs on female linkage map - continued

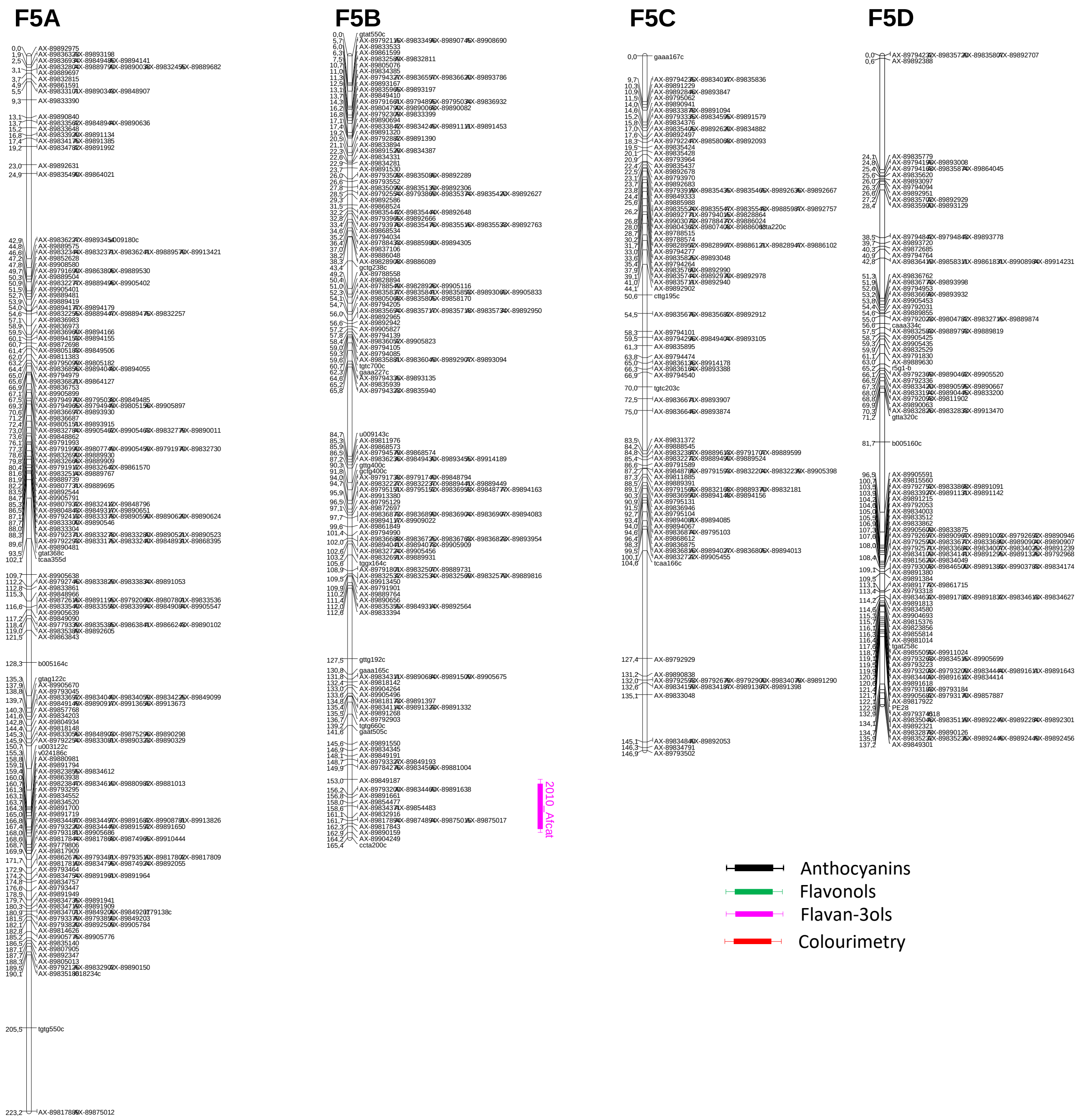

B. QTLs on female linkage map - continued

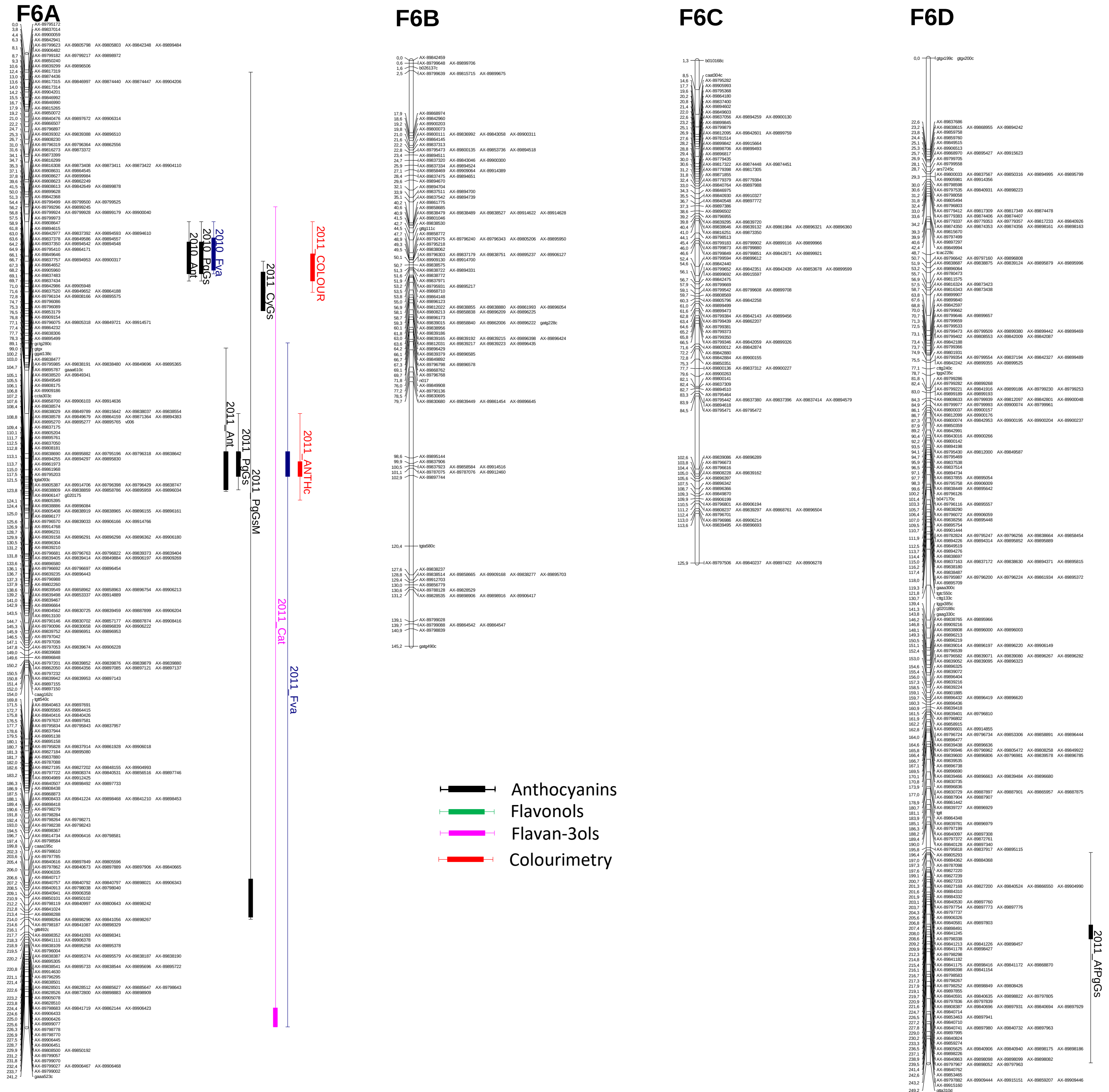

B. QTLs on female linkage map - continued

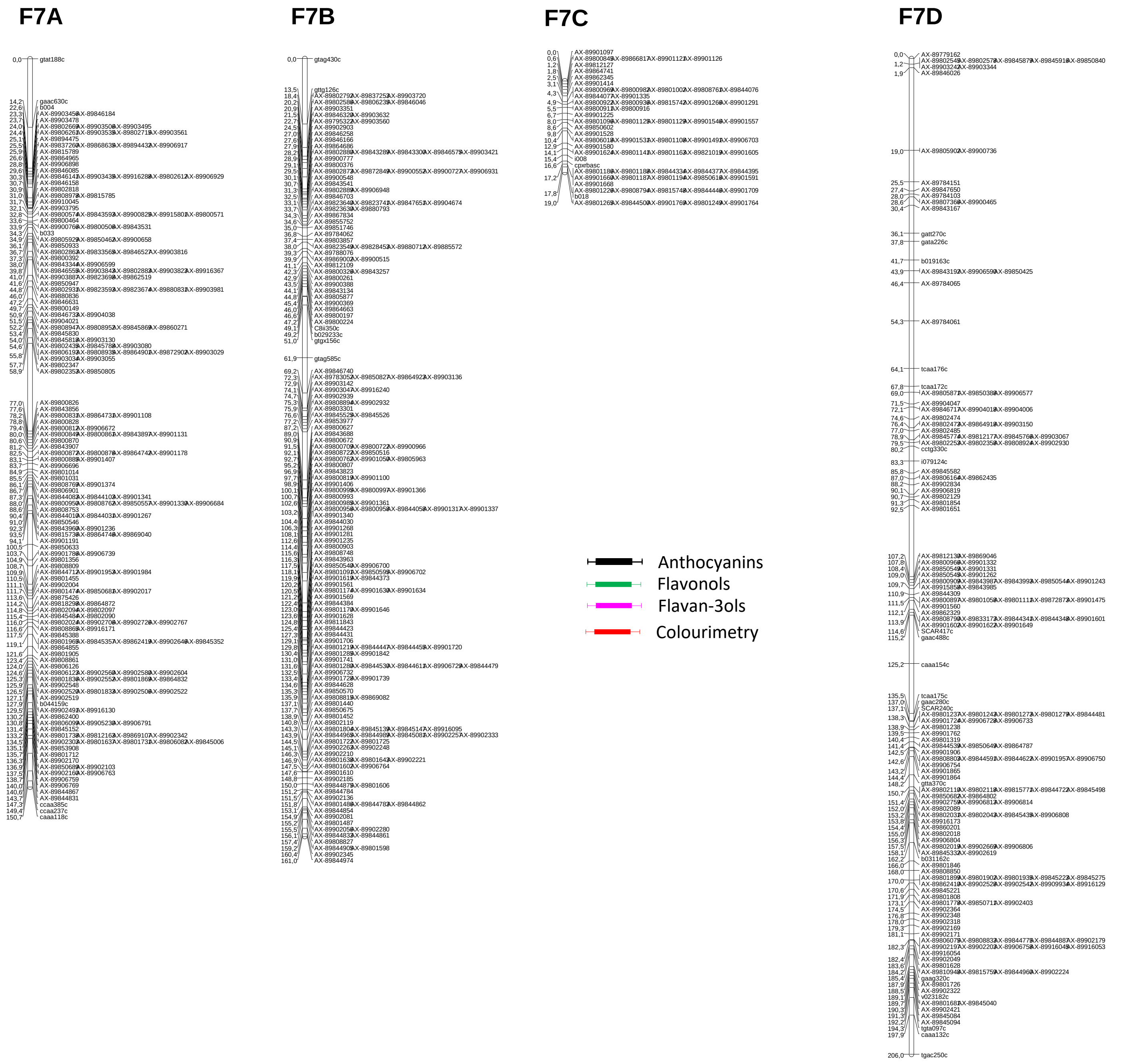

Supplemental Table 1. COLOUR trait values for ‘Capitola’ and ‘Capitola’ x ‘CF1116’ progeny in 2011.

Traits	Abbr.	Capitola			CF1116				Progeny		
		mean	s.d.	range	mean	s.d.	range	Cap vs CF	mean	s.d.	range
Colour	COLOUR	3.50	0.00	3.50-3.50	NA	NA	NA	NA	3.38	1.33	0.50-6.00

The mean value, standard deviation (s.d.) and range are described. ‘CF1116’ was not studied in 2011.
COLOUR values were assessed on a 0 to 6 scale. Values are the means of n = 3 replicates per genotype.

Supplemental Table 2. Correspondence between names of linkage groups from female ('Capitola') and male ('CF1116') parents and the *F. vesca*, *F. viridis*, *F. iinumae* and *F. nipponica* subgenomes according to positions of SNP markers from Axiom 90K SNP array (Hardigan et al., 2020A, 2020b) on the *F. x ananassa* reference genome (Edger et al., 2019).

Female-new-name	Female-old-name	*Camarosa genome*	*Sub-genome*	affyx	gene	rapd	scar	ssr	Total markers	LG size (cM)
F1A	F1b	*Fvb1-4*	*F. vesca*	269		9		1	279	158.67
F1B	F1a	*Fvb1-2*	*F. iinumae*	214	1	7		1	223	162.42
F1C	F1c	*Fvb1-3*	*F. nipponica*	104	1	6		1	112	86.39
F1D	F1d	*Fvb1-1*	*F. viridis*	138		4		1	143	110.87
F2A	F2a	*Fvb2-2*	*F. vesca*	329		10		6	345	227.93
F2B	F2d	*Fvb2-1*	*F. nipponica*	45		1		1	47	20.15
F2C	F2c	*Fvb2-1*	*F. nipponica*	52		5			57	58.29
F2D	F2b	*Fvb2-3*	*F. viridis*	174		12		2	188	189.11
F3A	F3a	*Fvb3-4*	*F. vesca*	117		7		4	128	145.8
F3B	F3d	*Fvb3-2*	*F. iinumae*	175		2		1	178	167.43
F3C	F3c	*Fvb3-3*	*F. nipponica*	124		4		1	129	103.93
F3D	F3b	*Fvb3-1*	*F. viridis*	274		6		2	282	185.9
F4A	F4a	*Fvb4-3*	*F. vesca*	148		6		3	157	131.83
F4B	F4b	*Fvb4-4*	*F. iinumae*	101		6		1	108	128.95
F4C	F4c	*Fvb4-2*	*F. nipponica*	156		5		5	166	142.54
F4D	F4d	*Fvb4-1*	*F. viridis*	104		9		3	116	143.86
F5A	F5a	*Fvb5-1*	*F. vesca*	264		4		6	274	223.16
F5B	F5d	*Fvb5-3*	*F. iinumae*	208		12		1	221	165.44
F5C	F5b	*Fvb5-4*	*F. nipponica*	142		5			147	146.92
F5D	F5c	*Fvb5-2*	*F. viridis*	162		3		4	169	137.19
F6A	F6a	*Fvb6-1*	*F. vesca*	404		11		2	417	241.22
F6B	F6c	*Fvb6-3*	*F. iinumae*	139		4		2	145	145.18
F6C	F6d	*Fvb6-2*	*F. nipponica*	139		1		1	141	125.9
F6D	F6b	*Fvb6-4*	*F. viridis*	349		12		3	364	249.23
F7A	F7c	*Fvb7-2*	*F. vesca*	212		5		3	220	150.74
F7B	F7a	*Fvb7-3*	*F. iinumae*	194		4		2	200	161.04
F7C	F7d	*Fvb7-1*	*F. nipponica*	63			1	2	66	18.98
F7D	F7b	*Fvb7-4*	*F. viridis*	174		14	2	4	194	206
		Total		4974	2	174	3	63	5216	4135.1

Female-new-name	Female-old-name	*Camarosa genome*	*Sub-genome*	affyx	gene	rapd	scar	ssr	Total markers	LG size (cM)
M1A	M1b	*Fvb1-4*	*F. vesca*	39		3			42	28.61
M1B	M1a	*Fvb1-2*	*F. iinumae*	184	1	10		3	198	130.61

M1C	M1c	*Fvb1-3*	*F. nipponica*	104				2	106	82.53
M1D	M1d	*Fvb1-1*	*F. viridis*	43		9		1	53	54.01
M2A	M2a	*Fvb2-2*	*F. vesca*	194		10		3	207	167.07
M2B	M2d	*Fvb2-4*	*F. iinumae*	52		6		2	60	69.27
M2C	M2c	*Fvb2-1*	*F. nipponica*	200		18		5	223	141.88
M2D	M2b	*Fvb2-3*	*F. viridis*	174		10		2	186	136.65
M3A	M3a	*Fvb3-4*	*F. vesca*	271		11		8	290	198.29
M3B	M3d	*Fvb3-2*	*F. iinumae*	216		13		3	232	154.77
M3C1&2	M3c1&c2	*Fvb3-3*	*F. nipponica*	248		8		4	260	138.18
M3D	M3b	*Fvb3-1*	*F. viridis*	129		11		3	143	165.15
M4A	M4a	*Fvb4-3*	*F. vesca*	191		19		8	218	192.23
M4B	M4b	*Fvb4-4*	*F. iinumae*	110		9		3	122	99.82
M4C	M4c	*Fvb4-2*	*F. nipponica*	107	1	12		4	124	174.78
M4D	M4d	*Fvb4-1*	*F. viridis*	97		8		3	108	145.08
M5A	M5a	*Fvb5-1*	*F. vesca*	247		12		6	265	217.01
M5B	M5d	*Fvb5-3*	*F. iinumae*	27		3			30	34.12
M5C	M5b	*Fvb5-4*	*F. nipponica*	257	1	14		5	277	206.74
M5D	M5c	*Fvb5-2*	*F. viridis*	210		9		4	223	128.09
M6A	M6a	*Fvb6-1*	*F. vesca*	312		15		2	329	235.46
M6B	M6c	*Fvb6-3*	*F. iinumae*	21		1		2	24	23.11
M6C	M6d	*Fvb6-2*	*F. nipponica*	230		9		2	241	194.93
M6D	M6b	*Fvb6-4*	*F. viridis*	222		22		1	245	227.9
M7A	M7c	*Fvb7-2*	*F. vesca*	115		7		2	124	121.35
M7B	M7a	*Fvb7-3*	*F. iinumae*	124		8		1	133	71.34
M7C	M7d	*Fvb7-1*	*F. nipponica*	183		10		8	201	142.01
M7D	M7b	*Fvb7-4*	*F. viridis*	122		9		5	136	77.33
		Sub-Total		4429	3	276	0	92	4800	
	Small LGs									
	M41			9		2			11	6.41
	M44	Fvb6-2	*F. nipponica*	43		1		1	45	22.41
	M1					23			23	142.06
		Total		4481	3	302	0	93	4879	

Supplemental Table 3. Summary of male linkage map.

For each linkage group (LG), number of markers, length, mean spacing between markers and max spacing are indicated. LG-new-name corresponds to Hardigan et al., 2020b and LG-old-name corresponds to Labadie et al., 2020.

LG-new-name	LG-old-name	Nb of Markers	length (cM)	mean spacing (cM)	max spacing (cM)
M1A	M1b	42	28.6	0.7	10.6
M1B	M1a	198	130.6	0.7	8.1
M1C	M1c	106	82.5	0.8	21.7
M1D	M1d	53	54	1	11.8
M2A	M2a	207	167.1	0.8	17.4
M2B	M2d	60	69.3	1.2	20.2
M2C	M2c	223	141.9	0.6	5.1
M2D	M2b	186	136.6	0.7	21.8
M3A	M3a	290	198.3	0.7	10
M3B	M3d	232	154.8	0.7	5.4
M3C1	M3c1	131	76.3	0.6	5.8
M3C2	M3c2	129	61.9	0.5	6.2
M3D	M3b	143	165.2	1.2	35.6
M4A	M4a	218	192.2	0.9	12.6
M4B	M4b	122	99.8	0.8	15.9
M4C	M4c	124	174.8	1.4	15.6
M4D	M4d	108	145.1	1.4	32.7
M5A	M5a	265	217	0.8	11.5
M5B	M5d	30	34.1	1.2	7
M5C	M5b	277	206.7	0.7	22.3
M5D	M5c	223	128.1	0.6	12.2
M6A	M6a	329	235.5	0.7	14.6
M6B	M6c	24	23.1	1	10
M6C	M6d	241	194.9	0.8	23.5
M6D	M6b	245	227.9	0.9	12.3
M7A	M7c	124	121.4	1	14.9
M7B	M7a	133	71.3	0.5	5.8
M7C	M7d	201	142	0.7	19.6
M7D	M7b	136	77.3	0.6	6
	M1	23	142.1	6.5	24.6
	M41	11	6.4	0.6	2
	M44	45	22.4	0.5	9.5
	Total or Mean	4879	3929.2	0.8	35.6

Supplemental Table 4. Summary of female linkage map.

For each linkage group (LG), number of markers, length, mean spacing between marker and max spacing are indicated. LG-new-name corresponds to Hardigan et al., 2020b and LG-old-name corresponds to Labadie et al., 2020.

LG-new-name	LG-old-name	Nb of Markers	length (cM)	mean spacing (cM)	max spacing (cM)
F1A	F1b	279	158.7	0.6	15.7
F1B	F1a	223	162.4	0.7	10.3
F1C	F1c	112	86.4	0.8	22.6
F1D	F1d	143	110.9	0.8	11.4
F2A	F2a	345	227.9	0.7	13.9
F2B	F2d	47	20.2	0.4	6
F2C	F2c	57	58.3	1	16.2
F2D	F2b	188	189.1	1	14.7
F3A	F3a	128	145.8	1.1	19.9
F3B	F3d	178	167.4	0.9	13.9
F3C	F3c	129	103.9	0.8	12.1
F3D	F3b	282	185.9	0.7	12.3
F4A	F4a	157	131.8	0.8	25.5
F4B	F4b	108	129	1.2	37
F4C	F4c	166	142.5	0.9	27.3
F4D	F4d	116	143.9	1.3	30.9
F5A	F5a	274	223.2	0.8	18.1
F5B	F5d	221	165.4	0.8	18.9
F5C	F5b	147	146.9	1	22.8
F5D	F5c	169	137.2	0.8	23.5
F6A	F6a	417	241.2	0.6	15.8
F6B	F6c	145	145.2	1	18.9
F6C	F6d	141	124.6	0.9	18.1
F6D	F6b	364	249.2	0.7	22.6
F7A	F7c	220	150.7	0.7	18.1
F7B	F7a	200	161	0.8	13.5
F7C	F7d	66	19	0.3	2.5
F7D	F7b	194	206	1.1	17.2
	Total or Mean	5216	4133.8	0.8	37

Supplemental Table 5. Significant QTLs detected in male and female linkage maps for all traits and for two years based on CIM analysis with LOD> LOD threshold 10% for each trait.
For each trait, the Abbreviation (Abbr.), years, linkage groups (LGs), number of covariable (nb cov) for CIM QTL analyses, QTLs Marker names (Marker) and positions (pos.), LOD score (LOD), effect and R², and flanking QTL markers names (Left and Right) and positions (pos.) are indicated. LG-new-name corresponds to Hardigan et al., 2020b and LG-old-name corresponds to Labadie et al., 2020.

Traits	Abbr.	years	LG-new-name	LG-old-name	nb cov	Marker	Pos.	LOD	Effect	R²	Left flanking marker	Pos.	Right flanking marker	Pos.
**Anthocyanins**														
Total anthocyanins	Ant	2011	M1B	M1a	1	AX-89780748	78.46	4.42	3.97	14.38	AX-89819000	71.52	AX-89860669	80.90
		2010	F2A	F2a	2	AX-89782237	209.65	3.87	-4.44	14.94	AX-89878023	207.80	AX-89821527	223.56
		2010	M3A	M3a	1	AX-89826853	11.37	3.27	4.98	18.89	AX-89904962	8.86	AX-89786111	16.29
		2011	F3A	F3a	2	AX-89787035	8.89	2.66	-3.15	8.77	v029_3a	0.00	AX-89785581	145.80
		2010	F6A	F6a	2	cF6a.loc49.5	49.50	5.02	-5.45	22.17	AX-89899878	41.45	AX-89799499	54.38
		2011	F6A	F6a	2	cF6a.loc101	101.00	2.99	-3.40	10.49	AX-89895575	72.81	AX-89838574	108.42
Pelargonidin-3-glucoside	PgGs	2011	M1B	M1a	2	AX-89780748	78.46	4.34	2.98	11.65	AX-89819013	73.56	cctc227c	86.58
		2010	F2A	F2a	2	cF2a.loc208.5	208.50	4.07	-4.22	14.98	g011_2a	203.19	ggat194c	214.00
		2010	M3A	M3a	1	AX-89826853	11.37	3.60	4.89	20.59	AX-89904962	8.86	AX-89786111	16.29
		2011	F3A	F3a	4	AX-89787035	8.89	3.43	-2.58	8.46	u001_3a	3.40	AX-89882984	14.74
		2011	F3D	F3b	4	cF3b.loc43.5	143.50	2.70	-2.56	8.39	AX-89905040	42.42	gtaa298c	185.90
		2011	M4D	M4d	2	u008113c	138.35	3.05	-2.43	7.56	AX-89886574	82.08	tggx104c	145.08
		2010	F6A	F6a	2	cF6a.loc49.5	49.50	4.47	-4.84	19.70	AX-89899878	41.45	AX-89799499	54.38
		2011	F6A	F6a	4	cF6a.loc103.5	103.50	4.66	-2.66	9.26	gctg280c	89.11	gaaa610c	104.70
Pelargonidin-3-glucoside-malonate	PgGs M	2011	M1C	M1c	1	AX-89860573	0.00	1.93	-0.18	6.54	AX-89860573	0.00	AX-89874552	80.70
			F6A	F6a	1	AX-89840941	209.07	2.65	0.20	8.91	AX-89900059	4.41	AX-89798187	214.58
Pelargonidin-3-rutinoside	PgRs	2011	M1B	M1a	9	cctc227c	86.58	8.29	0.77	12.40	AX-89819033	81.53	tgtt195c	89.71
				M41	9	gatc390c	6.41	5.26	-0.49	5.46	AX-89899035	1.24	gatc390c	6.41
			M5A	M5a	9	cM5a.loc48.5	48.50	6.61	0.65	9.53	AX-89828890	39.96	AX-89794385	56.84
			M5C	M5b	9	tgat148c	63.97	4.54	-0.43	4.15	u009170c	58.96	tgag700c	68.97
			M6A	M6a	9	cM6a.loc104	104.00	4.76	0.60	8.20	AX-89895789	101.85	AX-89796219	110.22
			M6D	M6b	9	AX-89797216	34.09	3.85	-0.61	8.16	cctc218d	30.42	b010162d	40.02
			M6C	M6d	9	AX-89796386	136.33	3.36	-0.71	11.54	gaat480c	0.00	AX-89805325	163.43
			M7B	M7a	9	AX-89901215	71.34	6.24	-0.48	4.90	AX-89843951	66.37	AX-89901215	71.34
			M7C	M7d	9	AX-89901152	64.51	4.16	0.32	2.32	AX-89812170	56.13	AX-89800881	71.47
Cyanidin-3-glucoside	CyGs	2011	F6A	F6a	1	AX-89808647	58.92	4.36	-0.13	14.20	AX-89842368	51.25	AX-89894557	63.63
(epi)Afzelechin-pelargonidin-glucoside	AfPg Gs	2011	M1B	M1a	2	tgaa197c	49.77	3.74	0.10	12.73	AX-89875160	41.65	AX-89875515	55.71
			M2A	M2a	2	AX-89823367	24.66	4.69	0.11	15.19	AX-89783418	21.61	AX-89823385	29.58
			F6D	F6b	1	AX-89798583	216.66	2.73	0.08	8.17	AX-89884362	196.99	gtta310d	249.23
**Flavonols**														
Total Flavonols	Fvo	2011	F1A	F1b	1	AX-89804947	20.75	2.42	-0.03	8.16	caat086c	15.45	AX-89897438	158.67
		2010	F3D	F3b	1	AX-89827835	45.49	2.83	-0.07	16.54	AX-89787855	41.81	AX-89828320	52.24
Kaempferol-glucoside	KGs	2010	F3D	F3b	1	AX-89827835	45.49	3.01	-0.03	17.50	AX-89787765	39.35	AX-89883016	50.39
		2011	F4C	F4c	1	AX-89829510	2.48	4.09	0.02	13.39	tggx147c_F4c	0.00	b008-b_4c	18.53
Kaempferol-glucuronide	KGn	2011	F3B	F3d	2	cF3d.loc21	21.00	2.66	0.01	6.20	AX-89826704	8.17	AX-89786753	159.46
			M4C	M4c	2	cM4c.loc1	1.00	5.08	-0.02	16.37	AX-89791452	0.00	gtat238c	10.14
			F4C	F4c	2	cF4c.loc18.5	18.50	5.56	-0.01	15.39	AX-89791467	4.31	AX-89889008	22.08

			M6C	M6d	2	AX-89805325	163.43	3.11	-0.01	11.01	AX-89796107	150.67	tcaa540c	186.97
Kaempferol-coumaryl-glucoside	KCoGs	2011	M5D	M5c	1	AX-89835889	19.97	3.08	-0.01	10.26	tgtt140c	13.29	AX-89794844	25.75
		2010	M6D	M6b	1	AX-89896029	36.12	3.27	-0.02	18.88	cctc218d	30.42	tcac235c	45.98
Quercetin-glucuronide	QGn	2011	M6C	M6d	1	AX-89805325	163.43	2.54	-0.01	8.53	gaat480c	0.00	caaa167c	194.93
**Flavan-3-ols**														
Total Flavan-3-ols	F3ol	2011	M3C1	M3c1	2	AX-89785338	16.23	3.30	0.24	10.33	i075224c	11.32	AX-89904746	19.29
		2010	M5A	M5a	2	cM5a.loc23.5	23.50	2.85	-0.25	13.19	AX-89793472	22.63	gtgx171c	33.17
		2011	M5A	M5a	2	AX-89835724	46.80	5.08	-0.30	15.75	AX-89828890	39.96	AX-89794385	56.84
		2010	M6A	M6a	2	AX-89796438	123.15	4.22	-0.28	16.99	tgat340c	116.22	AX-89914769	128.09
Catechin	Cat	2011	F6A	F6a	1	cF6a.loc240.5	240.50	2.61	0.03	8.76	AX-89899984	37.75	gaaa523c	241.22
(epi)Catechin dimers	CatCat	2011	M2C	M2c	1	AX-89782622	86.22	3.79	-0.09	12.47	AX-89865363	78.73	i146188c	88.67
(epi)Afzelechin-(epi)catechin dimers	AfCat	2011	M1B	M1a	4	AX-89873448	123.16	5.51	0.02	18.42	AX-89816417	118.16	AX-89864948	125.60
		2011	F1A	F1b	2	AX-89798396	71.58	3.18	0.01	9.39	AX-89798440	65.40	AX-89846879	79.87
		2011	F2C	F2c	2	AX-89819622	38.88	2.05	-0.01	5.69	gctg285c	0.00	AX-89781524	56.43
		2011	M5A	M5a	4	cM5a.loc47	47.00	3.16	-0.01	5.09	AX-89828890	39.96	AX-89794385	56.84
		2011	M5C	M5b	4	AX-89836519	38.05	5.52	0.02	18.18	AX-89794083	31.26	AX-89794556	39.88
		2010	F5B	F5d	1	cF5d.loc158.5	158.50	3.16	-0.01	18.26	AX-89849187	153.04	AX-89904249	164.15
		2011	M6A	M6a	4	AX-89914769	128.09	5.38	0.02	15.84	AX-89795266	120.71	AX-89914804	129.92
(epi)Afzelechin-glucoside	AfGs	2011	M3A	M3a	3	cM3a.loc30.5	30.50	4.03	0.19	9.70	AX-89785262	20.73	AX-89784824	33.27
		2011	M5A	M5a	3	cM5a.loc58	58.00	3.50	-0.19	9.68	AX-89905829	46.80	b037227c	64.95
		2010	M6A	M6a	1	cM6a.loc126	126.00	2.71	-0.24	15.96	catc138c	49.50	AX-89840892	205.99
		2011	M6A	M6a	3	cM6a.loc165	165.00	5.82	-0.25	16.31	tcaa168c	153.98	AX-89797755	168.49
**Colourimetry**														
Anthocyanins (colourimetry)	ANTHc	2011	M1B	M1a	2	AX-89816735	36.09	3.09	130.41	9.91	i072226c	2.54	AX-89875150	41.00
		2010	M4A	M4a	1	tgaa125c	104.80	2.86	166.41	17.87	gatc380c	0.00	gtta105c	165.84
		2011	M6A	M6a	2	AX-89840892	205.99	5.19	167.22	16.31	AX-89797823	200.76	AX-89895308	220.62
		2011	F6A	F6a	1	cF6a.loc103.5	103.50	5.63	-176.14	17.97	gctg280c	89.11	AX-89795985	104.70
Colour	COLOUR	2011	M1B	M1a	2	v017213c	52.64	4.80	0.89	11.21	AX-89875160	41.65	AX-89780210	53.87
			M3A	M3a	2	cM3a.loc9.5	9.50	4.08	0.85	10.10	AX-89826900	8.86	AX-89804053	18.77
			F4D	F4d	2	AX-89790310	102.21	2.89	0.80	8.99	gttc187c	0.00	i136147c	110.44
			F6A	F6a	2	cF6a.loc53.5	53.50	3.61	-0.89	11.06	AX-89899878	41.45	AX-89899245	56.23

Supplemental Table 6. Values of the QTL thresholds at 5 and 10% used for CIM analysis. The thresholds were calculated on 1000 permutations for each trait for male and female in 2010 and 2011.

Traits	Abbr.	Male threshold				Female threshold			
		2010		2011		2010		2011	
		5%	10%	5%	10%	5%	10%	5%	10%
**Anthocyanins**									
Total anthocyanins	Ant	3.07	2.79	3.16	2.73	3.23	2.84	2.13	2.12
Pelargonidin-3-glucoside	PgGs	3.05	2.75	3.21	2.80	3.17	2.86	2.25	2.16
Pelargonidin-3-glucoside-malonate	PgGsM	2.04	1.87	1.92	1.78	2.13	1.97	1.78	1.75
Pelargonidin-3-rutinoside	PgRs	2.79	2.55	3.17	2.76	3.07	2.81	2.85	2.72
Cyanidin-3-glucoside	CyGs	2.80	2.54	3.13	2.89	2.89	2.64	2.61	2.60
(epi)Afzelechin-pelargonidin-glucoside	AfPgGs	3.23	2.89	3.09	2.78	3.29	2.94	2.51	2.45
**Flavonols**									
Total Flavonols	Fvo	2.93	2.64	3.08	2.77	2.95	2.73	2.02	2.01
Kaempferol-glucoside	KGs	3.11	2.73	3.01	2.75	3.17	2.84	2.04	2.01
Kaempferol-glucuronide	KGn	3.03	2.77	3.20	2.85	3.05	2.78	2.16	2.11
Kaempferol-coumaryl-glucoside	KCoGs	3.19	2.71	3.22	2.89	3.21	2.88	3.21	3.08
Quercetin-glucuronide	QGn	2.28	2.10	3.04	2.78	2.29	2.12	2.43	2.40
**Flavan-3-ols**									
Total Flavan-3-ols	F3ol	2.77	2.57	3.04	2.78	3.00	2.71	3.45	3.23
Catechin	Cat	3.02	2.65	3.02	2.80	3.25	2.80	2.68	2.63
(epi)Catechin dimers	CatCat	3.15	2.84	3.05	2.76	3.23	2.91	1.96	1.95
(epi)Afzelechin-(epi)catechin dimers	AfCat	2.97	2.67	3.12	2.79	3.22	2.89	1.89	1.87
(epi)Afzelechin-glucoside	AfGs	2.82	2.54	3.07	2.75	3.09	2.76	2.16	2.15
**Colourimetry**									
Anthocyanins (colourimetry)	ANTHc	3.11	2.77	3.15	2.80	3.19	2.87	5.15	4.77
**Visual assessment**									
Colour	COLOUR	NA	NA	3.07	2.79	NA	NA	2.42	2.41

Supplementary Table S7. List of genes from the FvH4_v4.0.a2 version of the *Fragaria vesca* reference genome located in the M3A/F3A colour-related QTLs (PgGs etc): physical position from 826.085 b to 2.673762 b (extract from Li et al., 2019, Sup Table S2)

Gene ID (v2.	Gene location	LG	Start	End	Gene ID (v4.0.a1	Gene ID (v2.	AED score	GO term	symbol	Description v4.0.a2
gene39832	Fvb3:842903-843127	Fvb3	842903	843127	FvH4_3g01591	gene39832	1			
gene19488	Fvb3:844553-846799	Fvb3	844553	846799	FvH4_3g01610	gene19488	0.02	GO:0005515		Tetratricopeptide repeat (TPR)-like superfamily protein
gene36080	Fvb3:847082-849454	Fvb3	847082	849454	FvH4_3g01611	gene36080	0.27	GO:0004650,GO:0005975		Pectin lyase-like superfamily protein
gene36081	Fvb3:850818-852643	Fvb3	850818	852643	FvH4_3g01620	gene36081	0.31	GO:0004650,GO:0005975		Pectin lyase-like superfamily protein
gene19486	Fvb3:852844-855245	Fvb3	852844	855245	FvH4_3g01630	gene19486	0.07	GO:0030170		Predicted pyridoxal phosphate-dependent enzyme, YBL036C type
gene19485	Fvb3:856439-857203	Fvb3	856439	857203	FvH4_3g01640	gene19485	0.78			Stigma-specific Stig1 family protein
gene19484	Fvb3:858564-860252	Fvb3	858564	860252	FvH4_3g01660	gene19484	0.53			Stigma-specific Stig1 family protein
gene19483	Fvb3:861992-863872	Fvb3	861992	863872	FvH4_3g01670	gene19483	0.47			Stigma-specific Stig1 family protein
gene19482	Fvb3:864360-864793	Fvb3	864360	864793	FvH4_3g01671	gene19482	1			
gene19481	Fvb3:865701-866343	Fvb3	865701	866343	FvH4_3g01672	gene19481	0.6			Stigma-specific Stig1 family protein
gene19480	Fvb3:866815-869961	Fvb3	866815	869961	FvH4_3g01680	gene19480	0.34			Pectin lyase-like superfamily protein
gene19479	Fvb3:868323-869998	Fvb3	868323	869998	FvH4_3g01690	gene19479	0.02	GO:0030247		
gene19478	Fvb3:877530-880706	Fvb3	877530	880706	FvH4_3g01700	gene19478	0.1	GO:0003700,(	ATWRKY6,WRKY6	WRKY family transcription factor
gene19477	Fvb3:881776-885746	Fvb3	881776	885746	FvH4_3g01710	gene19477	0.24	GO:0004190,(	APA1,ATAPA1	aspartic proteinase A1
gene19476	Fvb3:887358-888988	Fvb3	887358	888988	FvH4_3g01720	gene19476	0.2	GO:0004672,(	MAPKKK16	mitogen-activated protein kinase kinase kinase 16
gene19475	Fvb3:889181-892415	Fvb3	889181	892415	FvH4_3g01730	gene19475	0.08	GO:0003677,(	ATTOPII,TOPII	topoisomerase II
gene19474	Fvb3:896142-897214	Fvb3	896142	897214	FvH4_3g01740	gene19474	0.04		SLP3	subtilisin-like serine protease 3
gene19473	Fvb3:899967-901443	Fvb3	899967	901443	FvH4_3g01741	gene19473	1			
gene19472	Fvb3:901962-903170	Fvb3	901962	903170	FvH4_3g01742	gene19472	0	GO:0005783,GO:0006886,GO:00160		B-cell receptor-associated 31-like
gene19471	Fvb3:903802-904234	Fvb3	903802	904234	FvH4_3g01743	gene19471	1	GO:0003723		RNA-binding KH domain-containing protein
gene19470	Fvb3:904187-906850	Fvb3	904187	906850	FvH4_3g01750	gene19470	0.05	GO:0005783,GO:0006886,GO:00160		B-cell receptor-associated 31-like
gene19469	Fvb3:907610-909061	Fvb3	907610	909061	FvH4_3g01760	gene19469	0.01	GO:0008152,GO:0008168		S-adenosyl-L-methionine-dependent methyltransferases superfamily protein
gene19647	Fvb3:920697-923085	Fvb3	920697	923085	FvH4_3g01780	gene19647	0.03	GO:0008152,GO:0008168		S-adenosyl-L-methionine-dependent methyltransferases superfamily protein
gene19646	Fvb3:923544-924854	Fvb3	923544	924854	FvH4_3g01790	gene19646	0.12			
gene19645	Fvb3:927006-930248	Fvb3	927006	930248	FvH4_3g01800	gene19645	0.15	GO:0016021		Peroxisomal membrane 22 kDa (Mpv17/PMP22) family protein
gene19644	Fvb3:931371-932945	Fvb3	931371	932945	FvH4_3g01810	gene19644	0.08			F-box family protein with a domain of unknown function (DUF295)
gene19643	Fvb3:933825-937208	Fvb3	933825	937208	FvH4_3g01820	gene19643	0.2	GO:0004047,GO:0005515,GO:00065		Glycine cleavage T-protein family
gene19641	Fvb3:937913-939770	Fvb3	937913	939770	FvH4_3g01830	gene19641	0	GO:0003677		Homeodomain-like superfamily protein
gene19640	Fvb3:945420-949637	Fvb3	945420	949637	FvH4_3g01840	gene19640	0.09	GO:0005507,(	sks4	SKU5 similar 4
gene19639	Fvb3:950160-953560	Fvb3	950160	953560	FvH4_3g01850	gene19639	1	GO:0003824,GO:0005975,GO:00302		Rhamnogalacturonate lyase family protein
gene19638	Fvb3:954306-958361	Fvb3	954306	958361	FvH4_3g01860	gene19638	0.05			endonuclease/exonuclease/phosphatase family protein
gene36082	Fvb3:959559-961661	Fvb3	959559	961661	FvH4_3g01870	gene36082	0.05	GO:0000160	AHP1	histidine-containing phosphotransmitter 1
gene36083	Fvb3:962029-965180	Fvb3	962029	965180	FvH4_3g01880	gene36083	0.03	GO:0004553,GO:0005975		O-Glycosyl hydrolases family 17 protein
gene39833	Fvb3:965754-966752	Fvb3	965754	966752	FvH4_3g01890	gene39833	0			
gene19636	Fvb3:967939-970787	Fvb3	967939	970787	FvH4_3g01900	gene19636	0.03	GO:0016671,(	APR3,ATAPR3,PRH-26	APS reductase 3
gene19635	Fvb3:975062-976231	Fvb3	975062	976231	FvH4_3g01910	gene19635	0.11	GO:0003735,GO:0005840,GO:00064		60S acidic ribosomal protein family
gene19634	Fvb3:978864-981677	Fvb3	978864	981677	FvH4_3g01920	gene19634	0.31	GO:0004462,(	ATGLX1,GLX1	glyoxalase I homolog
gene19633	Fvb3:981811-983217	Fvb3	981811	983217	FvH4_3g01921	gene19633	0.32	GO:0006355	SNL1	SIN3-like 1
gene19632	Fvb3:984124-985287	Fvb3	984124	985287	FvH4_3g01930	gene19632	1	GO:0006355	ATSIN3,SIN3,SNL3	SIN3-like 3
gene19631	Fvb3:985544-990088	Fvb3	985544	990088	FvH4_3g01940	gene19631	0.12	GO:0004664,(	ADT1	arogenate dehydratase 1
gene19630	Fvb3:992934-995725	Fvb3	992934	995725	FvH4_3g01950	gene19630	0.04	GO:0005515		Ankyrin repeat family protein
gene39834	Fvb3:995867-997256	Fvb3	995867	997256	FvH4_3g01970	gene39834	0.03			ankyrin repeat family protein
gene19629	Fvb3:997175-999038	Fvb3	997175	999038	FvH4_3g01980	gene19629	0.03		APG8A,ATG8A	Ubiquitin-like superfamily protein
gene39835	Fvb3:1005208-1007252	Fvb3	1005208	1007252	FvH4_3g01990	gene39835	0.61			
gene19628	Fvb3:1008033-1010382	Fvb3	1008033	1010382	FvH4_3g02000	gene19628	0.04	GO:0004097,GO:0055114		
gene19627	Fvb3:1012397-1013018	Fvb3	1012397	1013018	FvH4_3g02010	gene19627	0.2			
gene19626	Fvb3:1013753-1017178	Fvb3	1013753	1017178	FvH4_3g02020	gene19626	0.13	GO:0006486,GO:0008378,GO:00160		Galactosyltransferase family protein
gene36084	Fvb3:1018898-1020868	Fvb3	1018898	1020868	FvH4_3g02030	gene36084	0.25			Protein of unknown function, DUF584
gene19625	Fvb3:1023652-1032269	Fvb3	1023652	1032269	FvH4_3g02040	gene19625	0.12	GO:0004373,(	ATSS3,SS3	starch synthase 3
gene19624	Fvb3:1032973-1033354	Fvb3	1032973	1033354	FvH4_3g02050	gene19624	1			
gene19623	Fvb3:1033896-1035017	Fvb3	1033896	1035017	FvH4_3g02060	gene19623	0.42	GO:0015986,(	ATPC1	ATPase, F1 complex, gamma subunit protein
gene19622	Fvb3:1035439-1043274	Fvb3	1035439	1043274	FvH4_3g02070	gene19622	0.14	GO:0003677	DUO3	Homeodomain-like superfamily protein
gene19620	Fvb3:1048889-1049564	Fvb3	1048889	1049564	FvH4_3g02071	gene19620	0.32	GO:0008420,(	CPL4	C-terminal domain phosphatase-like 4
gene36085	Fvb3:1053256-1058542	Fvb3	1053256	1058542	FvH4_3g02080	gene36085	0.05	GO:0005515		Met-10+ like family protein / kelch repeat-containing protein
gene36086	Fvb3:1061045-1063286	Fvb3	1061045	1063286	FvH4_3g02090	gene36086	0.12	GO:0004601,(	PRXR1	Peroxidase superfamily protein
gene19618	Fvb3:1063611-1067660	Fvb3	1063611	1067660	FvH4_3g02100	gene19618	0			
gene19617	Fvb3:1070692-1071396	Fvb3	1070692	1071396	FvH4_3g02101	gene19617	1			F-box family protein
gene19615	Fvb3:1072228-1077275	Fvb3	1072228	1077275	FvH4_3g02130	gene19615	0.16	GO:0004672,(	CPK21	calcium-dependent protein kinase 21
gene19614	Fvb3:1077065-1078269	Fvb3	1077065	1078269	FvH4_3g02131	gene19614	0.91			
gene19613	Fvb3:1080746-1081375	Fvb3	1080746	1081375	FvH4_3g02140	gene19613	1			Protein of unknown function, DUF584
gene19612	Fvb3:1082259-1085472	Fvb3	1082259	1085472	FvH4_3g02150	gene19612	1			
gene19611	Fvb3:1089090-1090099	Fvb3	1089090	1090099	FvH4_3g02160	gene19611	0			
gene19608	Fvb3:1104055-1104642	Fvb3	1104055	1104642	FvH4_3g02170	gene19608	1			oxidoreductase, zinc-binding dehydrogenase family protein
gene19607	Fvb3:1106898-1110805	Fvb3	1106898	1110805	FvH4_3g02171	gene19607	1			
gene19606	Fvb3:1113776-1119852	Fvb3	1113776	1119852	FvH4_3g02190	gene19606	0.04	GO:0016491,GO:0055114		oxidoreductase, zinc-binding dehydrogenase family protein
gene19604	Fvb3:1122569-1124236	Fvb3	1122569	1124236	FvH4_3g02200	gene19604	1	GO:0000271,GO:0003979,GO:00512		UDP-glucose 6-dehydrogenase family protein
gene19603	Fvb3:1125468-1126183	Fvb3	1125468	1126183	FvH4_3g02201	gene19603	1			
gene19602	Fvb3:1127095-1129662	Fvb3	1127095	1129662	FvH4_3g02210	gene19602	0.07			
gene19621	Fvb3:1132001-1132390	Fvb3	1132001	1132390	FvH4_3g02220	gene19621	1			
gene36087	Fvb3:1136867-1140924	Fvb3	1136867	1140924	FvH4_3g02240	gene36087	0.09			
gene36088	Fvb3:1146381-1150096	Fvb3	1146381	1150096	FvH4_3g02250	gene36088	0.33	GO:0006855,GO:0015238,GO:00152		MATE efflux family protein
gene19600	Fvb3:1150471-1154816	Fvb3	1150471	1154816	FvH4_3g02260	gene19600	0.28	GO:0006855,GO:0015238,GO:00152		MATE efflux family protein
gene19599	Fvb3:1155287-1155909	Fvb3	1155287	1155909	FvH4_3g02270	gene19599	1			
gene19598	Fvb3:1159211-1161427	Fvb3	1159211	1161427	FvH4_3g02280	gene19598	0.03	GO:0003677,(	ATSWI3B,CHB2,SWI3E	switch subunit 3
gene19597	Fvb3:1161199-1165626	Fvb3	1161199	1165626	FvH4_3g02290	gene19597	0.93	GO:0005515,GO:0043531		NB-ARC domain-containing disease resistance protein
gene19596	Fvb3:1166594-1169644	Fvb3	1166594	1169644	FvH4_3g02300	gene19596	0.06	GO:0016226	ABC1,ATABC1,ATNAP:	ATP binding cassette protein 1
gene19595	Fvb3:1170407-1174072	Fvb3	1170407	1174072	FvH4_3g02310	gene19595	0			DNA binding
gene19594	Fvb3:1175337-1179005	Fvb3	1175337	1179005	FvH4_3g02320	gene19594	0.22	GO:0003676	ATRBP45B,RBP45B	RNA-binding (RRM/RBD/RNP motifs) family protein
gene19593	Fvb3:1179842-1183164	Fvb3	1179842	1183164	FvH4_3g02321	gene19593	0.08			
gene19592	Fvb3:1182925-1183445	Fvb3	1182925	1183445	FvH4_3g02330	gene19592	0.44			HSP20-like chaperones superfamily protein
gene19591	Fvb3:1184010-1192131	Fvb3	1184010	1192131	FvH4_3g02340	gene19591	0.01	GO:0005515,GO:0007051		binding;calmodulin binding
gene19590	Fvb3:1192787-1195424	Fvb3	1192787	1195424	FvH4_3g02350	gene19590	0.14		DER2.2	DERLIN-2.2
gene39836	Fvb3:1195454-1199552	Fvb3	1195454	1199552	FvH4_3g02360	gene39836	0.33		ATTOM1,TOM1	tobamovirus multiplication 1
gene19588	Fvb3:1202464-1204303	Fvb3	1202464	1204303	FvH4_3g02370	gene19588	0.13	GO:0005515	PPR336	pentatricopeptide repeat 336
gene19587	Fvb3:1204860-1205587	Fvb3	1204860	1205587	FvH4_3g02390	gene19587	1	GO:0016616,GO:0051287,GO:00551		UDP-glucose 6-dehydrogenase family protein
gene19586	Fvb3:1205926-1208911	Fvb3	1205926	1208911	FvH4_3g02400	gene19586	0	GO:0005515		SWIB complex BAF60b domain-containing protein
gene36089	Fvb3:1209686-1214038	Fvb3	1209686	1214038	FvH4_3g02410	gene36089	0.12	GO:0005524	ATHSP101,HOT1,HSP1	heat shock protein 101
gene36090	Fvb3:1214342-1217361	Fvb3	1214342	1217361	FvH4_3g02420	gene36090	0.11	GO:0000741		
gene19584	Fvb3:1217959-1218596	Fvb3	1217959	1218596	FvH4_3g02421	gene19584	1			
gene19583	Fvb3:1218676-1220955	Fvb3	1218676	1220955	FvH4_3g02430	gene19583	0.11	GO:0008654,(	CLS	cardiolipin synthase
gene19582	Fvb3:1221719-1224257	Fvb3	1221719	1224257	FvH4_3g02440	gene19582	0.2	GO:0004672,GO:0005524,GO:00064		Protein kinase superfamily protein
gene19581	Fvb3:1223868-1224263	Fvb3	1223868	1224263	FvH4_3g02450	gene19581	1			
gene19580	Fvb3:1225360-1232457	Fvb3	1225360	1232457	FvH4_3g02460	gene19580	0.13	GO:0005515,GO:0006629		phospholipases;galactolipases
gene19579	Fvb3:1233807-1234424	Fvb3	1233807	1234424	FvH4_3g02470	gene19579	1			LA RNA-binding protein
gene19578	Fvb3:1235098-1238130	Fvb3	1235098	1238130	FvH4_3g02490	gene19578	0.26	GO:0019239		adenosine/AMP deaminase family protein
gene19577	Fvb3:1237674-1242642	Fvb3	1237674	1242642	FvH4_3g02480	gene19577	0.09	GO:0001522,GO:0003723,GO:00099		Pseudouridine synthase family protein
gene36091	Fvb3:1241491-1247952	Fvb3	1241491	1247952	FvH4_3g02481	gene36091	0.11	GO:0004553,(	BGLU47	beta-glucosidase 47
gene36092	Fvb3:1248042-1251710	Fvb3	1248042	1251710	FvH4_3g02500	gene36092	0.22	GO:0016021,(	ATGPT1,GPT1	glucose 6-phosphate/phosphate translocator 1
gene19573	Fvb3:1255971-1262076	Fvb3	1255971	1262076	FvH4_3g02510	gene19573	0.08	GO:0003676	PCFS4	PCF11P-similar protein 4
gene19572	Fvb3:1267960-1273443	Fvb3	1267960	1273443	FvH4_3g02520	gene19572	0.23	GO:0006355,(	PDF2	protodermal factor 2
gene19570	Fvb3:1279701-1282170	Fvb3	1279701	1282170	FvH4_3g02530	gene19570	0	GO:0010215,(	COBL11	COBRA-like protein 11 precursor
gene19568	Fvb3:1282882-1283827	Fvb3	1282882	1283827	FvH4_3g02550	gene19568	0.25		NTL	NTF2-like
gene19567	Fvb3:1284357-1286597	Fvb3	1284357	1286597	FvH4_3g02560	gene19567	0.1	GO:0004857,(	ATPMEPCRA,PMEPCR.	methylesterase PCR A
gene19566	Fvb3:1289201-1292633	Fvb3	1289201	1292633	FvH4_3g02570	gene19566	0.58		RIC7	PAK-box/P21-Rho-binding family protein
gene19564	Fvb3:1293444-1294926	Fvb3	1293444	1294926	FvH4_3g02571	gene19564	1			
gene19563	Fvb3:1298132-1298446	Fvb3	1298132	1298446	FvH4_3g02572	gene19563	1		ATFC-II,FC-II,FC2	ferrochelatase 2
gene19562	Fvb3:1301418-1303514	Fvb3	1301418	1303514	FvH4_3g02580	gene19562	1	GO:0005515		F-box/RNI-like superfamily protein

gene19561	Fvb3:1304903-1305370	Fvb3	1304903	1305370	FvH4_3g02581	gene19561	1		ACS12	1-amino-cyclopropane-1-carboxylate synthase 12
gene39837	Fvb3:1306058-1307635	Fvb3	1306058	1307635	FvH4_3g02582	gene39837	0.1	GO:0003676		
gene19560	Fvb3:1307821-1309174	Fvb3	1307821	1309174	FvH4_3g02590	gene19560	0.44			
gene19559	Fvb3:1312993-1315394	Fvb3	1312993	1315394	FvH4_3g02591	gene19559	0.15	GO:0005515		F-box/RNI-like superfamily protein
gene39838	Fvb3:1316535-1318003	Fvb3	1316535	1318003	FvH4_3g02592	gene39838	1			
gene19558	Fvb3:1318229-1319213	Fvb3	1318229	1319213	FvH4_3g02593	gene19558	0.43			
gene19557	Fvb3:1322663-1323853	Fvb3	1322663	1323853	FvH4_3g02594	gene19557	0.14	GO:0005515		F-box family protein
gene19556	Fvb3:1324979-1325998	Fvb3	1324979	1325998	FvH4_3g02600	gene19556	0.33			Oligosaccharyltransferase complex/magnesium transporter family protein
gene19555	Fvb3:1326638-1327957	Fvb3	1326638	1327957	FvH4_3g02620	gene19555	0.27	GO:0005515		Tetratricopeptide repeat (TPR)-like superfamily protein
gene19553	Fvb3:1331732-1333854	Fvb3	1331732	1333854	FvH4_3g02630	gene19553	0.08	GO:0004553,(	XTH8	xyloglucan endotransglucosylase/hydrolase 8
gene19552	Fvb3:1334771-1336329	Fvb3	1334771	1336329	FvH4_3g02640	gene19552	0			postsynaptic protein-related
gene19551	Fvb3:1337019-1340760	Fvb3	1337019	1340760	FvH4_3g02641	gene19551	1	GO:0005515	UBQ10	polyubiquitin 10
gene19550	Fvb3:1340958-1345868	Fvb3	1340958	1345868	FvH4_3g02650	gene19550	0.11	GO:0005515,(	TDP1	tyrosyl-DNA phosphodiesterase-related
gene36093	Fvb3:1347333-1351040	Fvb3	1347333	1351040	FvH4_3g02660	gene36093	0.19			Chaperone DnaJ-domain superfamily protein
gene36094	Fvb3:1351967-1355057	Fvb3	1351967	1355057	FvH4_3g02670	gene36094	0.02	GO:0016491,(	ATGA2OX8,GA2OX8	gibberellin 2-oxidase 8
gene19547	Fvb3:1360388-1360757	Fvb3	1360388	1360757	FvH4_3g02671	gene19547	1			
gene19546	Fvb3:1365621-1367459	Fvb3	1365621	1367459	FvH4_3g02680	gene19546	0.26	GO:0005524,(	ATRP1,RP1	PPDK regulatory protein
gene19545	Fvb3:1368552-1371552	Fvb3	1368552	1371552	FvH4_3g02690	gene19545	0.16			
gene19544	Fvb3:1378211-1380130	Fvb3	1378211	1380130	FvH4_3g02700	gene19544	0.18	GO:0004601,GO:0006979,GO:00200		Peroxidase superfamily protein
gene39839	Fvb3:1380840-1381773	Fvb3	1380840	1381773	FvH4_3g02710	gene39839	1			Late embryogenesis abundant (LEA) hydroxyproline-rich glycoprotein family
gene19543	Fvb3:1383480-1384064	Fvb3	1383480	1384064	FvH4_3g02720	gene19543	1		NDR1	Late embryogenesis abundant (LEA) hydroxyproline-rich glycoprotein family
gene19542	Fvb3:1384043-1386849	Fvb3	1384043	1386849	FvH4_3g02730	gene19542	0.35	GO:0009245,GO:0016410		Trimeric LpxA-like enzymes superfamily protein
gene19541	Fvb3:1386762-1390102	Fvb3	1386762	1390102	FvH4_3g02731	gene19541	0.47	GO:0004672,(	CRK25	cysteine-rich RLK (RECEPTOR-like protein kinase) 25
gene36095	Fvb3:1390832-1393829	Fvb3	1390832	1393829	FvH4_3g02732	gene36095	0.47	GO:0004672,(	CRK25	cysteine-rich RLK (RECEPTOR-like protein kinase) 25
gene39840	Fvb3:1393775-1394278	Fvb3	1393775	1394278	FvH4_3g02750	gene39840	0.23			Trimeric LpxA-like enzymes superfamily protein
gene36096	Fvb3:1394904-1397882	Fvb3	1394904	1397882	FvH4_3g02751	gene36096	0.47	GO:0004672,(	CRK25	cysteine-rich RLK (RECEPTOR-like protein kinase) 25
gene19539	Fvb3:1398823-1401464	Fvb3	1398823	1401464	FvH4_3g02752	gene19539	0.48	GO:0004672,(	CRK25	cysteine-rich RLK (RECEPTOR-like protein kinase) 25
gene19538	Fvb3:1406724-1407994	Fvb3	1406724	1407994	FvH4_3g02753	gene19538	1			
gene19537	Fvb3:1418298-1425301	Fvb3	1418298	1425301	FvH4_3g02754	gene19537	0.47	GO:0004672,(	CRK25	cysteine-rich RLK (RECEPTOR-like protein kinase) 25
gene19534	Fvb3:1426375-1430307	Fvb3	1426375	1430307	FvH4_3g02770	gene19534	0.47	GO:0004672,(	CRK25	cysteine-rich RLK (RECEPTOR-like protein kinase) 25
gene19533	Fvb3:1431110-1435446	Fvb3	1431110	1435446	FvH4_3g02771	gene19533	0.45	GO:0004672,(	CRK10,RLK4	cysteine-rich RLK (RECEPTOR-like protein kinase) 10
gene19532	Fvb3:1437798-1443554	Fvb3	1437798	1443554	FvH4_3g02772	gene19532	0.46	GO:0004672,(	CRK10,RLK4	cysteine-rich RLK (RECEPTOR-like protein kinase) 10
gene19530	Fvb3:1449956-1453571	Fvb3	1449956	1453571	FvH4_3g02773	gene19530	0.51	GO:0004672,(	CRK25	cysteine-rich RLK (RECEPTOR-like protein kinase) 25
gene19528	Fvb3:1456071-1458661	Fvb3	1456071	1458661	FvH4_3g02774	gene19528	0.47	GO:0004672,(	CRK25	cysteine-rich RLK (RECEPTOR-like protein kinase) 25
gene19527	Fvb3:1465643-1466101	Fvb3	1465643	1466101	FvH4_3g02790	gene19527	1	GO:0006855,GO:0015238,GO:00152		MATE efflux family protein
gene39841	Fvb3:1471384-1473237	Fvb3	1471384	1473237	FvH4_3g02800	gene39841	0.44	GO:0004672,(	CRK25	cysteine-rich RLK (RECEPTOR-like protein kinase) 25
gene19526	Fvb3:1473385-1473941	Fvb3	1473385	1473941	FvH4_3g02801	gene19526	0.48	GO:0004672,(	CRK33	cysteine-rich RLK (RECEPTOR-like protein kinase) 33
gene19525	Fvb3:1475940-1479008	Fvb3	1475940	1479008	FvH4_3g02820	gene19525	0.41	GO:0004672,(	CRK25	cysteine-rich RLK (RECEPTOR-like protein kinase) 25
gene19524	Fvb3:1479970-1482127	Fvb3	1479970	1482127	FvH4_3g02830	gene19524	0.57	GO:0004672,(	CRK25	cysteine-rich RLK (RECEPTOR-like protein kinase) 25
gene19523	Fvb3:1482849-1484843	Fvb3	1482849	1484843	FvH4_3g02831	gene19523	1	GO:0006855,GO:0015238,GO:00152		MATE efflux family protein
gene36098	Fvb3:1488257-1490637	Fvb3	1488257	1490637	FvH4_3g02832	gene36098	0.45		CRK25	cysteine-rich RLK (RECEPTOR-like protein kinase) 25
gene36099	Fvb3:1491475-1492987	Fvb3	1491475	1492987	FvH4_3g02833	gene36099	0.53		CRK29	cysteine-rich RLK (RECEPTOR-like protein kinase) 29
gene36100	Fvb3:1494127-1497293	Fvb3	1494127	1497293	FvH4_3g02834	gene36100	0.42	GO:0004672,(	CRK29	cysteine-rich RLK (RECEPTOR-like protein kinase) 29
gene36103	Fvb3:1498681-1511405	Fvb3	1498681	1511405	FvH4_3g02850	gene36103	0.39		ATRPA70C,RPA70C	Replication factor-A protein 1-related
gene19521	Fvb3:1512633-1513880	Fvb3	1512633	1513880	FvH4_3g02851	gene19521	1	GO:0003676		
gene24648	Fvb3:1528115-1528692	Fvb3	1528115	1528692	FvH4_3g02852	gene24648	1			
gene24649	Fvb3:1531980-1534897	Fvb3	1531980	1534897	FvH4_3g02870	gene24649	0.12	GO:0004672,(	CRK29	cysteine-rich RLK (RECEPTOR-like protein kinase) 29
gene36104	Fvb3:1535226-1536646	Fvb3	1535226	1536646	FvH4_3g02871	gene36104	0			
gene24650	Fvb3:1536971-1537610	Fvb3	1536971	1537610	FvH4_3g02880	gene24650	1	GO:0003677,(	AGL29	AGAMOUS-like 29
gene24651	Fvb3:1537709-1539815	Fvb3	1537709	1539815	FvH4_3g02881	gene24651	1			
gene39842	Fvb3:1540876-1541927	Fvb3	1540876	1541927	FvH4_3g02882	gene39842	1		ATK5	kinesin 5
gene24652	Fvb3:1542978-1545247	Fvb3	1542978	1545247	FvH4_3g02890	gene24652	0.01			RNI-like superfamily protein
gene24653	Fvb3:1546731-1549678	Fvb3	1546731	1549678	FvH4_3g02900	gene24653	0.04	GO:0016021		Sulfite exporter TauE/SafE family protein
gene24654	Fvb3:1549701-1552477	Fvb3	1549701	1552477	FvH4_3g02910	gene24654	0.16	GO:0046983	bHLH105,ILR3	basic helix-loop-helix (bHLH) DNA-binding superfamily protein
gene24655	Fvb3:1553281-1554668	Fvb3	1553281	1554668	FvH4_3g02911	gene24655	0.04	GO:0006662,(	ATCXXS1,CXXS1	C-terminal cysteine residue is changed to a serine 1
gene24656	Fvb3:1554833-1559824	Fvb3	1554833	1559824	FvH4_3g02912	gene24656	0.09	GO:0003777,(	ATK1,KATA,KATAP	kinesin 1
gene24657	Fvb3:1561438-1562904	Fvb3	1561438	1562904	FvH4_3g02920	gene24657	0.17	GO:0005509,(	PSBQ,PSBQ-2,PSII-Q	photosystem II subunit Q-2
gene24658	Fvb3:1564146-1567056	Fvb3	1564146	1567056	FvH4_3g02930	gene24658	1	GO:0006355		DNA-binding storekeeper protein-related transcriptional regulator
gene24659	Fvb3:1569121-1570130	Fvb3	1569121	1570130	FvH4_3g02940	gene24659	0.15	GO:0000786,(	HTA2	histone H2A 2
gene24660	Fvb3:1569878-1570816	Fvb3	1569878	1570816	FvH4_3g02950	gene24660	0.03			Protein of unknown function (DUF1218)
gene24661	Fvb3:1571146-1573912	Fvb3	1571146	1573912	FvH4_3g02960	gene24661	0.19	GO:0003824	HSA32	Aldolase-type TIM barrel family protein
gene24663	Fvb3:1576006-1578248	Fvb3	1576006	1578248	FvH4_3g02970	gene24663	0.36			
gene24664	Fvb3:1578846-1582265	Fvb3	1578846	1582265	FvH4_3g02971	gene24664	1			
gene24665	Fvb3:1583755-1586819	Fvb3	1583755	1586819	FvH4_3g02980	gene24665	0.21	GO:0003824,(	BAN	NAD(P)-binding Rossmann-fold superfamily protein
gene24666	Fvb3:1585474-1588821	Fvb3	1585474	1588821	FvH4_3g02990	gene24666	0.06	GO:0004672,(	CRLK1	Protein kinase superfamily protein
gene24667	Fvb3:1590747-1593542	Fvb3	1590747	1593542	FvH4_3g03000	gene24667	0.12	GO:0000287,(	ATTPS03,TPS03	terpene synthase 03
gene24668	Fvb3:1594603-1595709	Fvb3	1594603	1595709	FvH4_3g03010	gene24668	0.27	GO:0004842,GO:0016567		RING/U-box superfamily protein
gene24669	Fvb3:1599666-1604244	Fvb3	1599666	1604244	FvH4_3g03030	gene24669	0.18	GO:0003743		eukaryotic translation initiation factor-related
gene24670	Fvb3:1604517-1607349	Fvb3	1604517	1607349	FvH4_3g03040	gene24670	0	GO:0005515		Phototropic-responsive NPH3 family protein
gene24671	Fvb3:1607881-1612771	Fvb3	1607881	1612771	FvH4_3g03041	gene24671	0.04	GO:0005515,GO:0046872		phosphoinositide binding
gene24672	Fvb3:1613290-1615710	Fvb3	1613290	1615710	FvH4_3g03042	gene24672	0.43	GO:0000287,(	ATTPS14,TPS14	terpene synthase 14
gene34030	Fvb3:1615982-1617349	Fvb3	1615982	1617349	FvH4_3g03043	gene34030	1			Tetratricopeptide repeat (TPR)-like superfamily protein
gene36105	Fvb3:1623630-1624412	Fvb3	1623630	1624412	FvH4_3g03044	gene36105	0.66			
gene39843	Fvb3:1624708-1629243	Fvb3	1624708	1629243	FvH4_3g03045	gene39843	0.44	GO:0000287,(	ATTPS14,TPS14	terpene synthase 14
gene24675	Fvb3:1629270-1629924	Fvb3	1629270	1629924	FvH4_3g03060	gene24675	0.46	GO:0005515		Tetratricopeptide repeat (TPR)-like superfamily protein
gene24676	Fvb3:1633206-1635362	Fvb3	1633206	1635362	FvH4_3g03061	gene24676	0.44	GO:0000287,(	ATTPS14,TPS14	terpene synthase 14
gene24677	Fvb3:1635670-1636316	Fvb3	1635670	1636316	FvH4_3g03070	gene24677	0.41	GO:0005515		Tetratricopeptide repeat (TPR)-like superfamily protein
gene24678	Fvb3:1636706-1637892	Fvb3	1636706	1637892	FvH4_3g03071	gene24678	0.66			phosphoinositide binding
gene39844	Fvb3:1642571-1645757	Fvb3	1642571	1645757	FvH4_3g03072	gene39844	0.6	GO:0000287,(	ATTPS14,TPS14	terpene synthase 14
gene24673	Fvb3:1645119-1647527	Fvb3	1645119	1647527	FvH4_3g03080	gene24673	0.37	GO:0005515		Tetratricopeptide repeat (TPR)-like superfamily protein
gene39845	Fvb3:1649467-1654658	Fvb3	1649467	1654658	FvH4_3g03081	gene39845	0.42	GO:0000287,(	ATTPS14,TPS14	terpene synthase 14
gene39846	Fvb3:1654662-1655297	Fvb3	1654662	1655297	FvH4_3g03090	gene39846	0.45	GO:0005515		Tetratricopeptide repeat (TPR)-like superfamily protein
gene39847	Fvb3:1657959-1658828	Fvb3	1657959	1658828	FvH4_3g03100	gene39847	1	GO:0009055,GO:0015035,GO:00454		Glutaredoxin family protein
gene36106	Fvb3:1664540-1666004	Fvb3	1664540	1666004	FvH4_3g03101	gene36106	1	GO:0003677,GO:0046983		BED zinc finger ;hAT family dimerisation domain
gene39848	Fvb3:1673389-1676118	Fvb3	1673389	1676118	FvH4_3g03110	gene39848	0.44			
gene36107	Fvb3:1677359-1678352	Fvb3	1677359	1678352	FvH4_3g03120	gene36107	1			
gene30670	Fvb3:1678430-1680235	Fvb3	1678430	1680235	FvH4_3g03130	gene30670	0	GO:0008168	ATBSMT1,BSMT1	S-adenosyl-L-methionine-dependent methyltransferases superfamily protein
gene30669	Fvb3:1680583-1685225	Fvb3	1680583	1685225	FvH4_3g03150	gene30669	0.2	GO:0000287,(	ATTPS14,TPS14	terpene synthase 14
gene30668	Fvb3:1685456-1686592	Fvb3	1685456	1686592	FvH4_3g03160	gene30668	0	GO:0005515		Tetratricopeptide repeat (TPR)-like superfamily protein
gene30667	Fvb3:1687520-1688818	Fvb3	1687520	1688818	FvH4_3g03170	gene30667	0.37	GO:0046983	DYT1	basic helix-loop-helix (bHLH) DNA-binding superfamily protein
gene36108	Fvb3:1690369-1690947	Fvb3	1690369	1690947	FvH4_3g03180	gene36108	0.32			Protein of unknown function, DUF538
gene36109	Fvb3:1691861-1692829	Fvb3	1691861	1692829	FvH4_3g03190	gene36109	1	GO:0070300		
gene30665	Fvb3:1701110-1704158	Fvb3	1701110	1704158	FvH4_3g03200	gene30665	0.09	GO:0046983		basic helix-loop-helix (bHLH) DNA-binding superfamily protein
gene30664	Fvb3:1707024-1710101	Fvb3	1707024	1710101	FvH4_3g03210	gene30664	0.21	GO:0003955,(	FQR1	flavodoxin-like quinone reductase 1
gene30663	Fvb3:1712123-1715078	Fvb3	1712123	1715078	FvH4_3g03220	gene30663	0.08	GO:0004842,(	B80,PUB8	plant U-box 8
gene30662	Fvb3:1717851-1721952	Fvb3	1717851	1721952	FvH4_3g03230	gene30662	0.43	GO:0004674,GO:0005524,GO:00064		S-locus lectin protein kinase family protein
gene30661	Fvb3:1729201-1732990	Fvb3	1729201	1732990	FvH4_3g03231	gene30661	0.42	GO:0004674,GO:0005524,GO:00064		S-locus lectin protein kinase family protein
gene30660	Fvb3:1734251-1737670	Fvb3	1734251	1737670	FvH4_3g03240	gene30660	0.44	GO:0004674,GO:0005524,GO:00064		S-locus lectin protein kinase family protein
gene30659	Fvb3:1740132-1743982	Fvb3	1740132	1743982	FvH4_3g03241	gene30659	0.44	GO:0004674,GO:0005524,GO:00064		S-locus lectin protein kinase family protein
gene30658	Fvb3:1746423-1750099	Fvb3	1746423	1750099	FvH4_3g03242	gene30658	0.44	GO:0004674,GO:0005524,GO:00064		S-locus lectin protein kinase family protein
gene30657	Fvb3:1754164-1757586	Fvb3	1754164	1757586	FvH4_3g03243	gene30657	0.48	GO:0004674,GO:0005524,GO:00064		S-locus lectin protein kinase family protein
gene30656	Fvb3:1757967-1760459	Fvb3	1757967	1760459	FvH4_3g03250	gene30656	1	GO:0005515,GO:0006396		crooked neck protein, putative / cell cycle protein, putative
gene30655	Fvb3:1762502-1763232	Fvb3	1762502	1763232	FvH4_3g03251	gene30655	0.35			
gene30654	Fvb3:1764174-1766346	Fvb3	1764174	1766346	FvH4_3g03260	gene30654	0.14	GO:0009690,(	ATCKX6,ATCKX7,CKX6	cytokinin oxidase/dehydrogenase 6
gene30653	Fvb3:1768450-1769979	Fvb3	1768450	1769979	FvH4_3g03280	gene30653	0.13	GO:0005524,(	ATRLI2,RLI2	RNAse l inhibitor protein 2
gene30652	Fvb3:1771083-1774428	Fvb3	1771083	1774428	FvH4_3g03300	gene30652	0.38	GO:0004674,GO:0005524,GO:00064		S-locus lectin protein kinase family protein
gene30651	Fvb3:1775603-1779237	Fvb3	1775603	1779237	FvH4_3g03301	gene30651	0.36	GO:0004674,GO:0005524,GO:00064		S-locus lectin protein kinase family protein
gene30650	Fvb3:1779994-1783527	Fvb3	1779994	1783527	FvH4_3g03310	gene30650	0.37	GO:0004674,GO:0005524,GO:00064		S-locus lectin protein kinase family protein
gene30649	Fvb3:1786992-1790349	Fvb3	1786992	1790349	FvH4_3g03320	gene30649	0.09	GO:0004674,GO:0005524,GO:00064		S-locus lectin protein kinase family protein

gene39849	Fvb3:1796903-1797935	Fvb3	1796903	1797935	FvH4_3g03321	gene39849	1			
gene39850	Fvb3:1802085-1802574	Fvb3	1802085	1802574	FvH4_3g03322	gene39850	0.47		B120	S-locus lectin protein kinase family protein
gene39851	Fvb3:1802838-1805248	Fvb3	1802838	1805248	FvH4_3g03323	gene39851	0.35	GO:0004672,GO:0005524,GO:00064		S-locus lectin protein kinase family protein
gene30646	Fvb3:1805959-1806562	Fvb3	1805959	1806562	FvH4_3g03324	gene30646	0.45			
gene30645	Fvb3:1812569-1815462	Fvb3	1812569	1815462	FvH4_3g03325	gene30645	1			
gene34404	Fvb3:1815809-1816872	Fvb3	1815809	1816872	FvH4_3g03340	gene34404	0.58	GO:0048544		S-locus lectin protein kinase family protein
gene30644	Fvb3:1818089-1821332	Fvb3	1818089	1821332	FvH4_3g03350	gene30644	0.41	GO:0004674,GO:0005524,GO:00064		S-locus lectin protein kinase family protein
gene39852	Fvb3:1821652-1821882	Fvb3	1821652	1821882	FvH4_3g03351	gene39852	0.49			S-locus lectin protein kinase family protein
gene39853	Fvb3:1821888-1822607	Fvb3	1821888	1822607	FvH4_3g03360	gene39853	0.47			
gene30643	Fvb3:1829057-1832851	Fvb3	1829057	1832851	FvH4_3g03370	gene30643	0.05	GO:0004674,GO:0005524,GO:00064		S-locus lectin protein kinase family protein
gene30642	Fvb3:1832450-1838776	Fvb3	1832450	1838776	FvH4_3g03390	gene30642	0.35	GO:0004674,GO:0005524,GO:00064		S-locus lectin protein kinase family protein
gene30641	Fvb3:1838900-1842240	Fvb3	1838900	1842240	FvH4_3g03400	gene30641	0.08			lysine decarboxylase family protein
gene30640	Fvb3:1842526-1847095	Fvb3	1842526	1847095	FvH4_3g03410	gene30640	0.37	GO:0004674,GO:0005524,GO:00064		S-locus lectin protein kinase family protein
gene30639	Fvb3:1846715-1850040	Fvb3	1846715	1850040	FvH4_3g03420	gene30639	0.51	GO:0004674,(	ARK3,RK3	receptor kinase 3
gene30638	Fvb3:1850406-1855533	Fvb3	1850406	1855533	FvH4_3g03430	gene30638	0.47	GO:0004674,(	ARK3,RK3	receptor kinase 3
gene30637	Fvb3:1857375-1862568	Fvb3	1857375	1862568	FvH4_3g03431	gene30637	0.53	GO:0004674,GO:0005524,GO:00064		S-locus lectin protein kinase family protein
gene30636	Fvb3:1864042-1867792	Fvb3	1864042	1867792	FvH4_3g03432	gene30636	0.56	GO:0004674,GO:0005524,GO:00064		S-locus lectin protein kinase family protein
gene30635	Fvb3:1868486-1872250	Fvb3	1868486	1872250	FvH4_3g03433	gene30635	0.54	GO:0004674,(	SD1-29	S-domain-1 29
gene39854	Fvb3:1872986-1875693	Fvb3	1872986	1875693	FvH4_3g03434	gene39854	0.48	GO:0004672,GO:0005524,GO:00064		S-locus lectin protein kinase family protein
gene39855	Fvb3:1875960-1876356	Fvb3	1875960	1876356	FvH4_3g03435	gene39855	0.5		SD1-29	S-domain-1 29
gene39856	Fvb3:1879585-1883401	Fvb3	1879585	1883401	FvH4_3g03450	gene39856	0.47	GO:0004674,(	SD1-29	S-domain-1 29
gene30633	Fvb3:1885337-1888514	Fvb3	1885337	1888514	FvH4_3g03451	gene30633	0.47	GO:0004672,GO:0005524,GO:00064		S-locus lectin protein kinase family protein
gene39857	Fvb3:1890169-1891176	Fvb3	1890169	1891176	FvH4_3g03460	gene39857	0.5	GO:0004672,GO:0006468		S-locus lectin protein kinase family protein
gene39858	Fvb3:1891752-1895554	Fvb3	1891752	1895554	FvH4_3g03461	gene39858	0.47	GO:0004674,(	SD1-29	S-domain-1 29
gene30630	Fvb3:1900765-1902550	Fvb3	1900765	1902550	FvH4_3g03480	gene30630	0.1	GO:0003824,(	ACS8	1-amino-cyclopropane-1-carboxylate synthase 8
gene39859	Fvb3:1903504-1909697	Fvb3	1903504	1909697	FvH4_3g03481	gene39859	0.5	GO:0004674,GO:0005524,GO:00064		S-locus lectin protein kinase family protein
gene30631	Fvb3:1910643-1914082	Fvb3	1910643	1914082	FvH4_3g03482	gene30631	0.46	GO:0004674,GO:0005524,GO:00064		S-locus lectin protein kinase family protein
gene39860	Fvb3:1925559-1926137	Fvb3	1925559	1926137	FvH4_3g03500	gene39860	1			
gene39861	Fvb3:1926399-1930238	Fvb3	1926399	1930238	FvH4_3g03501	gene39861	0.52	GO:0004674,(	SD1-29	S-domain-1 29
gene39862	Fvb3:1931602-1934547	Fvb3	1931602	1934547	FvH4_3g03502	gene39862	0.44	GO:0004672,GO:0005524,GO:00064		S-locus lectin protein kinase family protein
gene30752	Fvb3:1937726-1941392	Fvb3	1937726	1941392	FvH4_3g03520	gene30752	0.42	GO:0004674,(	SD1-29	S-domain-1 29
gene30751	Fvb3:1947703-1951778	Fvb3	1947703	1951778	FvH4_3g03521	gene30751	0.49	GO:0004674,(	SD1-29	S-domain-1 29
gene30750	Fvb3:1953397-1955959	Fvb3	1953397	1955959	FvH4_3g03530	gene30750	0.17	GO:0003677,(	LFY,LFY3	floral meristem identity control protein LEAFY (LFY)
gene30749	Fvb3:1957303-1959663	Fvb3	1957303	1959663	FvH4_3g03540	gene30749	0.51	GO:0003677,(	ANAC098,ATCUC2,CU	NAC (No Apical Meristem) domain transcriptional regulator superfamily protein
gene39863	Fvb3:1968041-1969872	Fvb3	1968041	1969872	FvH4_3g03541	gene39863	0.47	GO:0004672,GO:0005524,GO:00064		S-locus protein kinase, putative
gene39864	Fvb3:1971043-1972422	Fvb3	1971043	1972422	FvH4_3g03542	gene39864	1			Gag-Pol-related retrotransposon family protein
gene39865	Fvb3:1972940-1973908	Fvb3	1972940	1973908	FvH4_3g03543	gene39865	1			Reverse transcriptase (RNA-dependent DNA polymerase)
gene30748	Fvb3:1977342-1981619	Fvb3	1977342	1981619	FvH4_3g03560	gene30748	0.39	GO:0004674,(	SD1-29	S-domain-1 29
gene36110	Fvb3:1982582-1984962	Fvb3	1982582	1984962	FvH4_3g03561	gene36110	1		CRK8	cysteine-rich RLK (RECEPTOR-like protein kinase) 8
gene30746	Fvb3:1993305-1994378	Fvb3	1993305	1994378	FvH4_3g03580	gene30746	1		ANAC100,ATNAC5,NA	NAC domain containing protein 100
gene30745	Fvb3:2002057-2004128	Fvb3	2002057	2004128	FvH4_3g03581	gene30745	0.44	GO:0048544	SD1-29	S-domain-1 29
gene30744	Fvb3:2005205-2010120	Fvb3	2005205	2010120	FvH4_3g03590	gene30744	0.25	GO:0004674,(	B120	S-locus lectin protein kinase family protein
gene30743	Fvb3:2012052-2012596	Fvb3	2012052	2012596	FvH4_3g03600	gene30743	0.15	GO:0005509		Calcium-binding EF-hand family protein
gene39866	Fvb3:2017693-2018750	Fvb3	2017693	2018750	FvH4_3g03601	gene39866	1			
gene36111	Fvb3:2025184-2026088	Fvb3	2025184	2026088	FvH4_3g03610	gene36111	0	GO:0005509		Calcium-binding EF-hand family protein
gene36112	Fvb3:2032026-2041011	Fvb3	2032026	2041011	FvH4_3g03620	gene36112	0.12	GO:0000155,(	AHK2,HK2	histidine kinase 2
gene30741	Fvb3:2041916-2043479	Fvb3	2041916	2043479	FvH4_3g03630	gene30741	0.37	GO:0000977,(	AGL24	AGAMOUS-like 24
gene30740	Fvb3:2045154-2047400	Fvb3	2045154	2047400	FvH4_3g03640	gene30740	0	GO:0005622,GO:0008270		B-box type zinc finger family protein
gene30739	Fvb3:2047356-2051306	Fvb3	2047356	2051306	FvH4_3g03650	gene30739	0.16	GO:0006950		Adenine nucleotide alpha hydrolases-like superfamily protein
gene36113	Fvb3:2051965-2054538	Fvb3	2051965	2054538	FvH4_3g03660	gene36113	0	GO:0061630		phosphoinositide binding
gene30737	Fvb3:2055536-2058092	Fvb3	2055536	2058092	FvH4_3g03670	gene30737	0.15			PLAC8 family protein
gene30736	Fvb3:2059475-2061075	Fvb3	2059475	2061075	FvH4_3g03680	gene30736	0.07	GO:0003677	ATMYB58,MYB58	myb domain protein 58
gene30735	Fvb3:2063791-2067097	Fvb3	2063791	2067097	FvH4_3g03690	gene30735	0.01			Protein of unknown function (DUF594)
gene30734	Fvb3:2067408-2069341	Fvb3	2067408	2069341	FvH4_3g03700	gene30734	0.26	GO:0005615		Serine protease inhibitor (SERPIN) family protein
gene30732	Fvb3:2075516-2077533	Fvb3	2075516	2077533	FvH4_3g03710	gene30732	0.25	GO:0005615		Serine protease inhibitor (SERPIN) family protein
gene30731	Fvb3:2079582-2081684	Fvb3	2079582	2081684	FvH4_3g03720	gene30731	0.04	GO:0005634,(	CYCD6;1	Cyclin D6;1
gene30730	Fvb3:2087041-2089912	Fvb3	2087041	2089912	FvH4_3g03730	gene30730	1	GO:0005515		Octicosapeptide/Phox/Bem1p family protein
gene30729	Fvb3:2093477-2096784	Fvb3	2093477	2096784	FvH4_3g03740	gene30729	0.36	GO:0005337,(	ATENT3,ENT3,FUR1	Major facilitator superfamily protein
gene30728	Fvb3:2097824-2100941	Fvb3	2097824	2100941	FvH4_3g03750	gene30728	0.34	GO:0005337,(	ATENT3,ENT3,FUR1	Major facilitator superfamily protein
gene39867	Fvb3:2101296-2102656	Fvb3	2101296	2102656	FvH4_3g03751	gene39867	1	GO:0003676,GO:0004523		Polynucleotidyl transferase, ribonuclease H-like superfamily protein
gene30727	Fvb3:2108104-2114350	Fvb3	2108104	2114350	FvH4_3g03760	gene30727	0.03		B160	Zinc finger, RING-type;Transcription factor jumonji/aspartyl beta-hydroxylase
gene30726	Fvb3:2114368-2118237	Fvb3	2114368	2118237	FvH4_3g03770	gene30726	0.12			Protein kinase superfamily protein
gene30725	Fvb3:2123941-2125668	Fvb3	2123941	2125668	FvH4_3g03780	gene30725	0	GO:0003677	ATM4,ATMYB102,MYI	MYB-like 102
gene30724	Fvb3:2130409-2133928	Fvb3	2130409	2133928	FvH4_3g03790	gene30724	0.14	GO:0046854		Inositol monophosphatase family protein
gene36114	Fvb3:2134381-2135089	Fvb3	2134381	2135089	FvH4_3g03800	gene36114	0			
gene30723	Fvb3:2136348-2137154	Fvb3	2136348	2137154	FvH4_3g03810	gene30723	1			Wound-responsive family protein
gene39868	Fvb3:2138534-2139174	Fvb3	2138534	2139174	FvH4_3g03820	gene39868	1			
gene30722	Fvb3:2139906-2141834	Fvb3	2139906	2141834	FvH4_3g03821	gene30722	1			
gene30720	Fvb3:2148137-2148516	Fvb3	2148137	2148516	FvH4_3g03840	gene30720	1	GO:0009116		uridine 5'-monophosphate synthase / UMP synthase (PYRE-F) (UMPS)
gene30719	Fvb3:2155134-2156798	Fvb3	2155134	2156798	FvH4_3g03870	gene30719	0			
gene30718	Fvb3:2158074-2161938	Fvb3	2158074	2161938	FvH4_3g03880	gene30718	0.2	GO:0005789		PapD-like superfamily protein
gene30717	Fvb3:2164174-2164943	Fvb3	2164174	2164943	FvH4_3g03881	gene30717	1			
gene30716	Fvb3:2166172-2166854	Fvb3	2166172	2166854	FvH4_3g03882	gene30716	1			
gene30715	Fvb3:2172524-2175538	Fvb3	2172524	2175538	FvH4_3g03900	gene30715	0.08	GO:0016021,(	ATSTP1,STP1	sugar transporter 1
gene30714	Fvb3:2180550-2183354	Fvb3	2180550	2183354	FvH4_3g03910	gene30714	1	GO:0005515,GO:0006396		Tetratricopeptide repeat (TPR)-like superfamily protein
gene30713	Fvb3:2183855-2184805	Fvb3	2183855	2184805	FvH4_3g03920	gene30713	1			
gene30712	Fvb3:2185797-2187518	Fvb3	2185797	2187518	FvH4_3g03930	gene30712	1	GO:0005515		ARM repeat superfamily protein
gene30711	Fvb3:2192859-2197338	Fvb3	2192859	2197338	FvH4_3g03931	gene30711	1			
gene30710	Fvb3:2197836-2201324	Fvb3	2197836	2201324	FvH4_3g03932	gene30710	1			zinc knuckle (CCHC-type) family protein
gene30709	Fvb3:2202893-2205067	Fvb3	2202893	2205067	FvH4_3g03940	gene30709	0	GO:0005515		F-box family protein
gene30708	Fvb3:2207665-2208496	Fvb3	2207665	2208496	FvH4_3g03950	gene30708	0.13	GO:0007017,GO:0030286		Dynein light chain type 1 family protein
gene30707	Fvb3:2209479-2214082	Fvb3	2209479	2214082	FvH4_3g03960	gene30707	0.09	GO:0005509,(	NDB2	NAD(P)H dehydrogenase B2
gene30706	Fvb3:2217498-2218328	Fvb3	2217498	2218328	FvH4_3g03970	gene30706	1			
gene30705	Fvb3:2220247-2223648	Fvb3	2220247	2223648	FvH4_3g03980	gene30705	0.02	GO:0005515		
gene30704	Fvb3:2224636-2225868	Fvb3	2224636	2225868	FvH4_3g03990	gene30704	0.01	GO:0005515	UFO	F-box family protein
gene30703	Fvb3:2228125-2230499	Fvb3	2228125	2230499	FvH4_3g04000	gene30703	0.11			SGNH hydrolase-type esterase superfamily protein
gene30702	Fvb3:2233254-2235273	Fvb3	2233254	2235273	FvH4_3g04010	gene30702	0.16		DFL1,GH3.6	Auxin-responsive GH3 family protein
gene30701	Fvb3:2238784-2240383	Fvb3	2238784	2240383	FvH4_3g04011	gene30701	1			
gene30700	Fvb3:2241443-2244542	Fvb3	2241443	2244542	FvH4_3g04012	gene30700	1		ARP2,ATARP2,WRM	actin related protein 2
gene30699	Fvb3:2245250-2246484	Fvb3	2245250	2246484	FvH4_3g04020	gene30699	0.02	GO:0005515		F-box family protein
gene30698	Fvb3:2248238-2251991	Fvb3	2248238	2251991	FvH4_3g04030	gene30698	0.07	GO:0005515		Transducin/WD40 repeat-like superfamily protein
gene30697	Fvb3:2252416-2252727	Fvb3	2252416	2252727	FvH4_3g04031	gene30697	1			
gene30696	Fvb3:2254908-2260020	Fvb3	2254908	2260020	FvH4_3g04040	gene30696	0.22	GO:0008531,(	ATFMN/FHY,FMN/FH'	riboflavin kinase/FMN hydrolase
gene30695	Fvb3:2260488-2269210	Fvb3	2260488	2269210	FvH4_3g04050	gene30695	0.14	GO:0005515,GO:0005680,GO:00300		Transducin/WD40 repeat-like superfamily protein
gene30694	Fvb3:2267614-2272975	Fvb3	2267614	2272975	FvH4_3g04060	gene30694	0.11	GO:0005516,GO:0006950		Calmodulin-binding protein
gene30693	Fvb3:2273592-2276556	Fvb3	2273592	2276556	FvH4_3g04070	gene30693	0.21			
gene30692	Fvb3:2279559-2283961	Fvb3	2279559	2283961	FvH4_3g04090	gene30692	0.15	GO:0003951,(	SPHK1	sphingosine kinase 1
gene30691	Fvb3:2288504-2292230	Fvb3	2288504	2292230	FvH4_3g04100	gene30691	0.16	GO:0003824,GO:0005992		Haloacid dehalogenase-like hydrolase (HAD) superfamily protein
gene36115	Fvb3:2296892-2298138	Fvb3	2296892	2298138	FvH4_3g04110	gene36115	0.13			
gene36116	Fvb3:2303454-2311128	Fvb3	2303454	2311128	FvH4_3g04120	gene36116	0.08	GO:0003677,(	HSI2-L1,HSL1,VAL2	HSI2-like 1
gene30689	Fvb3:2311925-2313808	Fvb3	2311925	2313808	FvH4_3g04130	gene30689	0.18	GO:0005484,(	ATSYP124,SYP124	syntaxin of plants 124
gene30688	Fvb3:2313986-2316242	Fvb3	2313986	2316242	FvH4_3g04140	gene30688	0.03	GO:0000813,(	VPS28-1,VPS28-2	Vacuolar protein sorting-associated protein VPS28 family protein
gene30687	Fvb3:2316784-2321613	Fvb3	2316784	2321613	FvH4_3g04150	gene30687	0.04	GO:0005515,GO:0006396		Tetratricopeptide repeat (TPR)-like superfamily protein
gene30685	Fvb3:2322710-2324665	Fvb3	2322710	2324665	FvH4_3g04160	gene30685	0.01			Protein of unknown function (DUF761)
gene30684	Fvb3:2330051-2330996	Fvb3	2330051	2330996	FvH4_3g04161	gene30684	1			
gene30683	Fvb3:2331651-2335381	Fvb3	2331651	2335381	FvH4_3g04170	gene30683	0.03	GO:0003677,(	LFY,LFY3	floral meristem identity control protein LEAFY (LFY)
gene30682	Fvb3:2337237-2339099	Fvb3	2337237	2339099	FvH4_3g04180	gene30682	0.12	GO:0003824,(	ACS8	1-amino-cyclopropane-1-carboxylate synthase 8
gene39869	Fvb3:2339390-2340260	Fvb3	2339390	2340260	FvH4_3g04190	gene39869	0.22	GO:0003735,GO:0005840,GO:00064		Ribosomal protein L11 family protein
gene36117	Fvb3:2345654-2347251	Fvb3	2345654	2347251	FvH4_3g04191	gene36117	1			

gene30680	Fvb3:2352139-2358276	Fvb3	2352139	2358276	FvH4_3g04200	gene30680	0.24		ATSNF4,SNF4	homolog of yeast sucrose nonfermenting 4
gene39870	Fvb3:2359667-2360037	Fvb3	2359667	2360037	FvH4_3g04210	gene39870	1		DVL18,RTFL5	ROTUNDIFOLIA like 5
gene30679	Fvb3:2361295-2365490	Fvb3	2361295	2365490	FvH4_3g04220	gene30679	0.18	GO:0005096	AGD5,NEV	ARF-GAP domain 5
gene39871	Fvb3:2366518-2367865	Fvb3	2366518	2367865	FvH4_3g04230	gene39871	0.44			
gene39872	Fvb3:2368453-2369188	Fvb3	2368453	2369188	FvH4_3g04231	gene39872	0.44			Protein of unknown function (DUF300)
gene30678	Fvb3:2369280-2370216	Fvb3	2369280	2370216	FvH4_3g04232	gene30678	0.37			Protein of unknown function (DUF300)
gene30677	Fvb3:2372687-2378024	Fvb3	2372687	2378024	FvH4_3g04233	gene30677	1			
gene30676	Fvb3:2386233-2387546	Fvb3	2386233	2387546	FvH4_3g04240	gene30676	0.44			
gene30675	Fvb3:2388143-2389916	Fvb3	2388143	2389916	FvH4_3g04241	gene30675	0.29			Protein of unknown function (DUF300)
gene30674	Fvb3:2392811-2393323	Fvb3	2392811	2393323	FvH4_3g04242	gene30674	0.8			
gene30673	Fvb3:2394024-2395850	Fvb3	2394024	2395850	FvH4_3g04250	gene30673	0.13			Putative lysine decarboxylase family protein
gene30672	Fvb3:2402870-2403688	Fvb3	2402870	2403688	FvH4_3g04260	gene30672	1			Protein of unknown function (DUF1191)
gene30482	Fvb3:2414813-2419554	Fvb3	2414813	2419554	FvH4_3g04270	gene30482	0.03	GO:0005634,(	BUM,BUM1,SHL,STM,	KNOX/ELK homeobox transcription factor
gene36118	Fvb3:2434240-2445242	Fvb3	2434240	2445242	FvH4_3g04271	gene36118	1	GO:0046983		BED zinc finger ;hAT family dimerisation domain
gene39873	Fvb3:2437887-2441102	Fvb3	2437887	2441102	FvH4_3g04272	gene39873	1			
gene39874	Fvb3:2441838-2442306	Fvb3	2441838	2442306	FvH4_3g04273	gene39874	1			
gene34398	Fvb3:2445503-2447544	Fvb3	2445503	2447544	FvH4_3g04274	gene34398	1			
gene39875	Fvb3:2447567-2449053	Fvb3	2447567	2449053	FvH4_3g04275	gene39875	1			
gene34397	Fvb3:2451076-2451596	Fvb3	2451076	2451596	FvH4_3g04276	gene34397	1			
gene39876	Fvb3:2451879-2453196	Fvb3	2451879	2453196	FvH4_3g04277	gene39876	1			
gene30480	Fvb3:2454125-2454500	Fvb3	2454125	2454500	FvH4_3g04278	gene30480	1			
gene39877	Fvb3:2455276-2456618	Fvb3	2455276	2456618	FvH4_3g04279	gene39877	1			
gene30479	Fvb3:2456669-2457157	Fvb3	2456669	2457157	FvH4_3g04280	gene30479	1			
gene39878	Fvb3:2457754-2458089	Fvb3	2457754	2458089	FvH4_3g04281	gene39878	1		PRA1.B4	prenylated RAB acceptor 1.B4
gene30478	Fvb3:2463114-2463608	Fvb3	2463114	2463608	FvH4_3g04290	gene30478	0.35	GO:0046983		basic helix-loop-helix (bHLH) DNA-binding family protein
gene30477	Fvb3:2467937-2470515	Fvb3	2467937	2470515	FvH4_3g04300	gene30477	0.27			Putative lysine decarboxylase family protein
gene30475	Fvb3:2478741-2480461	Fvb3	2478741	2480461	FvH4_3g04320	gene30475	0.23	GO:0016491,GO:0055114		oxidoreductase, zinc-binding dehydrogenase family protein
gene30474	Fvb3:2481728-2482159	Fvb3	2481728	2482159	FvH4_3g04330	gene30474	0.69			RING/U-box superfamily protein
gene30473	Fvb3:2483914-2485758	Fvb3	2483914	2485758	FvH4_3g04340	gene30473	0.32	GO:0016491,GO:0055114		oxidoreductase, zinc-binding dehydrogenase family protein
gene30472	Fvb3:2486427-2490004	Fvb3	2486427	2490004	FvH4_3g04350	gene30472	0.29	GO:0003676,(	ENDO4	endonuclease 4
gene30471	Fvb3:2490330-2493176	Fvb3	2490330	2493176	FvH4_3g04360	gene30471	0.43	GO:0003676,(	BFN1,ENDO1	bifunctional nuclease i
gene30470	Fvb3:2493649-2503227	Fvb3	2493649	2503227	FvH4_3g04370	gene30470	0.21	GO:0016491,(	ATNDI1,NDA1	alternative NAD(P)H dehydrogenase 1
gene30469	Fvb3:2498348-2498629	Fvb3	2498348	2498629	FvH4_3g04371	gene30469	0.28			
gene30468	Fvb3:2501261-2501731	Fvb3	2501261	2501731	FvH4_3g04372	gene30468	1			
gene30467	Fvb3:2502379-2503545	Fvb3	2502379	2503545	FvH4_3g04380	gene30467	0.84			
gene30466	Fvb3:2506864-2508628	Fvb3	2506864	2508628	FvH4_3g04381	gene30466	1			
gene30465	Fvb3:2510283-2511340	Fvb3	2510283	2511340	FvH4_3g04390	gene30465	1			alpha/beta-Hydrolases superfamily protein
gene30464	Fvb3:2516385-2518781	Fvb3	2516385	2518781	FvH4_3g04400	gene30464	0	GO:0003677	PGA6,WUS,WUS1	Homeodomain-like superfamily protein
gene30463	Fvb3:2519250-2521670	Fvb3	2519250	2521670	FvH4_3g04401	gene30463	1			
gene30462	Fvb3:2526433-2539920	Fvb3	2526433	2539920	FvH4_3g04410	gene30462	0.25	GO:0003824,(	DME	HhH-GPD base excision DNA repair family protein
gene30460	Fvb3:2542466-2545728	Fvb3	2542466	2545728	FvH4_3g04430	gene30460	0.04	GO:0006790,(	AHL,ATAHL,HL	HAL2-like
gene30459	Fvb3:2548136-2549101	Fvb3	2548136	2549101	FvH4_3g04450	gene30459	1			
gene30458	Fvb3:2549192-2553125	Fvb3	2549192	2553125	FvH4_3g04440	gene30458	0.12	GO:0015031,(	SC3	secretory carrier 3
gene30457	Fvb3:2553666-2558651	Fvb3	2553666	2558651	FvH4_3g04460	gene30457	0.06			Protein of unknown function (DUF707)
gene30456	Fvb3:2561199-2564257	Fvb3	2561199	2564257	FvH4_3g04470	gene30456	0.01	GO:0005515	BRD4	bromodomain 4
gene36119	Fvb3:2565092-2573380	Fvb3	2565092	2573380	FvH4_3g04490	gene36119	0.06	GO:0008017,GO:0008352,GO:00510		Transducin/WD40 repeat-like superfamily protein
gene36120	Fvb3:2575125-2581786	Fvb3	2575125	2581786	FvH4_3g04500	gene36120	0.06	GO:0004672,(	SRF3	STRUBBELIG-receptor family 3
gene30454	Fvb3:2584220-2585013	Fvb3	2584220	2585013	FvH4_3g04510	gene30454	0.27			
gene30453	Fvb3:2587117-2588921	Fvb3	2587117	2588921	FvH4_3g04520	gene30453	1			
gene30452	Fvb3:2591487-2593892	Fvb3	2591487	2593892	FvH4_3g04521	gene30452	1	GO:0003677,GO:0046983		BED zinc finger ;hAT family dimerisation domain
gene30451	Fvb3:2595061-2596221	Fvb3	2595061	2596221	FvH4_3g04530	gene30451	0.01			
gene30450	Fvb3:2597499-2599924	Fvb3	2597499	2599924	FvH4_3g04540	gene30450	0.29			
gene30449	Fvb3:2603071-2603504	Fvb3	2603071	2603504	FvH4_3g04541	gene30449	1			
gene30448	Fvb3:2605809-2608029	Fvb3	2605809	2608029	FvH4_3g04550	gene30448	0.19			lysine decarboxylase family protein
gene30447	Fvb3:2612473-2613372	Fvb3	2612473	2613372	FvH4_3g04560	gene30447	1			
gene30446	Fvb3:2613547-2616827	Fvb3	2613547	2616827	FvH4_3g04570	gene30446	0.09	GO:0005515		LisH and RanBPM domains containing protein
gene30445	Fvb3:2618718-2631807	Fvb3	2618718	2631807	FvH4_3g04580	gene30445	0.04	GO:0005524	EDA16	SNF2 domain-containing protein / helicase domain-containing protein / zinc finger protein-related
gene30444	Fvb3:2627189-2628148	Fvb3	2627189	2628148	FvH4_3g04590	gene30444	0.02			alpha/beta-Hydrolases superfamily protein
gene30443	Fvb3:2633585-2637467	Fvb3	2633585	2637467	FvH4_3g04600	gene30443	0.18	GO:0004185,(	scpl31	serine carboxypeptidase-like 31
gene30442	Fvb3:2638003-2640481	Fvb3	2638003	2640481	FvH4_3g04610	gene30442	0.05	GO:0009690,(	ATCKX5,CKX7	cytokinin oxidase 7
gene30441	Fvb3:2645673-2649644	Fvb3	2645673	2649644	FvH4_3g04611	gene30441	1			
gene30440	Fvb3:2651305-2654139	Fvb3	2651305	2654139	FvH4_3g04620	gene30440	0.18	GO:0004185,(	scpl31	serine carboxypeptidase-like 31
gene30439	Fvb3:2655643-2657423	Fvb3	2655643	2657423	FvH4_3g04630	gene30439	0.13	GO:0003677,(	anac025,NAC025	NAC domain containing protein 25
gene30438	Fvb3:2662560-2663368	Fvb3	2662560	2663368	FvH4_3g04640	gene30438	1	GO:0005515,GO:0007165		Toll-Interleukin-Resistance (TIR) domain family protein
gene30437	Fvb3:2667733-2668380	Fvb3	2667733	2668380	FvH4_3g04641	gene30437	1			
gene36121	Fvb3:2668901-2673182	Fvb3	2668901	2673182	FvH4_3g04650	gene36121	0.09			disease resistance protein (TIR class), putative
gene36122	Fvb3:2673192-2677479	Fvb3	2673192	2677479	FvH4_3g04651	gene36122	0.09	GO:0003676,GO:0006364,GO:00084		Exonuclease family protein

Supplementary Table S8. Phenotypic data of the two sets of individual with extremes phenotypes for pelargonidin-3-glucoside and studied using microarray. The choice of genotypes was based on the level of PgGs in 2010 and according to the genotyping with the marker located in the peak of QTL: AX-89826440-M3a.

Genotype	year	Level	Ant	PgGs	PgGsM	PgRs	CyGs	AfPgGs	Fvo	KGs	KGn	KCoGs	QGn	F3ol	Cat	CatCat	AfCat	AfGs	UnK	UnK1	UnK2	ANTHc	FLAVc	PHENc	FRAP	TEAC	COLOUR
6	2010	high	40.49	37.67	0.528	1.134	1.15	0.013	0.616	0.169	0.12	0.106	0.221	1.706	0.327	0.757	0.083	0.539	1.161	0.896	0.265	965.8	630.5	1.771	8.665	14.07	
7	2010	high	28.84	26.79	0.16	1.324	0.546	0.016	0.442	0.183	0.082	0.11	0.067	1.983	0.223	0.478	0.044	1.238	0.874	0.685	0.189	486	577.6	1.477	8.285	14.73	
34	2010	high	38.89	36.67	0	1.325	0.881	0.016	0.414	0.153	0.052	0.172	0.037	1.19	0.206	0.458	0.052	0.474	1.3	0.961	0.339	1103	585	1.576	8.152	14.46	
36	2010	high	28.61	25.8	0.608	1.359	0.829	0.011	0.301	0.137	0.078	0.055	0.031	1.067	0.239	0.582	0.051	0.194	0.837	0.64	0.197	757.2	527.1	1.1	6.786	12.51	
85	2010	high	33.67	31.4	0.174	1.275	0.809	0.011	0.313	0.095	0.074	0.099	0.045	1.098	0.243	0.578	0.068	0.208	1.025	0.767	0.258	761.1	659.7	1.64	8.964	16.19	
198	2010	high	36.28	33.06	0.24	2.314	0.642	0.021	0.325	0.109	0.064	0.071	0.082	2.637	0.233	0.56	0.066	1.778	0.982	0.725	0.257	1014	582.8	1.638	9.792	16.61	
216	2010	high	32.15	28.59	0.275	2.894	0.37	0.02	0.325	0.091	0.063	0.132	0.04	0.866	0.207	0.409	0.045	0.205	0.905	0.691	0.215	1048	405.8	1.627	7.756	14.21	
16	2010	low	27.74	26.61	0.133	0.814	0.163	0.019	0.308	0.108	0.085	0.077	0.038	1.211	0.235	0.565	0.061	0.351	0.849	0.663	0.186	661.1	261.6	1.124	7.423	12.45	
19	2010	low	20.97	19.69	0.091	0.957	0.232	0.006	0.17	0.037	0.055	0.045	0.033	1.093	0.216	0.458	0.041	0.378	0.6	0.477	0.123	661	294.3	1.354	7.623	13.78	
46	2010	low	14.97	14.08	0.015	0.734	0.13	0.01	0.168	0.065	0.029	0.051	0.023	1.178	0.246	0.476	0.048	0.408	0.465	0.356	0.109	352.5	347.7	0.892	6.751	12.4	
78	2010	low	18.25	16.8	0.023	1.167	0.253	0.009	0.253	0.073	0.047	0.075	0.057	1.469	0.22	0.435	0.069	0.745	0.472	0.374	0.098	515.7	482.8	1.249	6.832	13.23	
94	2010	low	19.07	18.11	0	0.828	0.132	0.007	0.177	0.037	0.062	0.038	0.041	0.665	0.171	0.315	0.029	0.15	0.561	0.409	0.152	476.1	386.8	1.047	6.221	12.31	
111	2010	low	18.01	16.66	0	1.176	0.165	0.003	0.301	0.096	0.076	0.087	0.043	1.155	0.245	0.507	0.045	0.358	0.5	0.396	0.104	672.8	456.9	1.175	6.907	13.18	
180	2010	low	15.85	14.63	0	1.091	0.125	0.004	0.182	0.062	0.042	0.058	0.021	0.972	0.199	0.38	0.039	0.354	0.541	0.421	0.12	484.1	296.5	1.05	4.652	9.84	
6	2011	high	38.87	33.59	0.356	3.969	0.488	0.47	0.354	0.155	0.099	0.065	0.035	1.713	0.316	0.708	0.092	0.596	1.214	0.932	0.282	1085	447.2	4.428		35.48	5.75
7	2011	high	28.6	24.74	0.373	2.41	0.714	0.365	0.4	0.188	0.09	0.082	0.04	1.525	0.244	0.525	0.048	0.709	0.994	0.745	0.249	744.9	358.3	2.908		29.09	4
34	2011	high	32.14	27.03	0	4.376	0.452	0.287	0.227	0.121	0.034	0.05	0.023	1.235	0.188	0.442	0.041	0.565	0.981	0.751	0.23	1200	399.5	2.398		33.75	5.5
36	2011	high	34.04	28.49	0.963	3.687	0.52	0.385	0.282	0.155	0.071	0.036	0.02	0.915	0.195	0.461	0.052	0.207	1.034	0.81	0.224	1220	469.2	2.792		39.98	6
85	2011	high	34.19	28.51	0	4.732	0.61	0.338	0.243	0.085	0.076	0.052	0.03	1.159	0.24	0.584	0.085	0.249	1.01	0.794	0.216	1052	384	2.259		33.36	3.25
198	2011	high	34.17	29.74	0	3.443	0.497	0.49	0.189	0.104	0.036	0.024	0.026	2.567	0.268	0.579	0.09	1.631	0.927	0.732	0.195	1028	424.7	2.426		33.53	5.25
216	2011	high	36.15	28.02	1.242	6.243	0.353	0.289	0.268	0.127	0.056	0.058	0.028	1.209	0.304	0.713	0.077	0.116	0.998	0.758	0.239	1102	591.6	2.84		39.53	6
16	2011	low	24.99	20.75	0.071	3.656	0.271	0.246	0.373	0.145	0.096	0.078	0.053	1.467	0.354	0.787	0.069	0.257	0.771	0.617	0.154	726.6	417.7	2.487		36.62	2
19	2011	low	21.82	19.37	0	2.029	0.174	0.248	0.167	0.064	0.062	0.019	0.022	1.084	0.189	0.371	0.051	0.472	0.75	0.595	0.155	765.3	390.9	2.457		34.48	1.75
46	2011	low	19.57	17.46	0.101	1.547	0.306	0.152	0.271	0.093	0.058	0.064	0.056	1.576	0.284	0.704	0.057	0.531	0.483	0.37	0.114	438.5	426	2.414		33.44	0.75
78	2011	low	23.45	19.63	0.074	3.185	0.324	0.237	0.259	0.116	0.047	0.05	0.045	1.538	0.284	0.619	0.098	0.536	0.603	0.489	0.115	758.3	433.6	2.366		36.74	2.25
94	2011	low	22.09	19.6	0.078	2.093	0.185	0.128	0.273	0.102	0.084	0.051	0.036	0.953	0.283	0.523	0.06	0.088	0.723	0.545	0.178	607.1	366.7	2.262		31.06	1
111	2011	low	27.46	22.98	0	3.414	0.858	0.207	0.297	0.148	0.054	0.06	0.035	0.837	0.18	0.401	0.045	0.211	0.8	0.625	0.174	979.7	358.2	2.203		31.83	5.25
180	2011	low	24.03	19.48	0	4.115	0.228	0.204	0.237	0.103	0.072	0.039	0.023	1.126	0.229	0.481	0.067	0.349	0.663	0.525	0.137	497.2	300.7	2.047		25.28	1.75

year	Ant.me	PgGs.m	PgGsM	PgRs.m	CyGs.m	AfPgGs	Fvo.me	KGs.me	KGn.m	KCoGs.	QGn.m	F3ol.m	Cat.me	CatCat.	AfCat.n	AfGs.m	UnK.m	UnK1.n	UnK2.n	ANTHc.	FLAVc.r	PHENc.	FRAP.m	TEAC.mean	
2010	4E-05	1E-04	0.022	0.034	8E-04	0.015	0.007	0.003	0.101	0.023	0.186	0.159	0.289	0.092	0.164	0.3	2E-04	2E-04	4E-04	0.006	6E-04	0.002	0.006	0.009	
2011	2E-05	3E-05	0.097	0.052	0.1	7E-04	0.739	0.209	0.898	0.941	0.119	0.317	0.82	0.807	0.624	0.292	5E-05	1E-04	5E-05	0.001	0.142	0.099		0.314	0.001

Supplementary Table S9. List of genes included in the support interval of M3A/3A colour-related QTLs that were tested by microarray. The support interval was on Fvb3 between 826.085 b to 2.673762 b. In this region, a total of 304 genes were tested on microarray. More particular attention were provided to three genes, underlined in yellow in this table. These three genes were considered as candidate genes for controlling the M3A/F3A colour-related QTLs.

Genotype	198	216	6	34	36	85	7	180	19	46	78	94	111	16							
Choice PgGs+genotype	high	high	high	high	high	high	high	low	low	low	low	low	low	low	red=<0.05	FvH4_name_v4.0.a2	LG_v4.0.a2	start_v4.0.a2	end_v4.0.a2	annot_v4.0.a2	Fvb
SEQ_ID																					
GENE19488	253.54	387.29	333.06	310.39	199.1	437.92	449.79	319.11	487.79	289.49	415.67	268.39	354.72	401.02	0.617	FvH4_3g01610	Fvb3	844553	846799	Tetratricopeptide repeat (TPR)-like superfamily protein	gene19488
GENE19486	1415.8	1009.3	1510.8	1470.8	1304.1	1397	1245.6	1266	1238	1105.5	1157.6	1392.1	1139.2	1405.7	0.265	FvH4_3g01630	Fvb3	852844	855245	Predicted pyridoxal phosphate-dependent enzyme, YBL036C type	gene19486
GENE19485	17.1	19.056	19.249	18.2	18.854	17.968	16.25	17.384	17.899	19.215	17.856	16.713	18.792	19.351	0.894	FvH4_3g01640	Fvb3	856439	857203	Stigma-specific Stig1 family protein	gene19485
GENE19484	14.858	15.629	16.285	14.538	14.822	16.234	15.548	17.058	14.449	15.605	14.392	16.235	14.78	17.359	0.611	FvH4_3g01660	Fvb3	858564	860252	Stigma-specific Stig1 family protein	gene19484
GENE19483	9.8175	9.7677	12.897	12.441	12.433	9.872	13.41	15.762	12.404	11.741	12.326	12.646	13.165	13.681	0.070	FvH4_3g01670	Fvb3	861992	863872	Stigma-specific Stig1 family protein	gene19483
GENE19482	9.9183	10.867	11.763	12.754	12.313	9.7237	11.108	9.8734	10.692	11.841	11.676	13.027	16.05	12.666	0.254	FvH4_3g01671	Fvb3	864360	864793	0	gene19482
GENE19481	20.952	21.617	26.815	24.164	24.963	23.153	29.161	23.454	28.788	26.119	28.942	29.118	31.127	29.373	0.025	FvH4_3g01672	Fvb3	865701	866343	Stigma-specific Stig1 family protein	gene19481
GENE19480	25.232	20.319	18.667	18.66	20.002	19.216	20.838	21.272	17.85	15.8	15.928	18.677	19.481	17.469	0.061	FvH4_3g01680	Fvb3	866815	869961	Pectin lyase-like superfamily protein	gene19480
GENE19479	56.329	54.696	78.009	108.66	142.73	119.81	46.716	110.58	42.351	67.479	148.43	142.72	32.892	36.724	0.879	FvH4_3g01690	Fvb3	868323	869998	0	gene19479
GENE19478	3693.2	6451	4425.6	5473.7	4776.6	6078.1	6690.3	2653.4	5646	5019.7	3451.5	4222	5659.9	8064.2	0.615	FvH4_3g01700	Fvb3	877530	880706	WRKY family transcription factor	gene19478
GENE19477	13582	18076	14915	18612	10706	16854	14245	18304	16548	22339	20170	17539	21036	14473	0.042	FvH4_3g01710	Fvb3	881776	885746	aspartic proteinase A1	gene19477
GENE19476	252.61	301.97	302.93	353.51	320.27	403	225.55	287.62	312.39	340.36	323.55	307.34	141.91	157.13	0.303	FvH4_3g01720	Fvb3	887358	888988	mitogen-activated protein kinase kinase kinase 16	gene19476
GENE19475	19.667	26.172	24.277	36.229	24.722	30.18	19.379	44.954	30.628	23.164	28.467	23.559	22.708	29.516	0.404	FvH4_3g01730	Fvb3	889181	892415	topoisomerase II	gene19475
GENE19474	10.062	10.818	11.307	11.378	9.9167	12.217	10.139	12.125	9.774	10.921	10.779	10.038	9.3758	10.469	0.487	FvH4_3g01740	Fvb3	896142	897214	subtilisin-like serine protease 3	gene19474
GENE19473	19.706	19.442	24.243	26.679	24.861	20.936	27.627	16.275	29.302	29.058	27.276	26.882	30.952	23.839	0.230	FvH4_3g01741	Fvb3	899967	901443	0	gene19473
GENE19472	12.87	13.883	13.142	12.775	12.283	14.46	12.694	13.876	12.922	13.595	12.223	14.121	13.907	13.904	0.385	FvH4_3g01742	Fvb3	901962	903170	B-cell receptor-associated 31-like	gene19472
GENE19471	23.874	22.392	20.331	33.703	20.693	25.055	21.171	24.729	27.128	26.457	24.583	20.686	24.99	24.925	0.653	FvH4_3g01743	Fvb3	903802	904234	RNA-binding KH domain-containing protein	gene19471
GENE19470	8768	9159.7	6909.7	9761.7	7232	7428.7	7585.2	6114.7	6949.8	6302	5986.1	7163.1	4636.1	7921.9	0.012	FvH4_3g01750	Fvb3	904187	906850	B-cell receptor-associated 31-like	gene19470
GENE19469	7455.7	8353.4	8387.7	9608.4	4852.9	6404.9	5352.7	2297.3	5386.2	5204.6	6442.3	3426.7	5474.6	5661.9	0.018	FvH4_3g01760	Fvb3	907610	909061	S-adenosyl-L-methionine-dependent methyltransferases superfamily protein	gene19469
GENE19647	138.73	106.88	73.695	131.94	71.489	80.801	62.966	48.357	77.202	82.162	80.213	59.901	63.071	85.095	0.090	FvH4_3g01780	Fvb3	920697	923085	S-adenosyl-L-methionine-dependent methyltransferases superfamily protein	gene19647
GENE19646	46.047	21.337	41.223	11.099	30.551	54.033	37.333	11.352	13.99	10.676	14.707	17.118	15.218	15.512	0.010	FvH4_3g01790	Fvb3	923544	924854	0	gene19646
GENE19645	12845	10043	10938	13284	10624	10277	8290.6	9806.7	10148	10210	9920.3	9516.9	8020.5	9241.1	0.091	FvH4_3g01800	Fvb3	927006	930248	Peroxisomal membrane 22 kDa (Mpv17/PMP22) family protein	gene19645
GENE19644	76.68	27.704	31.594	26.149	40.73	107.98	47.055	37.157	41.261	57.208	32.046	40.961	37.386	48.391	0.472	FvH4_3g01810	Fvb3	931371	932945	F-box family protein with a domain of unknown function (DUF295)	gene19644
GENE19643	1115	976.96	1745.7	1143.5	1056.2	1161.5	705.65	1430	1009.7	1067.6	1533.5	2119.2	1015.1	571.5	0.599	FvH4_3g01820	Fvb3	933825	937208	Glycine cleavage T-protein family	gene19643
GENE19641	52.279	22.656	29.102	28.501	29.548	35.02	31.389	30.507	25.976	15.674	31.519	21.156	29.774	16.876	0.088	FvH4_3g01830	Fvb3	937913	939770	Homeodomain-like superfamily protein	gene19641
GENE19640	142.14	207.6	275.82	289.61	40.162	331.94	99.694	337.06	99.73	50.204	798.01	122.4	302.73	41.004	0.647	FvH4_3g01840	Fvb3	945420	949637	SKU5 similar 4	gene19640
GENE19639	16.868	17.13	19.214	16.651	19.1	20.025	16.47	17.464	16.002	18.374	17.923	19.653	18.85	18.059	0.863	FvH4_3g01850	Fvb3	950160	953560	Rhamnogalacturonate lyase family protein	gene19639
GENE19638	198.79	183.12	173.36	311.76	177.06	236.21	145.9	264.34	250.04	164.3	175.7	143.94	131.49	142.52	0.464	FvH4_3g01860	Fvb3	954306	958361	endonuclease/exonuclease/phosphatase family protein	gene19638
GENE19636	4802.9	3935.6	3676	2528	3650.4	3717.2	3223.2	5834.2	4630.4	3279.2	4106.4	4128.9	4488.9	3336.4	0.172	FvH4_3g01900	Fvb3	967939	970787	APS reductase 3	gene19636
GENE19635	504.93	225.33	527.25	338.07	570.74	394.64	304.94	144.68	260.91	431.19	183.74	341.6	302.44	323.55	0.064	FvH4_3g01910	Fvb3	975062	976231	60S acidic ribosomal protein family	gene19635
GENE19634	24625	23260	25715	24786	25359	23238	28059	26948	27629	27779	26268	27434	25722	24745	0.053	FvH4_3g01920	Fvb3	978864	981677	glyoxalase I homolog	gene19634
GENE19633	25.801	17.26	18.891	19.005	30.812	30.607	24.722	23.682	26.705	23.814	21.89	20.345	23.872	22.275	0.783	FvH4_3g01921	Fvb3	981811	983217	SIN3-like 1	gene19633
GENE19632	10.506	8.9138	10.786	9.9733	11.553	9.0345	8.9865	9.1733	8.798	8.3711	9.9072	10.912	12.531	8.6466	0.775	FvH4_3g01930	Fvb3	984124	985287	SIN3-like 3	gene19632
GENE19631	153.2	147.22	158.85	150.32	126.24	136.6	126.54	270.17	113.43	114.55	96.656	132.67	72.56	204.56	0.977	FvH4_3g01940	Fvb3	985544	990088	arogenate dehydratase 1	gene19631
GENE19630	43.767	45.965	46.006	40.469	47.068	47.368	36.975	42.216	38.311	36.182	43.175	49.92	48.718	42.101	0.684	FvH4_3g01950	Fvb3	992934	995725	Ankyrin repeat family protein	gene19630
GENE19629	7156.1	5087.4	6934	6780.4	5707.6	4851.6	4861	2667.7	4780.6	4694.1	4354.7	6031.4	3327.9	6374.4	0.064	FvH4_3g01980	Fvb3	997175	999038	Ubiquitin-like superfamily protein	gene19629
GENE19628	26.745	46.03	42.985	37.224	32.71	44.928	37.381	87.816	36.541	38.726	59.413	47.874	74.863	36.536	0.084	FvH4_3g02000	Fvb3	1E+06	1E+06	0	gene19628
GENE19627	62.003	32.853	67.602	89.197	160.7	55.469	68.131	26.308	89.354	27.697	44.507	42.893	43.042	47.738	0.111	FvH4_3g02010	Fvb3	1E+06	1E+06	0	gene19627
GENE19626	18.187	20.682	26.358	29.51	29.25	32.484	23.552	24.278	21.786	22.799	19.987	47.515	14.701	22.365	0.836	FvH4_3g02020	Fvb3	1E+06	1E+06	Galactosyltransferase family protein	gene19626
GENE19625	171.78	317.31	263.35	233.43	261.87	572.14	336.26	456.88	353.2	687.07	455.95	675.13	264.25	397.01	0.059	FvH4_3g02040	Fvb3	1E+06	1E+06	starch synthase 3	gene19625
GENE19624	15.237	18.022	12.223	13.786	13.747	14.579	13.103	17.315	12.055	15.299	17.892	16.516	16.731	13.946	0.244	FvH4_3g02050	Fvb3	1E+06	1E+06	0	gene19624
GENE19623	33.847	164.29	243.9	93.262	71.501	169.36	61.631	196.84	89.729	291.38	513.86	670.91	204.49	77.034	0.091	FvH4_3g02060	Fvb3	1E+06	1E+06	ATPase, F1 complex, gamma subunit protein	gene19623
GENE19622	340.24	604.92	416.66	412.93	798.75	728.69	782.87	1175.6	755.45	719.12	448.33	540.54	920.26	1047.6	0.106	FvH4_3g02070	Fvb3	1E+06	1E+06	Homeodomain-like superfamily protein	gene19622
GENE19620	16.599	18.794	22.212	18.65	22.044	19.227	21.653	20.036	23.326	18.57	21.561	17.294	18.456	28.462	0.481	FvH4_3g02071	Fvb3	1E+06	1E+06	C-terminal domain phosphatase-like 4	gene19620
GENE19618	19.752	18.195	12.292	18.929	16.428	17.781	16.833	16.291	15.646	16.989	15.604	16.552	16.485	14.976	0.290	FvH4_3g02100	Fvb3	1E+06	1E+06	0	gene19618
GENE19617	17.487	18.321	20.127	23.875	21.402	19.778	19.69	19.26	19.621	22.697	21.09	17.108	20.94	25.553	0.548	FvH4_3g02101	Fvb3	1E+06	1E+06	F-box family protein	gene19617
GENE19615	2018.6	4214.5	2979.8	4588	3997.1	3536.5	3694.3	4449.9	3514.9	6532.8	2200.3	4582.8	2520.2	4686.7	0.462	FvH4_3g02130	Fvb3	1E+06	1E+06	calcium-dependent protein kinase 21	gene19615
GENE19614	31.303	23.455	30.93	23.339	21.879	40.08	54.044	26.617	35.746	22.602	56.998	20.192	22.033	22.438	0.699	FvH4_3g02131	Fvb3	1E+06	1E+06	0	gene19614
GENE19613	5718.9	8347.4	5935.3	4879.9	4975	5530	5440.9	7123.9	4788.7	6920.4	7972.9	6945	5671.9	9855.3	0.139	FvH4_3g02140	Fvb3	1E+06	1E+06	Protein of unknown function, DUF584	gene19613
GENE19612	41.206	29.656	22.381	25.027	28.35	27.844	30.856	25.674	22.982	25.925	25.213	23.434	28.239	29.142	0.183	FvH4_3g02150	Fvb3	1E+06	1E+06	0	gene19612
GENE19611	15.324	13.222	10.939	12.966	15.784	12.869	20.537	16.147	10.675	15.761	11.796	11.485	13.5	10.585	0.280	FvH4_3g02160	Fvb3	1E+06	1E+06	0	gene19611
GENE19608	27.889	39.189	539.87	655.11	173.95	406.5	431.08	50.707	306.24	270.2	75.626	263.56	46.651	130.76	0.153	FvH4_3g02170	Fvb3	1E+06	1E+06	oxidoreductase, zinc-binding dehydrogenase family protein	gene19608
GENE19607	13.15	13.287	16.361	14.799	14.591	12.857	12.565	13.934	13.64	11.444	13.941	14.672	15.755	12.947	0.805	FvH4_3g02171	Fvb3	1E+06	1E+06	0	gene19607

GENE19606	17.718	17.251	12.105	16.157	14.659	13.29	13.278	18.524	15.684	12.968	15.224	16.384	16.35	21.318	0.210	FvH4_3g02190	Fvb3	1E+06	1E+06	oxidoreductase, zinc-binding dehydrogenase family protein	gene19606
GENE19604	16.117	13.132	16.799	13.65	14.101	14.74	12.619	13.214	16.045	18.224	14.661	12.935	12.28	14.01	0.976	FvH4_3g02200	Fvb3	1E+06	1E+06	UDP-glucose 6-dehydrogenase family protein	gene19604
GENE19603	20.082	19.188	19.515	18.809	21.874	21.103	14.181	20.899	16.588	24.217	18.187	19.347	17.214	17.595	0.943	FvH4_3g02201	Fvb3	1E+06	1E+06	0	gene19603
GENE19602	737.12	578.45	377.04	626.69	715.42	570.04	774.79	607.16	706.73	654.74	413.05	611.77	395.95	588.01	0.415	FvH4_3g02210	Fvb3	1E+06	1E+06	0	gene19602
GENE19621	36.553	55.661	74.299	85.662	45.221	56.042	52.785	30.154	64.819	85.505	70.76	58.928	94.736	62.926	0.400	FvH4_3g02220	Fvb3	1E+06	1E+06	0	gene19621
GENE19600	23.567	51.13	30.017	30.039	23.82	29.104	61.467	33.831	36.122	30.143	36.743	119	63.69	24.543	0.351	FvH4_3g02260	Fvb3	1E+06	1E+06	MATE efflux family protein	gene19600
GENE19599	25.485	22.866	22.474	26.186	22.13	22.976	22.042	21.64	23.965	23.176	26.952	27.045	22.522	25.786	0.360	FvH4_3g02270	Fvb3	1E+06	1E+06	0	gene19599
GENE19598	4329.1	5603.8	4082.4	3384.4	5231.3	4754.7	5004.9	6263.3	4934.5	4708.3	5272.7	5020.6	6306.2	3807.9	0.225	FvH4_3g02280	Fvb3	1E+06	1E+06	switch subunit 3	gene19598
GENE19597	20.439	30.151	22.596	23.985	24.533	16.682	24.938	17.1	20.54	21.381	15.346	15.986	17.202	19.204	0.017	FvH4_3g02290	Fvb3	1E+06	1E+06	NB-ARC domain-containing disease resistance protein	gene19597
GENE19596	5440.7	6509.8	5863	5828.4	6602.5	7031.9	7493.9	7760	6780.1	7174.8	7131.3	7079.1	7012.3	6337.5	0.073	FvH4_3g02300	Fvb3	1E+06	1E+06	ATP binding cassette protein 1	gene19596
GENE19595	1813.7	2532.5	2208.4	2616.1	1831.8	2732.8	1902.1	2291.2	1842.6	2576.6	2287.3	2508.6	1813.6	1900.1	0.762	FvH4_3g02310	Fvb3	1E+06	1E+06	DNA binding	gene19595
GENE19594	12482	10424	11682	11554	12397	11857	11522	10173	11014	11212	10971	12811	10819	10569	0.154	FvH4_3g02320	Fvb3	1E+06	1E+06	RNA-binding (RRM/RBD/RNP motifs) family protein	gene19594
GENE19593	1721.3	2705	1862.7	2626.4	1945.6	2675.1	1736.8	2545.8	1582.4	1384.2	2387.1	2197.5	1965.8	1085.9	0.283	FvH4_3g02321	Fvb3	1E+06	1E+06	0	gene19593
GENE19592	180.32	137.44	145.7	78.694	201.83	129.82	91.055	126.03	217.95	329.49	93.895	204.71	120.49	370.55	0.142	FvH4_3g02330	Fvb3	1E+06	1E+06	HSP20-like chaperones superfamily protein	gene19592
GENE19591	18.348	25.962	16.465	21.522	24.17	22.66	28.098	39.599	22.675	18.7	22.654	22.988	17.251	13.474	0.996	FvH4_3g02340	Fvb3	1E+06	1E+06	binding;calmodulin binding	gene19591
GENE19590	12307	10993	12030	12628	11360	11774	10033	8911.2	10779	10223	10481	10854	9854.9	11327	0.017	FvH4_3g02350	Fvb3	1E+06	1E+06	DERLIN-2.2	gene19590
GENE19588	217.14	305.79	347.11	315.26	368.25	292.57	410.69	474.35	477.81	357.21	409.6	364.02	506.57	402.1	0.007	FvH4_3g02370	Fvb3	1E+06	1E+06	pentatricopeptide repeat 336	gene19588
GENE19587	13.907	12.056	14.408	14.344	15.432	14.047	14.389	15.17	12.042	13.767	12.863	15.331	14.782	11.884	0.572	FvH4_3g02390	Fvb3	1E+06	1E+06	UDP-glucose 6-dehydrogenase family protein	gene19587
GENE19586	138.75	90.475	72.027	28.947	98.424	46.146	108.14	26.28	43.593	32.718	34.5	46.904	25.793	28.094	0.013	FvH4_3g02400	Fvb3	1E+06	1E+06	SWIB complex BAF60b domain-containing protein	gene19586
GENE19584	64.2	41.895	80.412	62.856	81.551	77.35	65.995	60.782	77.513	78.327	84.061	61.227	97.988	81.055	0.210	FvH4_3g02421	Fvb3	1E+06	1E+06	0	gene19584
GENE19583	1599.9	1805.6	1478.6	1157.8	1632.6	1354.8	1946.9	1538.4	2060.4	1140.6	1632.3	1779.5	1932.3	1017.4	0.922	FvH4_3g02430	Fvb3	1E+06	1E+06	cardiolipin synthase	gene19583
GENE19582	15.278	12.058	15.627	15.148	13.942	16.106	13.81	14.787	13.023	15.129	13.936	14.942	15.171	13.807	0.789	FvH4_3g02440	Fvb3	1E+06	1E+06	Protein kinase superfamily protein	gene19582
GENE19581	18.622	22.706	22.572	24.397	27.346	28.897	22.686	24.917	22.347	24.012	30.913	50.501	17.532	18.494	0.513	FvH4_3g02450	Fvb3	1E+06	1E+06	0	gene19581
GENE19580	1361.2	1201.7	816.19	698.04	1169.4	1650.6	1277.3	1095.2	1532	1340.2	1205.2	1145	1030.3	1161.9	0.736	FvH4_3g02460	Fvb3	1E+06	1E+06	phospholipases;galactolipases	gene19580
GENE19579	16.763	17.142	18.012	16.954	18.721	18.464	18.713	21.327	19.421	16.694	19.368	16.959	22.926	19.598	0.105	FvH4_3g02470	Fvb3	1E+06	1E+06	LA RNA-binding protein	gene19579
GENE19578	315.69	273.96	422.65	482.61	424.13	328.69	335.71	311.06	304.31	337.95	414.18	495.18	310.21	260.64	0.614	FvH4_3g02490	Fvb3	1E+06	1E+06	adenosine/AMP deaminase family protein	gene19578
GENE19577	131.73	133.81	159.17	105.91	135.03	98.568	166.85	62.669	92.955	64.308	91.199	60.456	56.457	58.228	0.000	FvH4_3g02480	Fvb3	1E+06	1E+06	Pseudouridine synthase family protein	gene19577
GENE19573	2006.5	2940.7	2329.4	1516.4	3094	3103.6	3336.4	3332.9	3231.8	3156.1	2676.4	2735	4466.3	3238.7	0.083	FvH4_3g02510	Fvb3	1E+06	1E+06	PCF11P-similar protein 4	gene19573
GENE19572	591.81	874.47	907.81	988.19	629.1	1149.9	556.73	966.53	894.53	933.38	854.84	569.72	1001.1	713.95	0.750	FvH4_3g02520	Fvb3	1E+06	1E+06	protodermal factor 2	gene19572
GENE19570	18.483	20.377	17.42	18.809	17.421	17.031	19.416	17.538	16.459	19.3	24.135	18.218	22.499	19.76	0.291	FvH4_3g02530	Fvb3	1E+06	1E+06	COBRA-like protein 11 precursor	gene19570
GENE19568	136.59	275.73	273.74	218.1	353.08	288.93	122.92	544.58	86.282	139.02	181.1	281.48	65.394	171.16	0.691	FvH4_3g02550	Fvb3	1E+06	1E+06	NTF2-like	gene19568
GENE19567	133.75	93.8	63.397	31.495	110.51	164.17	185.01	102.74	41.145	63.786	54.869	82.221	37.876	86.093	0.080	FvH4_3g02560	Fvb3	1E+06	1E+06	methylesterase PCR A	gene19567
GENE19566	1734.4	910.93	617.54	612.37	633.04	1051.3	806.6	531.44	464.67	769.55	408.78	461.26	515.43	782.32	0.065	FvH4_3g02570	Fvb3	1E+06	1E+06	PAK-box/P21-Rho-binding family protein	gene19566
GENE19564	21.458	24.746	23.225	30.51	27.178	27.12	21.554	25.862	27.097	24.476	28.052	26.442	23.541	23.466	0.761	FvH4_3g02571	Fvb3	1E+06	1E+06	0	gene19564
GENE19563	151.34	180.47	236.12	229	160.71	179.31	272.62	325.84	311.45	238.61	387.43	306.28	462.04	250.23	0.005	FvH4_3g02572	Fvb3	1E+06	1E+06	ferrochelatase 2	gene19563
GENE19562	15.571	11.53	11.002	12.592	13.394	11.486	11.519	11.122	13.997	13.078	12.328	13.1	13.101	13.625	0.523	FvH4_3g02580	Fvb3	1E+06	1E+06	F-box/RNI-like superfamily protein	gene19562
GENE19561	113.75	171.96	202.01	128.69	196.88	128.78	207.04	135.41	225.41	184.07	267.26	164.81	266.23	217.21	0.091	FvH4_3g02581	Fvb3	1E+06	1E+06	1-amino-cyclopropane-1-carboxylate synthase 12	gene19561
GENE19560	41.369	36.519	37.427	51.537	50.377	42.928	40.518	45.716	45.26	41.992	42.836	49.549	44.09	42.358	0.533	FvH4_3g02590	Fvb3	1E+06	1E+06	0	gene19560
GENE19559	10.147	8.8853	9.8747	8.3324	9.8471	8.9605	8.7332	9.7866	10.294	8.9248	9.4069	10.133	9.5442	8.1312	0.602	FvH4_3g02591	Fvb3	1E+06	1E+06	F-box/RNI-like superfamily protein	gene19559
GENE19558	25.685	21.907	22.88	29.136	28.575	27.248	24.337	36.398	30.147	26.7	28.292	28.814	27.44	25.613	0.073	FvH4_3g02593	Fvb3	1E+06	1E+06	0	gene19558
GENE19557	117.32	113.53	132.93	128.38	121.6	111.46	138.6	108.62	120.26	132.23	122.76	126.72	96.153	124.4	0.456	FvH4_3g02594	Fvb3	1E+06	1E+06	F-box family protein	gene19557
GENE19556	13986	17828	16497	15985	19081	17704	18810	20344	21193	18907	18465	21693	20648	18070	0.008	FvH4_3g02600	Fvb3	1E+06	1E+06	Oligosaccharyltransferase complex/magnesium transporter family protein	gene19556
GENE19555	63.748	26.196	27.764	28.414	44.657	49.05	48.684	36.071	46.558	45.039	29.475	27.094	19.795	34.25	0.292	FvH4_3g02620	Fvb3	1E+06	1E+06	Tetratricopeptide repeat (TPR)-like superfamily protein	gene19555
GENE19553	182.13	149.74	237.93	166.96	880.48	445.56	128.82	398.49	69.465	149.12	177.79	207.37	102.49	170.32	0.271	FvH4_3g02630	Fvb3	1E+06	1E+06	xyloglucan endotransglucosylase/hydrolase 8	gene19553
GENE19552	749.72	286.35	319.18	383.08	307.24	300.18	234.38	220.99	268.99	222.38	191.96	207.58	168.25	336.07	0.085	FvH4_3g02640	Fvb3	1E+06	1E+06	postsynaptic protein-related	gene19552
GENE19551	1141.3	969.62	1017.4	961.37	904.48	1124	946.79	1108.5	1070.4	867.48	1017.4	878.74	1017.1	849.99	0.502	FvH4_3g02641	Fvb3	1E+06	1E+06	polyubiquitin 10	gene19551
GENE19550	259.21	285.17	318.56	356.02	258.71	422.23	382.87	342.22	520.69	421.58	356.34	361.73	508.06	344.5	0.053	FvH4_3g02650	Fvb3	1E+06	1E+06	tyrosyl-DNA phosphodiesterase-related	gene19550
GENE19547	32.507	31.676	28.854	27.674	26.997	28.023	28.636	30.124	28.832	29.731	29.564	29.01	29.035	27.751	0.958	FvH4_3g02671	Fvb3	1E+06	1E+06	0	gene19547
GENE19546	192.16	312.13	245.24	254.54	269.91	302.64	277.44	368.68	319.66	338.25	261.34	387.53	307.32	258.55	0.041	FvH4_3g02680	Fvb3	1E+06	1E+06	PPDK regulatory protein	gene19546
GENE19545	12420	8400.6	5173.7	4205.3	6428.7	5146.9	4494.3	7154.3	4472.9	5445.9	5467.1	4945.5	5327.6	4304.3	0.297	FvH4_3g02690	Fvb3	1E+06	1E+06	0	gene19545
GENE19544	54.791	35.11	45.666	52.235	34.129	62.847	58.316	74.299	45.432	43.19	54.152	55.308	43.347	32.889	0.906	FvH4_3g02700	Fvb3	1E+06	1E+06	Peroxidase superfamily protein	gene19544
GENE19543	27.946	33.782	31.625	35.954	29.177	39.878	38.605	90.267	33.483	29.79	33.808	42.322	25.148	29.388	0.465	FvH4_3g02720	Fvb3	1E+06	1E+06	Late embryogenesis abundant (LEA) hydroxyproline-rich glycoprotein family	gene19543
GENE19542	348.67	217.52	342.38	295.14	239.54	226.9	260.69	180.46	276.86	187.68	159.64	184.69	189.26	132.23	0.006	FvH4_3g02730	Fvb3	1E+06	1E+06	Trimeric LpxA-like enzymes superfamily protein	gene19542
GENE19541	119.6	324.58	102.93	153.4	86.67	103.65	89.665	79.962	70.93	85.829	46.946	72.471	60.187	89.616	0.079	FvH4_3g02731	Fvb3	1E+06	1E+06	cysteine-rich RLK (RECEPTOR-like protein kinase) 25	gene19541
GENE19539	22.173	28.33	23.484	23.977	23.662	29.088	24.021	25.063	29.817	22.078	24.839	23.177	28.501	21.268	0.999	FvH4_3g02752	Fvb3	1E+06	1E+06	cysteine-rich RLK (RECEPTOR-like protein kinase) 25	gene19539
GENE19538	8.9929	11.467	9.2994	8.9115	10.505	11.451	9.4762	11.796	8.7057	9.7399	9.968	9.0265	13.729	9.7347	0.649	FvH4_3g02753	Fvb3	1E+06	1E+06	0	gene19538
GENE19537	30.184	56.206	130.04	110.62	25.452	123.38	37.652	53.506	139.05	134.54	78.846	70.713	144.91	79.403	0.260	FvH4_3g02754	Fvb3	1E+06	1E+06	cysteine-rich RLK (RECEPTOR-like protein kinase) 25	gene19537
GENE19534	19.701	24.086	21.91	28.18	22.159	27.829	26.333	24.047	25.817	16.35	18.616	14.852	15.283	18.363	0.025	FvH4_3g02770	Fvb3	1E+06	1E+06	cysteine-rich RLK (RECEPTOR-like protein kinase) 25	gene19534
GENE19533	28.146	32.587	36.602	33.049	28.412	25.612	33.524	28.083	31.049	31.055	30.225	27.643	27.887	35.716	0.629	FvH4_3g02771	Fvb3	1E+06	1E+06	cysteine-rich RLK (RECEPTOR-like protein kinase) 10	gene19533
GENE19532	156.02	367.22	324.82	53.489	578.88	52.69	254.29	53.741	51.087	48.812	36.105	29.057	58.357	45.654	0.026	FvH4_3g02772	Fvb3	1E+06	1E+06	cysteine-rich RLK (RECEPTOR-like protein kinase) 10	gene19532
GENE19530	26.657	54.833	45.444	76.087	39.274	52.959	52.015	82.737	50.129	92.7	75.921	80.541	50.982	63.668	0.027	FvH4_3g02773	Fvb3	1E+06	1E+06	cysteine-rich RLK (RECEPTOR-like protein kinase) 25	gene19530
GENE19528	24.26	42.216	42.624	39.235	37.801	31.121	50.853	36.845	27.853	40.775	49.633	56.531	33.356	50.017	0.463	FvH4_3g02774	Fvb3	1E+06	1E+06	cysteine-rich RLK (RECEPTOR-like protein kinase) 25	gene19528
GENE19527	19.018	21.736	26.1	20.931	20.746	24.219	23.936	23.402	18.691	20.276	23.84	26.401	30.649	25.079	0.366	FvH4_3g02790	Fvb3	1E+06	1E+06	MATE efflux family protein	gene19527

GENE19526	19.437	19.739	26.227	21.774	23.616	18.627	24.187	20.395	16.999	21.314	23.893	24.924	20.559	23.357	0.837	FvH4_3g02801	Fvb3	1E+06	1E+06	cysteine-rich RLK (RECEPTOR-like protein kinase) 33	gene19526
GENE19525	211.74	393.89	288.78	564.3	291.79	450.54	381.67	641.58	332.95	722.37	550.48	604.99	368.68	422.98	0.056	FvH4_3g02820	Fvb3	1E+06	1E+06	cysteine-rich RLK (RECEPTOR-like protein kinase) 25	gene19525
GENE19524	14.515	17.821	13.383	12.226	17.305	14.461	15.346	13.356	12.073	12.814	12.763	15.506	14.023	13.868	0.112	FvH4_3g02830	Fvb3	1E+06	1E+06	cysteine-rich RLK (RECEPTOR-like protein kinase) 25	gene19524
GENE19523	42.185	52.983	71.268	55.913	61.223	65.114	55.827	65.503	50.3	55.851	62.199	61.093	68.353	53.452	0.693	FvH4_3g02831	Fvb3	1E+06	1E+06	MATE efflux family protein	gene19523
GENE19521	18.622	20.768	24.769	22.133	23.085	18.773	21.493	18.388	22.684	22.572	25.46	21.315	27.598	24.536	0.217	FvH4_3g02851	Fvb3	2E+06	2E+06	0	gene19521
GENE24648	121.36	73.775	89.394	82.09	141.21	92.198	150.26	95.53	93.853	160.76	89.16	80.412	126.69	87.513	0.885	FvH4_3g02852	Fvb3	2E+06	2E+06	0	gene24648
GENE24649	1477.8	561.82	793.55	1846.4	418.32	2128.1	1190.5	218.5	569.36	722.27	472.23	295.57	327.89	910.2	0.030	FvH4_3g02870	Fvb3	2E+06	2E+06	cysteine-rich RLK (RECEPTOR-like protein kinase) 29	gene24649
GENE24650	15.519	11.85	15.683	12.803	14.363	14.211	12.56	16.447	13.321	16.376	12.649	13.567	13.157	12.607	0.851	FvH4_3g02880	Fvb3	2E+06	2E+06	AGAMOUS-like 29	gene24650
GENE24651	13.701	19.731	11.554	10.979	19.145	17	14.915	19.427	25.659	14.094	16.582	14.653	18.668	11.247	0.406	FvH4_3g02881	Fvb3	2E+06	2E+06	0	gene24651
GENE24652	54.084	43.734	29.494	40.924	41.367	63.004	35.21	63.507	43.499	39.474	41.061	51.418	39.198	36.867	0.856	FvH4_3g02890	Fvb3	2E+06	2E+06	RNI-like superfamily protein	gene24652
GENE24653	288.68	769.81	536.41	1474.6	232.37	492.64	137.74	518.43	1079	1777.7	761.31	980.1	579.53	709.61	0.162	FvH4_3g02900	Fvb3	2E+06	2E+06	Sulfite exporter TauE/SafE family protein	gene24653
GENE24654	7702.5	4552.6	5495.8	4895.3	4629.8	5213.8	5009.2	4015.2	4570.9	4622.6	5154.8	4999	4714.9	3927.6	0.115	FvH4_3g02910	Fvb3	2E+06	2E+06	basic helix-loop-helix (bHLH) DNA-binding superfamily protein	gene24654
GENE24655	1581	1112.5	2220.5	1306	232	1045.8	1382.4	1402.9	469.48	619.53	1613.4	467.89	2396.2	356.54	0.561	FvH4_3g02911	Fvb3	2E+06	2E+06	C-terminal cysteine residue is changed to a serine 1	gene24655
GENE24656	328.57	468.51	222.5	476.82	452.73	403.92	576.79	314.44	293.58	182.38	205.12	206.1	171.42	187.15	0.003	FvH4_3g02912	Fvb3	2E+06	2E+06	kinesin 1	gene24656
GENE24657	44.787	154.97	205	81.463	53.398	139.92	71.277	327.14	69.519	317.57	145.08	398.56	60.879	66.362	0.166	FvH4_3g02920	Fvb3	2E+06	2E+06	photosystem II subunit Q-2	gene24657
GENE24658	11205	11520	11539	10989	13227	12268	11753	14190	12635	12460	11540	13189	11817	10941	0.249	FvH4_3g02930	Fvb3	2E+06	2E+06	DNA-binding storekeeper protein-related transcriptional regulator	gene24658
GENE24659	649.7	510.66	993.16	962.68	531.92	584.09	484.6	322.33	343.32	265.38	437.47	487.49	307.51	599.5	0.014	FvH4_3g02940	Fvb3	2E+06	2E+06	histone H2A 2	gene24659
GENE24660	38.358	99.251	75.112	63.235	52.821	62.194	43.851	168.2	50.409	58.331	71.31	181.88	44.8	61.365	0.255	FvH4_3g02950	Fvb3	2E+06	2E+06	Protein of unknown function (DUF1218)	gene24660
GENE24661	17854	13520	14158	14384	12341	15307	13772	18842	15860	16077	15088	16307	15391	13971	0.120	FvH4_3g02960	Fvb3	2E+06	2E+06	Aldolase-type TIM barrel family protein	gene24661
GENE24663	1856.4	1616.8	1342.9	1587.7	1370.9	1699.5	1693.5	1797.9	1656	1584.3	1937.9	1477.6	1983.6	1821.7	0.141	FvH4_3g02970	Fvb3	2E+06	2E+06	0	gene24663
GENE24664	27.296	35.418	27.912	29.391	29.628	42.157	48.32	25.618	33.21	34.331	36.108	27.227	60.796	28.237	0.891	FvH4_3g02971	Fvb3	2E+06	2E+06	0	gene24664
GENE24665	965.68	1607.4	2167.7	1967.9	988.13	1693.1	1245.2	1903.5	4307.2	3951.5	2826.2	2779	2048.5	2740.8	0.005	FvH4_3g02980	Fvb3	2E+06	2E+06	NAD(P)-binding Rossmann-fold superfamily protein	gene24665
GENE24666	26.54	23.35	24.92	22.677	24.044	26.759	27.155	25.482	32.037	24.355	23.884	21.623	22.985	26.997	0.855	FvH4_3g02990	Fvb3	2E+06	2E+06	Protein kinase superfamily protein	gene24666
GENE24667	4841.9	940.7	2308	3363.7	1841.2	1699.8	1842.5	711.77	203.84	5635.4	2376.7	3650.3	245.22	6013.8	0.793	FvH4_3g03000	Fvb3	2E+06	2E+06	terpene synthase 03	gene24667
GENE24668	728.25	1092.7	1137	823.83	1019.9	912.34	1458	1405.2	946.2	1058.6	1246.3	1252.2	1526.5	1042.2	0.148	FvH4_3g03010	Fvb3	2E+06	2E+06	RING/U-box superfamily protein	gene24668
GENE24669	4950.7	6140.6	5085.7	4118.8	5511.4	6046.5	6783.2	7291.2	6763.1	6194.1	7110.5	5609.5	7958.3	6312	0.018	FvH4_3g03030	Fvb3	2E+06	2E+06	eukaryotic translation initiation factor-related	gene24669
GENE24670	25.073	20.275	21.295	23.055	18.674	24.819	21.116	22.972	26.518	24.564	25.463	22.201	19.349	18.8	0.590	FvH4_3g03040	Fvb3	2E+06	2E+06	Phototropic-responsive NPH3 family protein	gene24670
GENE24671	1405.2	2544.1	1798.8	3275.2	2395.9	3010.4	2414.9	2562.9	2863.7	3764.1	2092.6	2237	1977.8	2643.2	0.590	FvH4_3g03041	Fvb3	2E+06	2E+06	phosphoinositide binding	gene24671
GENE24672	57.824	35.368	40.093	41.255	35.333	36.145	29.809	29.05	29.174	44.441	29.93	40.45	37.915	44.248	0.506	FvH4_3g03042	Fvb3	2E+06	2E+06	terpene synthase 14	gene24672
GENE34030	1262.3	942.19	1009.3	764.58	887.9	681	664.61	198.82	294	466.12	37.231	410.52	35.155	332.62	0.000	FvH4_3g03043	Fvb3	2E+06	2E+06	Tetratricopeptide repeat (TPR)-like superfamily protein	gene34030
GENE24675	2288.7	2462.4	3346.5	3620.8	3260.1	3246.3	3674.9	2744.3	5109.2	3297.4	4846.4	3897.3	5007.8	3332.8	0.056	FvH4_3g03060	Fvb3	2E+06	2E+06	Tetratricopeptide repeat (TPR)-like superfamily protein	gene24675
GENE24676	15186	11008	11877	11663	13589	9893.2	14878	10418	14278	10947	10333	10015	11615	16928	0.688	FvH4_3g03061	Fvb3	2E+06	2E+06	terpene synthase 14	gene24676
GENE24677	3348.3	3358.2	3448.7	2990.5	2803.4	2291.6	3455	2761.7	3784.3	2465.8	4376.7	3076.3	4829	2828.8	0.383	FvH4_3g03070	Fvb3	2E+06	2E+06	Tetratricopeptide repeat (TPR)-like superfamily protein	gene24677
GENE24678	33.114	28.355	82.231	98.613	96.962	94.027	105.42	21.217	100.57	101.54	76.997	66.223	84.272	80.809	0.951	FvH4_3g03071	Fvb3	2E+06	2E+06	phosphoinositide binding	gene24678
GENE24673	13.447	13.445	15.762	14.043	16.258	11.826	13.672	12.235	15.064	15.297	16.434	13.81	17.467	11.977	0.581	FvH4_3g03080	Fvb3	2E+06	2E+06	Tetratricopeptide repeat (TPR)-like superfamily protein	gene24673
GENE30670	1245.7	636.45	137.9	95.662	989.25	1371.6	64.34	369.19	1319.9	21.411	861.86	1307.1	55.289	1309.3	0.751	FvH4_3g03130	Fvb3	2E+06	2E+06	S-adenosyl-L-methionine-dependent methyltransferases superfamily protein	gene30670
GENE30669	11790	7402.9	9802.5	8625	10268	6492.2	11105	7596.9	11069	7977.3	7815.4	7082.7	9590.4	15363	0.916	FvH4_3g03150	Fvb3	2E+06	2E+06	terpene synthase 14	gene30669
GENE30668	2254.5	2484.7	2810.5	3299.5	2848.7	3340.8	3349.5	2388.2	4736	2535.6	3853	3106.4	3611.9	2194	0.470	FvH4_3g03160	Fvb3	2E+06	2E+06	Tetratricopeptide repeat (TPR)-like superfamily protein	gene30668
GENE30667	33.317	22.314	27.542	42.019	34.72	29.205	25.415	27.046	26.753	31.121	29.292	33.081	35.479	28.206	0.859	FvH4_3g03170	Fvb3	2E+06	2E+06	basic helix-loop-helix (bHLH) DNA-binding superfamily protein	gene30667
GENE30665	57.089	56.814	66.857	58.558	62.535	73.085	81.568	46.932	79.12	62.356	94.075	88.97	87.206	60.853	0.263	FvH4_3g03200	Fvb3	2E+06	2E+06	basic helix-loop-helix (bHLH) DNA-binding superfamily protein	gene30665
GENE30664	14990	10433	12254	11738	10268	10767	9336	6512.1	10022	9659.4	10725	9182.9	7067	12942	0.097	FvH4_3g03210	Fvb3	2E+06	2E+06	flavodoxin-like quinone reductase 1	gene30664
GENE30663	1618.8	1597	1618.9	1624	785.53	1243.7	1779.3	985.63	1183.8	844.53	1382	1014.3	1320.5	1749.5	0.166	FvH4_3g03220	Fvb3	2E+06	2E+06	plant U-box 8	gene30663
GENE30662	49.084	66.466	74.665	90.706	90.835	126.16	69.915	118	76.468	58.051	75.997	56.962	47.078	69.835	0.477	FvH4_3g03230	Fvb3	2E+06	2E+06	S-locus lectin protein kinase family protein	gene30662
GENE30661	6608.5	5731.9	5417	6763.3	6104.5	5397.6	7160.3	5137.7	4355.7	3922.5	3546.4	2925.8	3412.8	3473.2	0.000	FvH4_3g03231	Fvb3	2E+06	2E+06	S-locus lectin protein kinase family protein	gene30661
GENE30660	36.241	33.386	61.558	89.119	71.802	64.703	60.542	42.615	69.037	79.031	53.849	43.186	38.857	52.731	0.570	FvH4_3g03240	Fvb3	2E+06	2E+06	S-locus lectin protein kinase family protein	gene30660
GENE30659	29.853	22.144	29.385	32.006	31.096	25.623	29.377	18.733	21.471	17.981	22.875	21.697	23.669	25.881	0.002	FvH4_3g03241	Fvb3	2E+06	2E+06	S-locus lectin protein kinase family protein	gene30659
GENE30658	583.58	664.36	428.05	986.99	554.77	605.79	305.46	369.43	404.69	464.01	388.26	218.34	108.96	261.73	0.015	FvH4_3g03242	Fvb3	2E+06	2E+06	S-locus lectin protein kinase family protein	gene30658
GENE30657	31.083	31.79	29.651	35.287	33.636	28.812	33.314	28.361	36.768	36.857	35.225	29.654	40.085	41.894	0.119	FvH4_3g03243	Fvb3	2E+06	2E+06	S-locus lectin protein kinase family protein	gene30657
GENE30656	43.84	37.117	36.578	46.761	37.72	27.576	56.944	27.9	39.222	29.071	41.434	31.501	48.199	37.395	0.332	FvH4_3g03250	Fvb3	2E+06	2E+06	crooked neck protein, putative / cell cycle protein, putative	gene30656
GENE30655	24.539	24.793	24.252	25.616	26.25	28.156	26.161	21.728	27.292	23.452	27.817	26.796	27.965	25.105	0.958	FvH4_3g03251	Fvb3	2E+06	2E+06	0	gene30655
GENE30654	650.74	589.82	401.81	304.42	669.06	278.36	1086.2	491.48	472.79	728.54	406.43	703.38	426.51	1565.3	0.548	FvH4_3g03260	Fvb3	2E+06	2E+06	cytokinin oxidase/dehydrogenase 6	gene30654
GENE30653	40.395	44.016	52.79	62.638	50.837	49.076	56.02	59.192	73.409	53.056	68.482	50.073	60.423	60.947	0.034	FvH4_3g03280	Fvb3	2E+06	2E+06	RNAse I inhibitor protein 2	gene30653
GENE30652	17.05	17.634	19.387	21.987	20.448	19.507	21.444	17.417	25.689	26.329	23.482	19.994	28.025	27.226	0.028	FvH4_3g03300	Fvb3	2E+06	2E+06	S-locus lectin protein kinase family protein	gene30652
GENE30651	20.147	23.191	18.566	23.42	23.187	19.385	19.403	21.578	26.295	22.929	17.273	19.165	21.259	24.121	0.597	FvH4_3g03301	Fvb3	2E+06	2E+06	S-locus lectin protein kinase family protein	gene30651
GENE30650	114.37	204.95	163.42	261.88	125.81	126.25	181.47	116.02	88.345	135.82	148.67	127.47	111.76	119.06	0.059	FvH4_3g03310	Fvb3	2E+06	2E+06	S-locus lectin protein kinase family protein	gene30650
GENE30649	155.78	244.04	143.11	228.14	215.76	127.74	160.14	238.1	85.08	244.4	130.12	162.22	94.156	247.75	0.753	FvH4_3g03320	Fvb3	2E+06	2E+06	S-locus lectin protein kinase family protein	gene30649
GENE30646	85.783	69.281	113.6	112.45	98.841	66.856	146.33	77.642	107.63	78.649	114.64	116.84	119.47	143.09	0.515	FvH4_3g03324	Fvb3	2E+06	2E+06	0	gene30646
GENE30645	22.965	14.743	16.779	13.696	19.208	14.712	22.796	13.605	11.224	9.5045	10.905	12.505	13.314	11.84	0.005	FvH4_3g03325	Fvb3	2E+06	2E+06	0	gene30645
GENE34404	120.4	129.26	142.8	203.32	117.24	99.203	145.66	124.44	91.153	144.57	106.88	93.983	82.149	168.05	0.250	FvH4_3g03340	Fvb3	2E+06	2E+06	S-locus lectin protein kinase family protein	gene34404
GENE30644	1028.2	701.55	898.99	1133.3	597.1	676.5	809.09	559.28	219.63	582.47	527.63	468.87	289.48	1001.2	0.024	FvH4_3g03350	Fvb3	2E+06	2E+06	S-locus lectin protein kinase family protein	gene30644
GENE30643	206.57	470.98	396.8	548.27	394.58	540.27	511.88	275.19	257.53	365.6	356.71	286.93	352.75	472.77	0.089	FvH4_3g03370	Fvb3	2E+06	2E+06	S-locus lectin protein kinase family protein	gene30643
GENE30642	35.7	52.055	49.278	70.99	27.025	33.784	31.605	36.159	51.408	50.472	46.277	44.573	40.619	45.013	0.753	FvH4_3g03390	Fvb3	2E+06	2E+06	S-locus lectin protein kinase family protein	gene30642
GENE30641	931.52	372.88	581.5	521.79	360.53	456.46	384.16	381.28	445.8	423.95	419.46	477.47	382.67	424.46	0.269	FvH4_3g03400	Fvb3	2E+06	2E+06	lysine decarboxylase family protein	gene30641
GENE30640	149.47	261.71	230.23	518.77	116.11	286.75	229.71	86.496	141.87	142.57	95.418	87.712	59.504	324.41	0.067	FvH4_3g03410	Fvb3	2E+06	2E+06	S-locus lectin protein kinase family protein	gene30640

GENE30639	46.754	32.637	38.858	37.498	31.302	27.099	47.79	49.272	66.81	56.517	25.701	34.007	33.182	30.978	0.467	FvH4_3g03420	Fvb3	2E+06	2E+06	receptor kinase 3	gene30639
GENE30638	114.25	170.1	177.83	253.44	287.84	280.26	188.23	222.7	258.47	261.1	197.31	172.51	168.66	210.51	0.924	FvH4_3g03430	Fvb3	2E+06	2E+06	receptor kinase 3	gene30638
GENE30637	415.38	509.3	423.77	787.01	352.77	545.88	385.43	447.55	475.98	411.13	482.16	207.09	517.33	533.9	0.495	FvH4_3g03431	Fvb3	2E+06	2E+06	S-locus lectin protein kinase family protein	gene30637
GENE30636	17.667	14.919	17.053	15.653	14.786	15.964	13.634	15.416	17.318	14.058	13.663	14.568	13.411	13.746	0.172	FvH4_3g03432	Fvb3	2E+06	2E+06	S-locus lectin protein kinase family protein	gene30636
GENE30635	86.471	86.54	95.372	92.38	70.927	90.72	119.82	84.397	86.087	69.243	109.4	71.629	73	67.354	0.167	FvH4_3g03433	Fvb3	2E+06	2E+06	S-domain-1 29	gene30635
GENE30633	55.456	42.702	48.781	66.027	53.347	61.512	49.363	42.888	51.989	50.364	47.271	46.384	47.431	38.534	0.057	FvH4_3g03451	Fvb3	2E+06	2E+06	S-locus lectin protein kinase family protein	gene30633
GENE30630	28.933	26.587	25.258	21.474	23.525	25.669	22.885	26.796	24.515	20.505	28.4	23.667	27.372	20.903	0.841	FvH4_3g03480	Fvb3	2E+06	2E+06	1-amino-cyclopropane-1-carboxylate synthase 8	gene30630
GENE30631	49.418	55.2	116.46	110.83	116.54	84.334	129.66	30.856	31.023	35.223	44.222	38.182	33.083	38.77	0.003	FvH4_3g03482	Fvb3	2E+06	2E+06	S-locus lectin protein kinase family protein	gene30631
GENE30752	443.2	507.07	495	773.5	580.69	369.98	736.56	514.33	234.64	405.33	529.65	536.53	135.76	1471.7	0.951	FvH4_3g03520	Fvb3	2E+06	2E+06	S-domain-1 29	gene30752
GENE30751	155.51	281.56	339.26	441.51	380.42	181.59	612.51	349.5	80.932	265	279.25	353.19	84.983	941.65	0.966	FvH4_3g03521	Fvb3	2E+06	2E+06	S-domain-1 29	gene30751
GENE30750	41.786	140.2	124.41	148.82	41.412	43.938	40.267	139.32	113.01	130.99	269.68	350.03	142.98	96.225	0.045	FvH4_3g03530	Fvb3	2E+06	2E+06	floral meristem identity control protein LEAFY (LFY)	gene30750
GENE30749	36.982	65.986	30.786	41.338	42.695	42.662	67.592	34.292	59.298	27.636	50.389	51.516	37.645	50.505	0.734	FvH4_3g03540	Fvb3	2E+06	2E+06	NAC (No Apical Meristem) domain transcriptional regulator superfamily protein	gene30749
GENE30748	23.927	25.246	29.68	29.403	31.733	24.686	26.088	26.255	26.316	25.479	27.727	25.964	33.342	28.791	0.777	FvH4_3g03560	Fvb3	2E+06	2E+06	S-domain-1 29	gene30748
GENE30746	44.058	53.634	47.459	41.918	51.55	50.678	55.111	36.275	56.902	39.669	49.209	60.725	46.67	45.723	0.735	FvH4_3g03580	Fvb3	2E+06	2E+06	NAC domain containing protein 100	gene30746
GENE30745	102.76	93.059	125.42	151.71	108.93	92.227	162.23	90.162	100.27	97.451	116.03	102.68	141.53	132.53	0.549	FvH4_3g03581	Fvb3	2E+06	2E+06	S-domain-1 29	gene30745
GENE30744	1038.4	2457	2477.7	2134.8	1935.1	3074.9	2020.9	2324.9	1540.1	1887	2408.5	2004.6	1944.2	2430.4	0.756	FvH4_3g03590	Fvb3	2E+06	2E+06	S-locus lectin protein kinase family protein	gene30744
GENE30743	65.63	219.4	109.99	168.3	72.55	75.346	70.1	51.904	57.905	61.492	61.687	59.827	76.403	59.917	0.068	FvH4_3g03600	Fvb3	2E+06	2E+06	Calcium-binding EF-hand family protein	gene30743
GENE30741	141.26	105.62	138.19	136.98	114.31	133.33	111.88	142.43	122.09	119.72	88.331	116.68	80.93	108.18	0.157	FvH4_3g03630	Fvb3	2E+06	2E+06	AGAMOUS-like 24	gene30741
GENE30740	73.214	25.169	18.591	31.714	19.477	26.272	24.527	16.392	16.604	19.233	15.315	17.754	16.453	20.341	0.103	FvH4_3g03640	Fvb3	2E+06	2E+06	B-box type zinc finger family protein	gene30740
GENE30739	148.06	219.33	301.26	201.04	275.83	254.57	435.21	406.64	340.63	335.84	242.28	451.84	336.94	431.53	0.041	FvH4_3g03650	Fvb3	2E+06	2E+06	Adenine nucleotide alpha hydrolases-like superfamily protein	gene30739
GENE30737	3158.6	2160	2536	1946.5	2362.4	2555.5	1871.3	1472	2452	2495	2591.8	2751.3	1712.9	1995.4	0.530	FvH4_3g03670	Fvb3	2E+06	2E+06	PLAC8 family protein	gene30737
GENE30736	613.83	201.37	188.49	187.94	207.82	215.65	280.03	56.469	116.45	117.91	99.054	158.73	57.732	280.06	0.055	FvH4_3g03680	Fvb3	2E+06	2E+06	myb domain protein 58	gene30736
GENE30735	35.78	37.88	47.844	53.304	42.346	51.579	44.238	60.826	43.592	48.308	65.293	83.446	36.898	38.553	0.221	FvH4_3g03690	Fvb3	2E+06	2E+06	Protein of unknown function (DUF594)	gene30735
GENE30734	28.794	28.809	36.317	34.887	33.955	27.26	26.89	22.409	31.218	21.299	40.319	37.05	40.963	27.402	0.880	FvH4_3g03700	Fvb3	2E+06	2E+06	Serine protease inhibitor (SERPIN) family protein	gene30734
GENE30732	18.284	17.627	17.975	15.27	19.666	18.643	17.308	15.077	17.188	20.578	16.736	19.875	19.786	19.73	0.537	FvH4_3g03710	Fvb3	2E+06	2E+06	Serine protease inhibitor (SERPIN) family protein	gene30732
GENE30731	17.388	20.196	23.869	21.165	17.017	17.2	16.629	20.397	17.386	23.596	28.621	28.037	18.705	21.149	0.105	FvH4_3g03720	Fvb3	2E+06	2E+06	Cyclin D6;1	gene30731
GENE30730	165.11	915.14	758.18	486.57	1001.6	665.64	772.42	1342.8	338.96	802.59	1066.4	1921.1	985.2	1260.1	0.078	FvH4_3g03730	Fvb3	2E+06	2E+06	Octicosapeptide/Phox/Bem1p family protein	gene30730
GENE30729	126.55	104.71	88.535	107.86	66.432	108.13	64.619	67.69	47.14	46.929	79.419	81.068	43.314	38.703	0.006	FvH4_3g03740	Fvb3	2E+06	2E+06	Major facilitator superfamily protein	gene30729
GENE30728	51.106	42.233	45.406	50.391	34.951	57.419	42.411	64.057	34.072	29.11	41.451	27.793	56.618	30.32	0.370	FvH4_3g03750	Fvb3	2E+06	2E+06	Major facilitator superfamily protein	gene30728
GENE30727	828.58	1162.7	1279.4	1932.5	1602.6	1913.5	1895.1	1522.9	1705.9	1835.9	923.35	1207.9	1020.7	1617.5	0.609	FvH4_3g03760	Fvb3	2E+06	2E+06	Zinc finger, RING-type;Transcription factor jumonji/aspartyl beta-hydroxylase	gene30727
GENE30726	748.34	1947	1964.7	2315.5	1673.7	1799.9	1829.3	1675.9	2172.9	1864	1827.4	1782	1755.7	1989.6	0.580	FvH4_3g03770	Fvb3	2E+06	2E+06	Protein kinase superfamily protein	gene30726
GENE30725	30.218	77.13	50.935	55.257	54.694	42.175	44.67	90.76	51.812	96.984	95.983	79.224	71.277	76.285	0.004	FvH4_3g03780	Fvb3	2E+06	2E+06	MYB-like 102	gene30725
GENE30724	190.77	354.15	569.36	368.54	581.24	594.72	647.57	331.56	936.36	616.36	358.56	691.05	367.41	520.21	0.498	FvH4_3g03790	Fvb3	2E+06	2E+06	Inositol monophosphatase family protein	gene30724
GENE30723	262.42	873.14	485.38	336.14	985.47	741.92	1153.9	799.53	1119.9	961.72	905.28	805.56	1417.4	414.15	0.216	FvH4_3g03810	Fvb3	2E+06	2E+06	Wound-responsive family protein	gene30723
GENE30722	27.927	26.282	32.42	34.458	31.527	31.19	32.586	26.023	37.83	34.177	37.162	36.971	38.384	29.933	0.128	FvH4_3g03821	Fvb3	2E+06	2E+06	0	gene30722
GENE30720	2011	2611	3486	2214.5	2614.2	2309.2	3970.7	2491.3	3212.9	2456.4	3418.6	3064.8	4021.6	2507.5	0.441	FvH4_3g03840	Fvb3	2E+06	2E+06	uridine 5'-monophosphate synthase / UMP synthase (PYRE-F) (UMPS)	gene30720
GENE30719	26.808	37.817	31.676	21.615	43.795	52.256	46.927	38.259	70.711	42.663	40.502	47.656	31.591	21.972	0.528	FvH4_3g03870	Fvb3	2E+06	2E+06	0	gene30719
GENE30718	2214	2538.9	3162.5	2883.3	3055.7	2913.3	3551.3	2297.7	3098.2	3127.3	2660.4	3031.1	2343.1	3691.6	0.969	FvH4_3g03880	Fvb3	2E+06	2E+06	PapD-like superfamily protein	gene30718
GENE30717	12.866	14.67	12.994	13.011	13.304	13.161	14.13	13.191	13.921	12.411	14.019	13.541	17.435	13.877	0.379	FvH4_3g03881	Fvb3	2E+06	2E+06	0	gene30717
GENE30716	22.246	19.235	18.788	17.621	18.444	20.782	18.961	18.326	20.644	18.089	18.571	19.67	24.647	14.407	0.855	FvH4_3g03882	Fvb3	2E+06	2E+06	0	gene30716
GENE30715	21629	20577	20401	19759	21987	21724	23707	22485	21676	24931	21723	22874	21653	19235	0.417	FvH4_3g03900	Fvb3	2E+06	2E+06	sugar transporter 1	gene30715
GENE30714	32.906	37.983	66.284	62.133	43.769	36.77	64.291	31.622	98.841	36.287	73.285	65.812	143.22	56.933	0.178	FvH4_3g03910	Fvb3	2E+06	2E+06	Tetratricopeptide repeat (TPR)-like superfamily protein	gene30714
GENE30713	55.242	35.968	45.419	44.322	42.573	24.374	42.317	27.836	38.612	27.292	40.753	40.577	35.622	50.706	0.400	FvH4_3g03920	Fvb3	2E+06	2E+06	0	gene30713
GENE30712	4257.3	4019.4	4692.5	4242.4	5298.5	4931.4	4997.2	5654.8	5173	6302.2	4898.7	5376.1	4302.2	6481.6	0.037	FvH4_3g03930	Fvb3	2E+06	2E+06	ARM repeat superfamily protein	gene30712
GENE30711	35.622	36.716	54.178	47.59	45.424	49.416	42.431	30.038	48.064	42.983	60.101	58.624	64.734	48.285	0.279	FvH4_3g03931	Fvb3	2E+06	2E+06	0	gene30711
GENE30710	36.985	35.134	29.469	33.882	41.204	28.456	31.098	31.342	30.933	28.653	30.875	37.968	31.93	43.143	0.940	FvH4_3g03932	Fvb3	2E+06	2E+06	zinc knuckle (CCHC-type) family protein	gene30710
GENE30709	72.668	51.704	59.076	88.61	50.267	119.04	44.528	43.954	52.171	81.633	62.114	38.211	62.325	66.647	0.351	FvH4_3g03940	Fvb3	2E+06	2E+06	F-box family protein	gene30709
GENE30708	17.026	17.651	24.009	17.23	23.082	19.776	19.843	22.045	22.579	20.257	25.559	21.461	21.222	21.881	0.088	FvH4_3g03950	Fvb3	2E+06	2E+06	Dynein light chain type 1 family protein	gene30708
GENE30707	11202	13050	11387	15797	14065	11145	10465	11462	13446	12969	11453	13451	10645	14049	0.954	FvH4_3g03960	Fvb3	2E+06	2E+06	NAD(P)H dehydrogenase B2	gene30707
GENE30706	14313	10951	13220	12789	12402	10966	12251	10220	9667.3	11163	10124	11815	10438	10542	0.006	FvH4_3g03970	Fvb3	2E+06	2E+06	0	gene30706
GENE30705	5968.1	3393.6	3917.9	3509.9	3957.8	6258.8	4433.8	4822.8	1626.8	2254.4	2233.2	1532.3	2064.4	4443.4	0.022	FvH4_3g03980	Fvb3	2E+06	2E+06	0	gene30705
GENE30704	13.362	16.9	21.759	16.972	15.535	15.925	15.354	14.721	16.178	16.339	15.675	16.348	16.014	16.825	0.618	FvH4_3g03990	Fvb3	2E+06	2E+06	F-box family protein	gene30704
GENE30703	2607.1	2085.8	2682.1	2192	1541.2	1751.5	1622.1	1676.3	1386	1597.1	1804.2	2096.4	1928.1	1831.1	0.146	FvH4_3g04000	Fvb3	2E+06	2E+06	SGNH hydrolase-type esterase superfamily protein	gene30703
GENE30702	127.85	153.11	284.78	341.87	215.71	106.86	302.44	127.57	318.45	171.14	308.57	336.59	183.23	257.49	0.614	FvH4_3g04010	Fvb3	2E+06	2E+06	Auxin-responsive GH3 family protein	gene30702
GENE30701	9.6065	9.0979	8.9914	8.411	7.808	10.489	8.6347	8.6725	11.543	8.3671	7.4732	7.267	8.4333	9.0297	0.618	FvH4_3g04011	Fvb3	2E+06	2E+06	0	gene30701
GENE30700	25.314	30.516	34.396	32.657	30.544	32.379	33.209	29.489	31.609	32.309	32.376	33.755	36.479	36.651	0.217	FvH4_3g04012	Fvb3	2E+06	2E+06	actin related protein 2	gene30700
GENE30699	80.967	52.349	115.2	484.29	96.226	192.6	75.764	113.89	78.635	80.587	74.986	63.74	61.784	58.066	0.209	FvH4_3g04020	Fvb3	2E+06	2E+06	F-box family protein	gene30699
GENE30698	366.41	648.56	486.76	483.3	505.8	609.35	429.78	645.19	409.57	535.76	353.94	495.88	235.22	365.85	0.292	FvH4_3g04030	Fvb3	2E+06	2E+06	Transducin/WD40 repeat-like superfamily protein	gene30698
GENE30697	38.507	43.293	38.73	43.059	39.81	31.711	42.902	27.766	40.087	38.57	43.802	47.044	54.983	39.063	0.604	FvH4_3g04031	Fvb3	2E+06	2E+06	0	gene30697
GENE30696	2317.8	3494.7	2794.1	3010.7	2901.3	2416.2	1868.1	3555.4	2679.6	3850.9	3121.1	3411.5	1784.2	3874.3	0.179	FvH4_3g04040	Fvb3	2E+06	2E+06	riboflavin kinase/FMN hydrolase	gene30696
GENE30695	823.06	642.49	833.93	737.77	977.21	1025.9	1115.3	1063.5	694.49	703.06	684.25	607.28	948.97	749.15	0.278	FvH4_3g04050	Fvb3	2E+06	2E+06	Transducin/WD40 repeat-like superfamily protein	gene30695
GENE30694	893.8	1434.3	1343.6	2582.3	943.25	1476	1752.5	895.81	661.95	1055.8	1036.7	723.88	1246.3	1168.8	0.055	FvH4_3g04060	Fvb3	2E+06	2E+06	Calmodulin-binding protein	gene30694
GENE30693	23.139	19.845	17.807	20.987	18.148	13.962	17.281	14.025	16.617	14.985	19.602	15.905	18.877	14.796	0.116	FvH4_3g04070	Fvb3	2E+06	2E+06	0	gene30693

GENE30692	9058.4	9240.7	9570.3	10235	7643.5	8395.4	8977.8	8059.9	8099.3	9331.4	8332.9	7179.8	8157.5	8718.7	0.087	FvH4_3g04090	Fvb3	2E+06	2E+06	sphingosine kinase 1	gene30692
GENE30691	46.136	53.027	66.641	56.706	39.432	33.154	47.03	35.207	27.475	28.001	32.395	41.898	35.879	33.98	0.010	FvH4_3g04100	Fvb3	2E+06	2E+06	Haloacid dehalogenase-like hydrolase (HAD) superfamily protein	gene30691
GENE30689	23.986	25.835	27.867	32.608	26.476	34.503	28.374	24.425	30.003	28.231	35.386	29.068	45.312	26.679	0.381	FvH4_3g04130	Fvb3	2E+06	2E+06	syntaxin of plants 124	gene30689
GENE30688	12353	12373	10760	12658	11276	10887	12192	10497	10852	10398	11022	10764	10999	10467	0.010	FvH4_3g04140	Fvb3	2E+06	2E+06	Vacuolar protein sorting-associated protein VPS28 family protein	gene30688
GENE30687	4804	6839	4923.6	4737.2	6807.5	7018.5	7162.5	7348.8	7414.6	6574.9	5768.6	6093.3	6892.2	6204.4	0.277	FvH4_3g04150	Fvb3	2E+06	2E+06	Tetratricopeptide repeat (TPR)-like superfamily protein	gene30687
GENE30685	900.93	1265.6	2598.6	3118.5	1279.2	2308.6	2489.3	978.92	1024.9	1376.6	1465.1	1727.1	1153.3	1511.2	0.081	FvH4_3g04160	Fvb3	2E+06	2E+06	Protein of unknown function (DUF761)	gene30685
GENE30684	25.223	28.612	31.821	25.298	25.352	27.151	26.706	20.981	32.401	29.729	31.247	28.778	32.017	34.931	0.170	FvH4_3g04161	Fvb3	2E+06	2E+06	0	gene30684
GENE30683	17.461	45.287	49.847	51.977	20.519	19.993	20.592	49.469	37.086	42.297	102.14	100.69	50.172	31.378	0.064	FvH4_3g04170	Fvb3	2E+06	2E+06	floral meristem identity control protein LEAFY (LFY)	gene30683
GENE30682	16.818	15.305	16.973	16.296	15.34	16.237	16.522	15.354	15.12	18.187	18.525	18.107	19.029	16.571	0.141	FvH4_3g04180	Fvb3	2E+06	2E+06	1-amino-cyclopropane-1-carboxylate synthase 8	gene30682
GENE30680	66.025	112.34	68.91	75.448	132.04	147.32	152.42	117.39	290.78	149.68	157.95	131.05	196.49	77.24	0.109	FvH4_3g04200	Fvb3	2E+06	2E+06	homolog of yeast sucrose nonfermenting 4	gene30680
GENE30679	6803.9	7249.3	5243.9	7490.6	6576.5	7128.6	6934.3	5464.9	6599.5	5922	5943.4	5943.4	4775.5	6360.5	0.026	FvH4_3g04220	Fvb3	2E+06	2E+06	ARF-GAP domain 5	gene30679
GENE30678	4817.6	4739.8	4227.3	4641.8	4787.5	3656	3841.7	3614.8	4570.8	3372.5	3519.3	4303.6	4011.1	3343.1	0.048	FvH4_3g04232	Fvb3	2E+06	2E+06	Protein of unknown function (DUF300)	gene30678
GENE30677	109.36	152.15	128.93	97.306	197.54	179.66	262.81	134.16	280.1	340.22	159.09	149.83	218.72	172.91	0.223	FvH4_3g04233	Fvb3	2E+06	2E+06	0	gene30677
GENE30676	86.057	90.436	204.2	165.62	256.78	167.63	139.27	98.306	249.18	224.91	92.995	242.25	58.916	361.47	0.526	FvH4_3g04240	Fvb3	2E+06	2E+06	0	gene30676
GENE30675	1969.5	1797.7	1557.1	1653	1755.3	1387.3	1326.6	1424.1	1661.4	1191	1116.4	1394.6	1315	1288.8	0.021	FvH4_3g04241	Fvb3	2E+06	2E+06	Protein of unknown function (DUF300)	gene30675
GENE30674	1897.8	1914.7	5365.1	5048.7	4478.7	3391.8	5255	1521	4320.2	3710.3	5748.6	5545.4	7128.1	6418.9	0.296	FvH4_3g04242	Fvb3	2E+06	2E+06	0	gene30674
GENE30673	2032.5	1323.3	2490.8	1892.8	1889.3	1935.8	2357.5	1043.2	1893.4	2003.4	1300.7	1833	1702.1	1928.6	0.135	FvH4_3g04250	Fvb3	2E+06	2E+06	Putative lysine decarboxylase family protein	gene30673
GENE30672	387.41	413.74	642.18	466.76	457.1	341.46	494.97	261.55	494.22	446.1	798.94	431.36	845.71	694.15	0.253	FvH4_3g04260	Fvb3	2E+06	2E+06	Protein of unknown function (DUF1191)	gene30672
GENE30482	455.58	611.96	966.55	1122.6	435.83	423.88	346.48	549.37	1037.4	878.69	1678.2	994.33	1670.6	698.26	0.050	FvH4_3g04270	Fvb3	2E+06	2E+06	KNOX/ELK homeobox transcription factor	gene30482
GENE34398	27.459	30.211	49.771	46.48	48.865	51.556	49.619	33.107	41.066	45.459	49.016	45.258	63.577	62.378	0.383	FvH4_3g04274	Fvb3	2E+06	2E+06	0	gene34398
GENE34397	4338	67.507	680.79	101.65	115.85	2439.4	2886.4	4436.1	89.094	74.044	57.233	60.715	41.194	1597.2	0.510	FvH4_3g04276	Fvb3	2E+06	2E+06	0	gene34397
GENE30479	7.9145	10.208	12.226	11.953	7.6026	9.5217	11.251	6.8219	10.541	9.9928	9.7666	10.946	11.602	8.648	0.722	FvH4_3g04280	Fvb3	2E+06	2E+06	0	gene30479
GENE30478	49.632	62.888	46.021	49.408	44.892	34.197	41.482	41.69	42.64	43.611	38.952	47.182	43.9	45.694	0.341	FvH4_3g04290	Fvb3	2E+06	2E+06	basic helix-loop-helix (bHLH) DNA-binding family protein	gene30478
GENE30477	10970	12746	11332	10840	12152	9360.7	7591.2	11170	8584.1	12227	8355.4	12547	6632.6	12850	0.748	FvH4_3g04300	Fvb3	2E+06	2E+06	Putative lysine decarboxylase family protein	gene30477
GENE30475	1192.6	1455.8	2024.3	1701.3	1349.8	1445	1680.9	1351.9	1388	1142.3	1029.7	1524.6	1415.5	1711.2	0.199	FvH4_3g04320	Fvb3	2E+06	2E+06	oxidoreductase, zinc-binding dehydrogenase family protein	gene30475
GENE30474	70.929	48.166	55.336	50.878	38.867	101.26	44.179	55.474	63.797	51.531	141.2	81.414	84.863	29.674	0.392	FvH4_3g04330	Fvb3	2E+06	2E+06	RING/U-box superfamily protein	gene30474
GENE30473	11178	12482	12305	12378	10781	9764.2	10229	13392	14055	14962	11776	14344	11666	9330.5	0.115	FvH4_3g04340	Fvb3	2E+06	2E+06	oxidoreductase, zinc-binding dehydrogenase family protein	gene30473
GENE30472	120.54	256.98	412.2	416.69	510.39	365.57	278.67	394.49	458.02	589.75	237.5	694.1	205.15	390.66	0.314	FvH4_3g04350	Fvb3	2E+06	2E+06	endonuclease 4	gene30472
GENE30471	113.56	129.6	126.38	111.17	116.19	194.8	83.568	182.74	167.95	181.69	111.06	112.27	136.89	127.56	0.261	FvH4_3g04360	Fvb3	2E+06	2E+06	bifunctional nuclease i	gene30471
GENE30470	28.207	207.86	47.829	263.7	31.896	83.361	26.873	50.543	71.799	50.417	62.734	37.399	26.349	23.766	0.207	FvH4_3g04370	Fvb3	2E+06	3E+06	alternative NAD(P)H dehydrogenase 1	gene30470
GENE30469	19.966	25.043	25.076	25.601	22.688	21.678	21.988	27.743	27.568	27.551	26.453	26.069	25.571	23.463	0.008	FvH4_3g04371	Fvb3	2E+06	2E+06	0	gene30469
GENE30468	26.05	27.833	32.843	36.492	28.517	29.319	34.475	22.673	27.966	30.721	39.659	33.019	37.58	35.336	0.551	FvH4_3g04372	Fvb3	3E+06	3E+06	0	gene30468
GENE30467	865.41	1742.9	2207.3	2552.9	2259.6	1244.9	974.99	1098.6	3258.1	3071.5	3838.1	3854.7	2814.1	1316	0.059	FvH4_3g04380	Fvb3	3E+06	3E+06	0	gene30467
GENE30466	565.05	479.86	681.96	481.62	751.14	419.38	269.36	464.78	886.33	921.77	1020.6	894.85	782.15	437.86	0.038	FvH4_3g04381	Fvb3	3E+06	3E+06	0	gene30466
GENE30465	164.99	155.51	239.05	192.72	163.47	123.16	161.83	124.78	165.93	138.81	197.62	179.82	216.74	203.42	0.844	FvH4_3g04390	Fvb3	3E+06	3E+06	alpha/beta-Hydrolases superfamily protein	gene30465
GENE30464	32.08	54.304	25.258	38.163	31.45	21.488	25.07	43.813	21.011	20.174	31.048	21.37	28.074	21.405	0.294	FvH4_3g04400	Fvb3	3E+06	3E+06	Homeodomain-like superfamily protein	gene30464
GENE30463	8.9103	8.1387	6.4937	6.2299	5.9878	6.7545	7.4974	6.447	8.5235	6.9497	7.4967	8.2401	7.8913	7.4527	0.404	FvH4_3g04401	Fvb3	3E+06	3E+06	0	gene30463
GENE30462	601.5	1284.6	604.61	1092.4	1606	1398.2	1559.4	1284.1	1860	1581.8	855.42	962.01	1323.8	2199.2	0.277	FvH4_3g04410	Fvb3	3E+06	3E+06	HhH-GPD base excision DNA repair family protein	gene30462
GENE30460	799.09	573.14	660.27	588.37	603.84	626.59	570.03	633.75	475.67	548.31	480.88	457.93	432.73	538.49	0.010	FvH4_3g04430	Fvb3	3E+06	3E+06	HAL2-like	gene30460
GENE30459	2001	2782.5	3249.6	2617.5	4116.2	1793.7	2336	2179.8	2091.7	2166.7	1603.6	2813.9	1857	2605.9	0.164	FvH4_3g04450	Fvb3	3E+06	3E+06	0	gene30459
GENE30458	16697	19810	16031	17188	15907	15135	16062	17761	16932	15303	16354	16003	17631	13080	0.533	FvH4_3g04440	Fvb3	3E+06	3E+06	secretory carrier 3	gene30458
GENE30457	4905.4	4117.8	5182.5	5228.1	4190.6	5716.6	4357.2	1774.2	4483.8	4065.9	1791.5	2760.3	2001.3	5673	0.035	FvH4_3g04460	Fvb3	3E+06	3E+06	Protein of unknown function (DUF707)	gene30457
GENE30456	101.4	166.65	159.33	187.78	211.28	152.23	111.83	247.56	264.44	218.54	233.27	193.73	419.5	116.49	0.052	FvH4_3g04470	Fvb3	3E+06	3E+06	bromodomain 4	gene30456
GENE30454	489.54	231.22	344.19	270.39	591.01	485.03	698.38	310.81	601.52	551.98	725.54	300.48	493.42	242.75	0.862	FvH4_3g04510	Fvb3	3E+06	3E+06	0	gene30454
GENE30453	71.404	63.898	65.864	56.73	44.068	56.581	50.228	139.41	46.126	38.888	55.193	41.664	62.793	43.339	0.854	FvH4_3g04520	Fvb3	3E+06	3E+06	0	gene30453
GENE30452	38.717	30.169	35.463	46.956	39.599	50.608	35.8	33.676	30.471	29.654	25.216	24.012	27.167	40.227	0.016	FvH4_3g04521	Fvb3	3E+06	3E+06	BED zinc finger ;hAT family dimerisation domain	gene30452
GENE30451	108.23	90.202	64.022	62.074	66.81	82.588	90.255	40.017	23.385	20.628	24.34	24.142	31.148	41.039	0.000	FvH4_3g04530	Fvb3	3E+06	3E+06	0	gene30451
GENE30450	61.745	51.734	79.067	69.938	81.02	86.892	52.722	70.597	38.957	54.051	93.731	150.82	47.871	53.441	0.816	FvH4_3g04540	Fvb3	3E+06	3E+06	0	gene30450
GENE30449	39.275	45.254	84.473	61.092	67.288	58.178	62.614	43.221	76.767	73.475	67.713	77.842	89.607	77.036	0.135	FvH4_3g04541	Fvb3	3E+06	3E+06	0	gene30449
GENE30448	181.76	153.07	412.77	225.57	317.12	321.47	440.14	397.1	246.3	478.14	503.45	293	249.54	464.26	0.189	FvH4_3g04550	Fvb3	3E+06	3E+06	lysine decarboxylase family protein	gene30448
GENE30447	24.442	22.479	33.119	87.471	32.827	93.033	19.328	72.166	18.776	23.195	58.5	43.322	28.062	23.257	0.658	FvH4_3g04560	Fvb3	3E+06	3E+06	0	gene30447
GENE30446	7357.3	5599.6	6536.1	6417.4	6031.7	5295.8	5613.2	5991.9	5001.4	5234.5	5779.9	5756.9	5336.1	4976.2	0.053	FvH4_3g04570	Fvb3	3E+06	3E+06	LisH and RanBPM domains containing protein	gene30446
GENE30445	1382.9	3007.1	2020	2210.7	2808.9	3284.3	2983.9	4578.3	3556.4	3405.7	2586.8	2019.5	4027.7	3343.7	0.068	FvH4_3g04580	Fvb3	3E+06	3E+06	SNF2 domain-containing protein / helicase domain-containing protein / zinc finger protein-related	gene30445
GENE30444	1252	1317.6	1495.1	1033.7	1619.6	1691.7	1967.5	1378.5	1501	1522.3	1507.7	1680.8	1260.4	1710.3	0.846	FvH4_3g04590	Fvb3	3E+06	3E+06	alpha/beta-Hydrolases superfamily protein	gene30444
GENE30443	30.312	54.783	28.849	29.293	28.632	35.566	55.51	32.463	38.546	30.148	45.875	61.305	32.644	27.907	0.897	FvH4_3g04600	Fvb3	3E+06	3E+06	serine carboxypeptidase-like 31	gene30443
GENE30442	18.182	21.018	18.902	18.227	19.498	20.888	20.159	19.895	16.621	17.63	19.416	24.28	19.326	18.948	0.918	FvH4_3g04610	Fvb3	3E+06	3E+06	cytokinin oxidase 7	gene30442
GENE30441	27.98	32.625	33.219	26.51	34.305	32.447	38.644	24.829	39.277	40.649	34.651	62.814	37.612	53.377	0.092	FvH4_3g04611	Fvb3	3E+06	3E+06	0	gene30441
GENE30440	10.806	14.046	15.844	14.746	13.614	14.515	13.739	14.121	16.189	16.566	17.702	14.304	12.449	14.464	0.201	FvH4_3g04620	Fvb3	3E+06	3E+06	serine carboxypeptidase-like 31	gene30440
GENE30439	10890	9371.1	11076	11306	9358	8908.5	9623.9	8411.3	8566.3	9908	8912.7	8202.2	10984	10666	0.242	FvH4_3g04630	Fvb3	3E+06	3E+06	NAC domain containing protein 25	gene30439
GENE30438	24.473	22.853	28.55	26.208	25.864	30.387	24.346	30.066	28.827	24.944	25.039	22.779	27.91	20.777	0.839	FvH4_3g04640	Fvb3	3E+06	3E+06	Toll-Interleukin-Resistance (TIR) domain family protein	gene30438
GENE30437	25.026	18.729	22.996	20.242	24.048	20.988	22.063	21.812	22.624	19.976	19.47	18.247	21.59	20.699	0.200	FvH4_3g04641	Fvb3	3E+06	3E+06	0	gene30437
